# Ab initio study of mechanical and thermal properties of GeTe-based and PbSe-based high-entropy chalcogenides

**DOI:** 10.1038/s41598-023-42101-5

**Published:** 2023-09-27

**Authors:** Sahib Hasan, Puja Adhikari, Saro San, Wai-Yim Ching

**Affiliations:** 1 Department of Physics and Astronomy, University of Missouri-Kansas City, Kansas City, MO 64110 USA; 2 Department of Sciences, College of Basic Education, Al Muthanna University, Samawah, 66001 Iraq

**Keywords:** Materials science, Condensed-matter physics, Theory and computation

## Abstract

GeTe-based and PbSe-based high-entropy compounds have outstanding thermoelectric (TE) performance and crucial applications in mid and high temperatures. Recently, the optimization of TE performance of high-entropy compounds has been focused on reducing thermal conductivity by strengthening the phonon scattering process to improve TE performance. We report a first-principles investigation on nine GeTe-based high-entropy chalcogenide solid solutions constituted of eight metallic elements (Ag, Pb, Sb, Bi, Cu, Cd, Mn, and Sn) and 13 PbSe-based high-entropy chalcogenide solid solutions: Pb_0.99-y_Sb_0.012_Sn_y_Se_1-2x_Te_x_S_x_ (x = 0.1, 0.2, 0.25, 0.3, 0.35, 0.4, 0.45, and y = 0) and Pb_0.99-y_Sb_0.012_Sn_y_Se_1-2x_Te_x_S_x_ (y = 0.05, 0.1, 0.15, 0.2, 0.25 and x = 0.25). We have investigated the mechanical properties focusing on Debye temperature (*Θ*_D_), thermal conductivity (*κ*), Grüneisen parameter *(γ*_α_*)*, dominant phonon wavelength (*λ*_dom_), and melting temperature (*T*_m_). We find that the lattice thermal conductivity is significantly reduced when GeTe is alloyed into the following compositions: Ge_0.75_Sb_0.13_Pb_0.12_Te, Ge_0.61_Ag_0.11_Sb_0.13_Pb_0.12_Bi_0.01_Te, and Ge_0.61_Ag_0.11_Sb_0.13_Pb_0.12_Mn_0.05_Bi_0.01_Te. This reduction is due to the mass increase and strain fluctuations. The results also show that Ge_0.61_Ag_0.11_Sb_0.13_Pb_0.12_Bi_0.01_Te solid solution has the lowest Young’s modulus (30.362 GPa), bulk and shear moduli (18.626 and 12.359 GPa), average sound velocity (1653.128 m/sec), Debye temperature (151.689 K), lattice thermal conductivity (0.574 W.m^–1^.K^–1^), dominant phonon wavelength (0.692 Å), and melting temperature (535.91 K). Moreover, Ge_0.61_Ag_0.11_Sb_0.13_Pb_0.12_Bi_0.01_Te has the highest Grüneisen parameter with a reduced and temperature-independent lattice thermal conductivity. The positive correlation between *Θ*_D_ and *κ* is revealed. Alloying of PbSe-based high-entropy by Sb, Sn, Te, and S atoms at the Se and Pb sites resulted in much higher shear strains resulted in the reduction of phonon velocity, a reduced *Θ*_D_, and a lower lattice thermal conductivity.

## Introduction

High-entropy alloys (HEAs) are alloys with high configuration entropy obtained by increasing the number of constituting elements (*n*) with *n* ≥ 5^1^. The atomic concentration of elements in high-entropy (HE) materials can be between 5 and 35% of the samples^2, 3^. The concept of HE originated from the hypothesis that the solid solution is stabilized by a high configurational entropy of mixing^4^. Alternative criterion to define a HE material is the value of entropy of mixing ΔS_*mix*_ expressed as $\Delta {S}_{mix}=-R\sum_{i=1}^{N}{x}_{i}ln{x}_{i}$, where *R*, *N*, and *x*_*i*_ are the gas constant, number of components, and atomic fraction of the components, respectively^5^. Materials can be classified as low-entropy (Δ*S*_mix_ less than 0.69R), medium-entropy (Δ*S*_mix_ between 0.69R and 1.59R) and high-entropy (Δ*S*_mix_ larger than 1.60R)^6^. The synthesis of HE materials is an emerging field of research which aims to design multicomponent single-phase materials. The multicomponent materials contain a minimum of 5 elements in nearly equal atomic ratios or not so equal in high-entropy metal alloys (HEMAs). HEMAs have a wide range of remarkable mechanical^7, 8^, dielectric^9^, and superconducting^10^ properties. Recently, researchers found that HE materials can be disordered with severe lattice distortions and non-diffusion characteristic of the atoms contained therein exhibiting low thermal stability^11^. The scattering of phonons increases due to the severe lattice distortion, thus reducing lattice thermal conductivity of HE materials^12^. Single phase HEAs possess high hardness^13^, high strength^14^, good wear^15^, and erosion resistance^16^. Other multicomponent compounds, such as high-entropy oxides (HEOs)^17, 18^, borides^19^, and carbides^20, 21^ have also been synthesized recently.

Despite the intense research on HE materials, HE materials with semiconducting characteristics have not yet been explored. HE materials having a band gap have a wide range of electronic, thermoelectric, and optical applications^22–24^. The alloying of semiconductors has been employed to enhance the electronic, structural, and other functional characteristics, resulting in many emergent properties. For example, some alloyed semiconductors such as chalcogenides exhibit low thermal conductivity, tunable optical emission, and higher number of carrier mobility^25–28^. Chalcogenides with semiconducting characteristics contain at least one of the elements: tellurium (Te), selenium (Se), and sulfur (S). In particular, the family of IV − VI compounds have attracted a great attention due to their optoelectronic, microelectronic, and high thermoelectric (TE) performance^29, 30^. Recently, the most investigated category of IV − VI semiconductor chalcogenides are: SnS^31^, SnSe^32^, SnTe^33^, GeSe^34^, GeTe^35^, PbS^36^, PbSe^37^ and PbTe^38^.

The direct conversion of heat into electricity under a temperature gradient is carried out by thermoelectric (TE) devices. TE technology is one of the promising green solutions to mitigate the energy and environment crisis^39^. The performance of TE devices is described by the parameter *ZT* called figure of merit defined as *ZT* = *S*^2^*σT/κ*, where *σ* is the electrical conductivity, *S* is the Seebeck coefficient, *T* is the absolute temperature, and *κ* is the thermal conductivity. To improve the TE performance of chalcogenides, doping and alloying have been applied recently. For example, TE performance of Bi_2_(Te,Se)_3_, Sn(S,Se), and GeTe alloys has shown to be enhanced^40–44^. The attempts to improve TE performance, by increasing TE power factor (PF) and lowering the lattice thermal conductivity (*κ*_*L*_) have skyrocketed. Other attempts include introducing the configurational entropy via doping or alloying techniques^45^. For examples, chalcogenide compounds Ge_0.84_In_0.01_Pb_0_._1_Sb_0.05_Te_0.997_I_0.003_^29^, PbSnTeSe^46^, and (Sn_0.7_Ge_0.2_Pb_0.1_)_0_._75_Mn_0.275_Te^47^ achieved the enhancement of TE performance. Apparently, this strategy has been successful^48^. The sluggish diffusion and sever lattice distortion^49^ in HE materials have induced many fascinating TE properties such as low *κ*_*L*_ and very high *ZT*^50, 51^. HEA are widely used to enhance TE performance in many chalcogenide systems such as (SnGePbMn)Te^47^, BiSbTe_1.5_Se_1.5_^52^, and (PbTe)_1−2x_(PbSe)_x_(PbS)_x_^53^. A high figure of merit (*ZT* = 1.42 at 900 K) was achieved^47^ in (Sn_0.74_Ge_0.2_Pb_0.1_)_0.75_Mn_0.275_Te.

Among chalcogenide materials, germanium telluride GeTe^54^ is a promising semiconductor with a narrow band gap and good TE performance. GeTe belongs to the IV-VI group and has rhombohedral crystal structure (*R*3*m*) at room temperature^55, 56^. It exhibits a sudden phase transition to the cubic rock-salt structure (*Fm*-3* m*) at around 700 K^57^. Compared to PbTe and SnTe, GeTe is much less explored. Pure GeTe has average TE properties with maximum *ZT* of less than 0.8 at 720 K^58^. Electronic structure, dynamical, dielectric, and elastic properties of pure GeTe was investigated using density functional perturbation theory by R. Shaltaf et al.^59^. They used the Hartwigsen-Goedecker-Hutter pseudopotentials including spin–orbit coupling to achieve high accuracy of the calculations. They found that GeTe is a semiconductor with a direct energy band gap of 0.48 eV. However, alloyed and doped rhombohedral crystals of GeTe with high TE performance have been reported^60, 61^ revealing its reduced lattice thermal conductivity and higher *ZT* (*ZT ≈* 2.2). This was achieved in Ge_1–x–y_Sb_x_In_y_Te^62^, Ge_1–x–y_Sb_x_ Zn_y_Te^63^, and Ge_1–x–y_Bi_x_Cd_y_Te^64^. Other attempts were carried out to reduce the phase transition temperature of GeTe by dual-doping of Sb and Mn^57^, and Bi and Mn^65^. Thus, TE performance of alloyed and doped GeTe was enhanced in the medium temperature range of 700 K. By introducing Ag, Sb, Pb, and Bi to Ge sites to form a high-entropy GeTe-based material, higher electrical transport and lower thermal conductivity were achieved by Jiang et al.^66^.

Lead chalcogenides PbX (X = S, Se and Te) have been extensively studied in the last decades due to their applications as TE materials available at medium temperature (400–900 K)^67–70^. They belong to the IV-VI group and have a simple NaCl-type (B1) structure at ambient conditions. They are narrow gap semiconductors having low thermal conductivities even at high temperatures (500–700 k)^71^. This makes them good TE materials. TE performance of PbSe was enhanced by doping with elements such as B, Ga, In, Tl, Pb^72, 73^, Al^74^, Sb^75^, and Bi^76^. HE technique has also been used to achieve high TE performance of PbSe-based HE compounds. Jiang et al. studied TE performance of Pb_0.89_Sb_0.012_Sn_0.1_Se_0.5_Te_0.25_S_0.25_^77^, Pb_0.975_Na_0.025_Se_0.5_S_0.25_Te_0.25_, and Pb_0.935_Na_0.025_Cd_0.04_Se_0.5_S_0.25_Te_0.25_ solid solutions^78^. They exhibit high *ZT* and low thermal conductivity.

Obviously, optimization of TE performance via the minimization in lattice thermal conductivity (*κ*_L_) has skyrocketed. Creating a strong phonon scattering via phonon engineering is one of the best ways to minimize *κ*_L_. This can be achieved by replacing light atoms by heaver atoms^79, 80^, creating point defects^81^, enhancing strong lattice anharmonicity^82^, and producing dislocations^75, 83^. Thus, manipulation of the phonon dispersion is the key to reduce *κ*_*L*_. The phonon dispersion in can be expressed as in Eq. (1)^84^:

1 $$\begin{array}{c}\omega =2\sqrt{\frac{F}{M}} \mathrm{sin}\left(\frac{\pi }{2}\frac{k}{{k}_{c}}\right),\end{array}$$

where *F*, *M*, *k*, and *k*_*c*_ are the force constant, atomic mass, wave vector, and cut-off wave vector, respectively. Any manipulation of phonon dispersion can be carried out through the changes in these parameters. Low *ω* requires a large *M* but a small *F* (weak chemical bonds) which results in a low *κ*_L_. Anharmonic lattice vibrations (anharmonicity) is connected to *F* which can be manipulated by using the elastic lattice strains, or the change to non-equilibrium atomic positions from its equilibrium position. While *M* can be changed through substitution, removal, or insertion of atoms. Clearly, a significant reduction in *κ*_L_ can be carried out by creating a large change in *M*. In the current study, we focus on the large change in *M* through alloying to create a significant reduction in *κ*_L_.

There are recent studies of GeTe-based HE^66^ and PbSe-based HE^77^ focusing in their development and applications. Jiang et al.^66, 77^ pointed out on their potential of very high *ZT.* In Jiang’s work^66^, nine GeTe-based HE solid solutions were synthesized, then TE properties such as *κ*_*L*_ and *ZT* were experimentally determined. Density functional theory (DFT) calculations were carried out for small models with few atoms (16 atoms) by using special quasi-random structure (SQS)^85^. They calculated several mechanical parameters such as Young's modulus, shear modulus, Poisson’s ratio, and Grüneisen parameter. The purpose of using SQS approach is to simulate a random solid solution using a relatively very small supercell to reduce the computational cost. However, to efficiently mimic the chemical disordering of multi-principal element in a random solid solution, using a large cell is required. Jiang et al. missed analyzing the effect of Grüneisen parameter on lattice thermal conductivity and calculating several crucial parameters such as Kleinman parameter, machinability index, Debye temperature, minimum thermal conductivity, and melting temperature.

In this study, we expand Jiang et al.’s work to fill the gap by investigating the elastic and thermal properties of pure GeTe(m0) and nine randomly disordered solid solutions of GeTe-based HE chalcogenides. They are listed in Fig. S1a, Tables S1 and S2 (shown in the supplementary information (SI)). In addition, we have investigated the pure PbSe and thirteen PbSe-based HE chalcogenide solid solutions: Pb_0.99-y_Sb_0.012_Sn_y_Se_1-2x_Te_x_S_x_ (x = 0.1, 0.2, 0.25, 0.3, 0.35, 0.4, 0.45, and y = 0) and Pb_0.99-y_Sb_0.012_Sn_y_Se_1-2x_Te_x_S_x_ (y = 0.05, 0.1, 0.15, 0.2, 0.25 and x = 0.25) as listed in Fig. S2b, Tables S3 and S4. The results are presented in “Results” section below. GeTe-based HE solid solution models each with 1080 atoms were generated from the simple rhombohedral crystal (space group *R*3*m*) using the supercell (SC) method^86^. Elements Ag, Sb, Pb, Bi, Cd, Cu, Sn, and Mn are distributed randomly in these nine HE models (m1 to m9). For PbSe-based HE models, thirteen solid solutions are modeled from single-phase rock-salt structure (space group fcc) with supercells each consisting of 1000 atoms. Next, the elements Sb, Sn, Te, and S are distributed randomly in these thirteen HE models. Large supercell for such HE chalcogenides are constructed for the first time in this work. Our calculations are performed using DFT-based package, *Vienna *Ab initio Simulation Package (VASP). The current methods do not consider the effect of temperature. Nevertheless, the configurational entropy is captured using randomly generated supercells. More details of the computational methods used can be found in the supplementary information (SI). The results are presented in “Results” section and discussed in “Discussion” section. Figure S1a shows Ball and stick structure of Ge_0.61_Ag_0.11_Sb_0.13_Pb_0.12_Bi_0.01_Te (m5) with their solid solution composition and model numbers in the box at right, and (b) shows Ball and stick structure of Pb_0.99_Sb_0.012_Se_0.5_Te_0.25_S_0.25_ with their solid solution composition in the box on the right.

## Results

### Elastic constants of GeTe-based high-entropy chalcogenides

The elastic constants correlate stress to strain behavior. Elastic constants describe the response of materials under external forces. In this work, we calculated the mechanical properties of m0 and the nine GeTe-based high-entropy solid solutions models using VASP. Details of the method are described in the SI. The calculated elastic constants (C_ij_) are listed in Table S5. They provide information about the stability, stiffness, brittleness, ductility, and anisotropy of materials. Based on the calculated C_ij_ values, m0 and nine solid solutions fulfill the mechanical stability criterial for trigonal structure^87^ (${C}_{11}>{C}_{12}$, ${C}_{44}>0$, ${C}_{13}^{2}<\frac{1}{2}{C}_{33}({C}_{11}+{C}_{12})$, ${C}_{14}^{2}<\frac{1}{2}{C}_{44}({C}_{11}-{C}_{12})\cong {C}_{44}{C}_{66}$). The elastic constants C_11_, C_22_, and C_33_ are strongly correlated with the unidirectional compression along the principal x, y, and z directions^88^ and are very close in cubic structures. Synonymously, C_11_, C_22_, and C_33_ describe the resistance of a material against the deformation along [100], [010], and [001] directions, respectively, whereas the *C*_44_ and *C*_66_ measure the resistance against the shear deformation in the (100) and (001) planes, respectively. Large values of C_11_, C_22_, and C_33_ indicate incompressibility under uniaxial stress along x, y or z axes, respectively.

Our calculations show C_11_ and C_22_ are remarkably close in all nine solid solution models which is normal since the lattice parameters *a* and *b* are equal in the trigonal structures. The C_11_ and C_22_ for all nine solid solutions are larger than C_33,_ indicating that they are more compressible under uniaxial stress along z direction than x and y directions. It also means that the bonding strength along x and y axes are stronger than along the z axis. C_66_ is higher than C_44_ in m0, m1, m3, m5, and m8 indicating that the shear along the (100) plane is easier relative to the shear along the (001) plane. C_44_ is higher than C_66_ in m2 and m9 indicating that the shear along the (001) plane is easier relative to the shear along the (100) plane. C_66_ is higher than C_44_ in m4, m6, and m7. A low value for *C*_44_ indicates a high shearability. Due to the lowest *C*_44_ value, m5 has the highest shearability among all other solid solutions. The values of the elastic constants generally depend on the lattice parameters and the strength of the bonds between the elements. Comparing m0 (pure GeTe) with models m1 to m9, we see that introducing Ag, Sb, Pb, Bi, Cu, Mn, Cd, and Sn elements at the Ge sites led to reduction in C_11_ and C_22_. Introducing Sb and Pb in m2; Ag, Sb, and Pb in m4; Ag, Sb, Pb, and Bi in m6; Ag, Sb, Pb, Bi, and Cu in m7; Ag, Sb, Pb, Bi, and Sn in m9 all resulted in higher value of C_33_. Introducing Ag, Sb, Pb, and Bi in Ge sites of m5 results in much reduced elastic constants in comparison with m0, which implies that the elastic strains in m5 are much higher. Reduction of elastic strains influences their thermal conductivity.

Materials can resist the external applied stress by two ways: bond bending and bond stretching/contracting. The dimensionless Kleinman parameter (ζ)^89^ can be used to determine the contribution of the bond bending or the bond stretching/contracting to resist the external stress. More information about the formula used to calculate ζ is shown in Eq. (S10). Kleinman parameter is a way to measure the stability of a material against the bending or stretching. ζ lies in the range 0 ≤ ζ ≤ 1. The lower limit of ζ indicates insignificant contribution to bond bending, whereas the upper limit of ζ means the insignificant contribution of bond stretching/contracting to resist external applied stress. The calculated ζ for m0 and the nine solid solutions models are shown in Table 1. From these values, we predict that the mechanical strength in m0 and nine solid solutions is dominated by the bond bending comparing to bond stretching/contracting.

**Table 1:** Young’s modulus (E), bulk modulus (K), shear modulus (G), Poisson’s ratio (η), Pugh’s ratio (G/K), Vicker’s hardness (*H*_V_), micro-hardness (*H*), Kleinman parameter (ζ), and machinability index(*µ*_M_) for ten models in Ge-Te based high-entropy chalcogenides.

Model	E (GPa)	K (GPa)	G (GPa)	η	G/K	*H*_V_ (GPa)	*H* (GPa)	ζ	µ_M_
m0	59.716	35.148	24.537	0.217	0.698	5.892	4.629	0.377	1.309
m1	41.484	33.447	16.038	0.293	0.480	2.849	2.214	0.539	2.217
m2	42.489	36.976	16.236	0.309	0.439	2.596	2.067	0.469	1.372
m3	47.631	32.712	18.941	0.257	0.579	3.964	3.069	0.467	1.702
m4	53.582	38.885	21.090	0.270	0.542	3.967	3.235	0.480	1.492
m5	30.362	18.626	12.359	0.228	0.664	3.425	2.242	0.433	1.741
m6	52.784	38.819	20.726	0.273	0.534	3.855	3.138	0.484	1.490
m7	52.211	38.772	20.466	0.276	0.528	3.773	3.055	0.494	1.510
m8	42.853	33.805	16.626	0.289	0.492	3.005	2.338	0.493	1.725
m9	54.603	40.317	21.425	0.274	0.531	3.922	3.229	0.478	1.400

The other mechanical parameters such as bulk modulus (K), Young’s modulus (E), shear modulus (G), and Poisson’s ratio (η) are obtained from the elastic coefficient C_ij_ and the compliance tensor S_ij_ (S_ij_ = 1/C_ij_) using Voigt–Reuss–Hill (VRH) approximation^90, 91^ for poly-crystals. They are listed in Table 1. Young’s modulus measures the stiffness of the materials or change in length. Bulk modulus gives information about the resistance to compressibility or change in volume under pressure. Shear modulus represents the resistance against shear distortion. Figure S2a–d shows the distribution of K, E, G and Poisson’s ration (η) of m0 and the nine HE solid solutions. m5 has the smallest K, E and G moduli. In general, introducing Ag, Sb, Pb, Bi, Cu, Mn, Cd, and Sn elements at the Ge sites results in reduced E and G. Introducing Sb and Pb in m2; Ag, Sb, and Pb in m4; Ag, Sb, Pb, and Bi in m6; Ag, Sb, Pb, Bi, and Cu in m7; Ag, Sb, Pb, Bi, and Sn in m9 resulted in higher bulk modulus comparing with m0. Eight out of the nine HE solid solutions have bulk modulus higher than 30 GPa except m5. Hence, m5 is relatively compressible in nature.

Machinability is the ease with which the materials can be machined at a low cost. The bulk modulus K together with *C*_44_ can control the machinability also known as machinability index (µ_M_) shown in Eq. (S11). Calculating machinability index is crucial to evaluate applications in different materials. Higher µ_M_ indicates higher machinable characteristics. The machinability index is also a way to measure the plasticity and lubricating nature of a material^92^. It is obvious that large µ_M_ requires a small C_44_ which can give a better dry lubricity. Large value of µ_M_ leads to lower friction and higher plastic strain. Solid solutions models m1, m5, and m8 have the largest values of µ_M_ among others as shown in Table 1. The ratio of shear modulus to bulk modulus (G/K), or Pugh’s modulus ratio^91, 93^ is an useful parameter that determines the brittle and ductile behaviors of materials. G/K for m0 and other nine HE solid solution models are listed in Table 1 and shown in Fig. S3a. According to Pugh’s criterion, materials with G/K larger than 0.571 tend to be brittle and those less than 0.571 tend to be ductile^93, 94^. Similarly, m0, m3, and m5 are brittle while the remaining models are more ductile. Another rule to characterize material’s brittleness or ductility is the Frantsevich’s rule of Poisson’s ratio^95^. It assumes that if Poisson’s ratio (η) is less than 0.26, the material tends to be brittle otherwise, it is ductile. Hence, Frantsevich’s rule and Pugh’s criterion are equivalent.

We also used Vicker’s hardness or macro hardness (*H*_V_) formula to calculate the hardness (*H*_V_) of m0 and other nine solid solutions models. Vicker’s hardness formula was formulated by Chen et al*.*^96^ and Tian et al.^97^. Vicker’s hardness formula was derived from the elastic constants^98–100^ using Eq. (S12). The calculated values of *H*_V_ for m0 and all nine solid solution models are listed in Table 1. Figure S3b shows the distribution of the Vicker’s hardness of m0 and the nine HE solid solutions. Materials with *H*v larger than 40 GPa are presumed to be super hard materials^101^. *H*_V_ of the 10 models listed in Table 1 have values less than 10 GPa and cannot be considered as hard materials. Historically, Macro Vickers loads vary from 2 to 120 kg. However, when the applied loads range from a few grams to several kilograms, micro-hardness (*H*) measurements are more suitable^102, 103^. A semi-empirical formula that can estimate the micro-hardness (*H*) is listed in Eq. (S13). The values of *H* are listed in Table 1. Both macro hardness (*H*_V_) and micro hardness (*H*) have the same trend and both of them are crucial parameters to be calculated for the chalcogenide models. From the above results, introducing Ag, Sb, Pb, Bi, Cu, Mn, Cd, and Sn elements at the Ge sites results in reduced brittleness and hardness of the nine solid solution models.

Another popular parameter used to classify the chemical bonding of a material is the Cauchy pressure (CP)^104, 105^. CP for trigonal structures can be calculated using following formula: CP_x_ = *C*_13_–*C*_44_ and CP_y_ = *C*_12_–*C*_66_^104^. A negative CP indicates dominance of covalent bonding and a positive CP indicates dominance of ionic bonding^106^. As shown in Table S6, the covalent bonding is dominant in m0 and m5 and the ionic bonding is dominant in m1, m7, and m8. The remaining models (m2, m3, m4, m6, and m9) have mixed nature of both ionic and covalent bonding due to the opposite signs of CP_x_ and CP_y_. Cauchy pressure can also be used to predict the brittle/ductile behavior of materials. Negative CP implies that the material is brittle. m0, m3, and m5 have negative CP so they are brittle. This result is in agreement with Pugh’s criterion. The elasticity’s response was also investigated by us through the Lame’s constants (*λ*, *μ*). Lame’s constants can be used to measure the compressibility and the shear stiffness of a material, respectively. In most cases, and in the context of elasticity, *μ* carries the same information as shear modulus. The formula used to calculate *λ* and *μ* can be found in Eqs. (S14) and (S15). *λ* can be negative, while *μ* is always positive. The values of *λ* and *µ* at zero temperature and zero pressure are listed in Table S6. As can be seen, m0 and m5 exhibit shear stiffness than compressibility, while the other solid solutions models exhibit opposite trends.

The elastic anisotropy parameter is characterized by the universal anisotropic index (*A*^*U*^) calculated using formula (S16), listed in Table S7, and shown in Fig. S3c. If *A*^*U*^ has a value of unity, the material is isotropic, while values other than unity give varying degrees of anisotropy^107^. The *A*^*U*^ for m0 and all nine models vary from unity in different degrees, showing their anisotropic nature. Comparatively, m3 has *A*^*U*^ close to 1 showing isotropic nature. Another way of measuring the elastic anisotropy is the percentage of anisotropy in compression (*A*_comp_) and shear (*A*_shear_) listed in Table S7. If* A*_comp_ and *A*_shear_ have a value of zero, it indicates isotropic elastic behavior whereas their value of 1 indicates largest possible anisotropy. The shear anisotropic factor for {100} shear planes between the [011] and [010] directions (*A*_*1*_), the shear anisotropic factor for {010} shear planes between the [101] and [001] directions (*A*_*2*_), and the shear anisotropic factor for {001} shear planes between the [110] and [010] directions (*A*_*3*_) are calculated by the formulae (S19), (S20) and (S21). The values of *A*_*1*_, *A*_*2*_, and *A*_*3*_ for m0 and nine solid solution models are presented in Table S7. If *A*_*1*_ = *A*_*2*_ = *A*_*3*_ = 1, the material is isotropic and bonds existing between the planes, otherwise, the opposite is true, or anisotropic nature for all models.

The directional dependent mechanical properties, E, K in terms of compressibility (1/K), G, and η, are presented in three-dimensional (3D) surface plots via ELATE program^108^ in Figs. S4–S7 respectively. The isotropic compounds exhibit a perfect spherical in 3D plot. The 3D surface plot of E (Fig. S4) and G (Fig. S6) shows m0 and all nine solid solution models are anisotropic nature. m1 and m5 have less deviation while m2 has the largest deviation from the spherical shape for both E and G, indicating that m2 has the highest Young and shear anisotropy while m1 and m5 have the least. An extreme anisotropy in the compressibility (inversely proportional to the bulk modulus) is notable in all nine solid solution models. The circular graphic in 2D for elastic moduli gives the same information about the isotropic nature of solids. The amount of deviation from the circular shape determines the degree of anisotropy. Figures S8–S17 show the two-dimensional (2D) plots of E, 1/K, G, and η for m0 and other nine solid solutions. Figures S8–S17 show that E and 1/K are found to be isotropic in the xy plane while they are anisotropic in xz and yz planes for all models. The minimum and maximum values of each mechanical modulus derived by using ELATE program are listed in Table S8.

### Debye temperature and thermal conductivity of GeTe-based high-entropy chalcogenides

**Figure 1:**
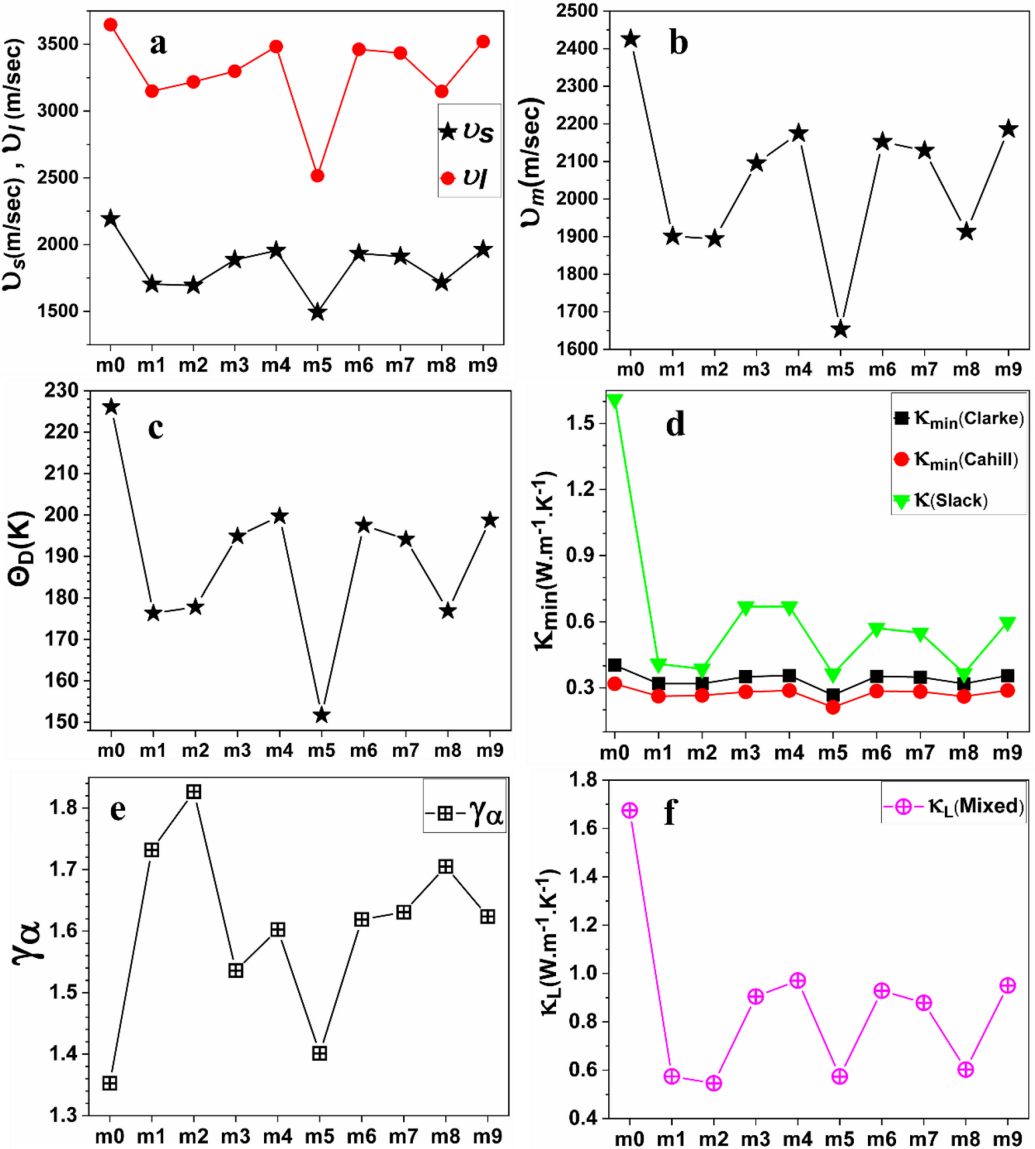
(**a**) Longitudinal *ν*_*l*_ and transverse *ν*_*s*_ velocities, (**b**) Average sound velocity, (**c**) Debye temperature, (**d**) thermal conductivities at 300 K, (**e**) Acoustic Grüneisen constant, and (**f**) Lattice thermal conductivities calculated according to mixed model at 300 K for the ten models in Ge-Te based high-entropy chalcogenides.

Debye temperature (*Θ*_D_) originates from the theory of thermal vibration of atoms. *Θ*_*D*_ in condensed materials can reflect the strength of covalent bonding and other thermal characteristics such as specific heat, melting temperature, and thermal expansion. *Θ*_*D*_ is positively correlated with thermal conductivity (*κ*) and is an important parameter in high temperature applications. Anderson’s method is one of the straightforward and accurate methods to calculate *Θ*_*D*_. The formula used to calculate *Θ*_*D*_ is shown in Eq. (S22). Other parameters used to calculate *Θ*_D_, such as the average sound velocity (*v*_m_), the transverse (shear) velocity (*v*_s_), and the longitudinal sound velocity (*v*_l_) are shown in Eqs. (S23), (S24) and (S25) respectively. The calculated *ν*_s_, *ν*_l_, *ν*_m_, and *Θ*_D_ for m0 and the nine solid solution models are listed in Table 2 and plotted in Fig. 1a–c. We notice that m0 has the highest *Θ*_D_ and* v*_m_, while m5 has the smallest *v*_m_ and *Θ*_D_. This indicates that m5 has the weakest bond strength and lowest thermal conductivity. m1, m2, and m8 have close values of *v*_m_ and *Θ*_D_. Materials with low *Θ*_D_ tend to be soft materials with low melting temperature. While materials with higher *Θ*_D_ tend to be harder, with stronger interatomic bonding and higher melting temperature^109, 110^. From Table 2, we notice that the transverse sound velocity *ν*_s_ is depressed in all nine solid solution models (form m1 to m9) in comparison to pure GeTe (m0). m2 and m5 have the smallest values of *ν*_s_. This significantly dampened transverse phonon modes (*ν*_*s*_) would strengthen the scattering of phonons^111^ which in return results in a reduced lattice thermal conductivity (*κ*_L_). Impedance parameter (Z) is used in acoustic applications such as noise reduction and transducer design. In this work, Z is calculated using Eq. (S26) and listed in Table 2. When sound waves are transmitted, the difference in *Z* between two materials determines the amount of acoustic energy transmitted at their interface.

**Table 2:** The theoretical density (*ρ*), the calculated sound velocity (longitudinal *ν*_l_, transverse *ν*_s_, and average *ν*_m_), Debye temperature *Θ*_D_, and the acoustic impedance (*Z*) in Kg m^–2^ s^–1^ unit for ten models in Ge-Te based high-entropy chalcogenides.

Model	ρ (Kg/m^3^)	*ν*_*s*_ (m/sec)	*ν*_l_ (m/sec)	*ν*_m_ (m/sec)	*Θ*_D_ (*K*)	*Z* (× 10^6^)
m0	5100.85	2193.257	3647.526	2425.743	226.102	11.187
m1	5526.86	1703.475	3149.734	1901.120	176.298	9.415
m2	5658.40	1693.919	3218.777	1894.103	177.805	9.585
m3	5326.40	1885.752	3298.924	2095.333	194.876	10.044
m4	5521.33	1954.413	3483.628	2175.012	199.725	10.791
m5	5546.19	1492.775	2515.852	1653.128	151.689	8.279
m6	5545.39	1933.267	3461.732	2152.272	197.491	10.721
m7	5600.65	1911.601	3434.394	2128.719	194.132	10.706
m8	5651.86	1715.135	3146.978	1913.041	176.884	9.694
m9	5558.50	1963.277	3520.297	2185.922	198.754	10.913

Thermal conductivity (*κ*) measures the performance of heat transfer at high temperatures^112^. It is crucial to calculate the minimum thermal conductivity (*κ*_min_) and lattice thermal conductivity (*κ*_L_) since they determine the TE performance. Clarke’s model^113^, Cahill’s model^114^, and Slack’s model^115^ were used to estimate *κ*_min_ and *κ* of m0 and other nine HE chalcogenide solid solutions at 300 K. The formulae (S27), (S28), (S29) and (S31) were used to calculated *κ*_min_, *κ*, and Grüneisen constant *(γ*_*α*_*)* respectively. They are listed in Table 3. Figure 1d shows that m5 has the lowest *κ*_*min*_ according to Clarke’s model and Cahill’s model, while m2, m5, and m8 have the lowest and comparable values of *κ* according to Slack’s model. The Grüneisen constant ${\gamma }_{\alpha }$ provides information about the nature of materials by measuring the bond anharmonicity, which is related to the interatomic interaction^116^. If *γ*_*α*_ is large, the anharmonic vibrations are strong, indicating a small influence of temperature increase on the lattice dynamics and thermal properties. The calculated *γ*_*α*_ for m0 and other nine HE chalcogenide solid solutions are summarized in Table 3 and Fig. 1e. With the lowest *γ*_*α*_, m0 and m5 are the highest influenced under high temperature (higher and temperature-dependent lattice thermal conductivity) whereas m2 with highest *γ*_*α*_ is less influenced under high temperature (depressed and temperature-independent lattice thermal conductivity). However, strong anharmonic vibrations (higher* γ*_*α*_) also indicate higher phonon scattering and thus low *κ*. Hence, according to this criterion, m5 should have the highest value of *γ*_*α*_ among other models. Another formula that estimates Grüneisen parameter using the Poisson’s ratio is shown in Eq. (S32). Poisson’s ratio can be calculated using the transverse (*v*_*s*_) and the longitudinal (*v*_*l*_) sound velocities as shown in Eq. (S33). *v*_*s*_ and *v*_*l*_ can also be estimated using the elastic constants as shown in Eqs. (2) and (3) below^117^:

**Table 3:** The calculated minimum thermal (*κ*_min_) and thermal (*κ*) conductivities (W⋅m^–1^. K^–1^) at 300 K, the constant *A* (*A* here is calculated by using the Julian’s formula (S34)), Slack’s thermal conductivity (*κ*_A_) calculated by using the constant *A* in the fifth column, Grüneisen parameter (*γ*_a_) calculated by using the formula (S31), and Grüneisen parameter calculated by using the formula (S32) for ten models in Ge-Te based high-entropy chalcogenides.

Model	Clarke model *κ*_min_ (W.m^–1^.K^–1^)	Cahill model *κ*_min_ (W.m^–1^.K^–1^)	Slack model κ (W.m^–1^.K^–1^)	*A* (× 10^–6^)	Slack model *κ*_A_ (W.m^–1^.K^–1^)	*γ*_α_ Eq. (S31)	*γ*_α_ Eq. (S32)
m0	0.4028	0.3185	1.610	3.2632	1.695	1.3526	1.6424
m1	0.3193	0.2620	0.409	3.1190	0.411	1.7319	2.1978
m2	0.3193	0.2652	0.386	3.0869	0.384	1.8266	1.3132
m3	0.3505	0.2815	0.669	3.1897	0.688	1.5358	1.8898
m4	0.3560	0.2878	0.670	3.1645	0.683	1.6026	1.4747
m5	0.2670	0.2119	0.364	3.2437	0.381	1.4011	2.2736
m6	0.3520	0.2851	0.571	3.1586	0.582	1.6189	1.4439
m7	0.3486	0.2828	0.550	3.1528	0.559	1.6307	1.4561
m8	0.3190	0.2609	0.366	3.1271	0.369	1.7049	1.6818
m9	0.3558	0.2883	0.599	3.1567	0.609	1.6237	1.3085

2 $$\begin{array}{c}{v}_{l}=\sqrt{\frac{{C}_{11}}{\rho }},\end{array}$$

3 $$\begin{array}{c}{v}_{s}=\sqrt{\frac{{C}_{44}}{\rho } },\end{array}$$

The calculated *v*_*l*_ and *v*_*s*_ (using Eqs. (2) and (3) respectively), and η (using Eq. (S33)) are listed in Table S9. The calculated *γ*_*α*_ (using Eq. (S32)) are listed in Table 3. m5 has the highest *γ*_*α*_ which agrees well with its lowest value of *κ*.

In Fig. S18, we plotted the *v*_*l*_/*v*_*s*_ versus Grüneisen parameter, showing that Gruneisen parameter is linearly proportional to *v*_*l*_/*v*_*s*_. The increasing *v*_*l*_/*v*_*s*_ on x-axis implies a decrease in *v*_*s*_. The decrease in *v*_*s*_ from the softened transverse phonons is the reason for the increase of *γ*_*α*_ (Eq. S31)_*.*_ Thus, the anharmonicity (*γ*_*α*_), which describes the interactions among the different branches of phonons, is largely strengthened, resulting in a temperature-independent and significantly reduced *κ*_*L*_. Thermal conductivity (*κ*) calculated by Slack’s formula (Eq. S29) consists of a constant *A.* A well-known value of *A* is 3.1e–6. However, for better accuracy, Julian derived a formula for the constant* A* (shown in Eq. (S34)). The* A* values listed in the fifth column of Table 3 result in slightly different values of Slack’s thermal conductivity (*κ*_*A*_) shown in the sixth column of Table 3 for all solid solutions models.

At low temperatures, the electron–phonon scattering is small. Thus, thermal conductivity *κ* is mainly contributed by lattice thermal conductivity *κ*_*L*_. *κ*_*L*_ has a relatively small values at low temperatures. *κ*_*L*_ has contributions from acoustic phonons (*κ*_*a*_) and optical phonons (*κ*_*o*_). It is crucial to calculate *κ*_*L*_ to ascertain whether the HE chalcogenides considered are good TE materials or not. We have calculated *κ*_*L*_, *κ*_*a*_, and *κ*_*o*_ using the Eqs. (S35), (S36) and (S37) respectively. Table 4 lists their values at 300 K. Figure 1f shows that m2, m5, and m8 have the lower *κ*_*L*_ among all other HE solid solutions. The Callaway-Debye theoretical model^118^ states that a higher *Θ*_*D*_ indicates a higher thermal conductivity and vice versa. We plotted *Θ*_*D*_ versus *κ* in Fig. S19a–d in SI to reveal the correlation between *Θ*_*D*_ and *κ*. Figure S19 shows that *Θ*_*D*_ is positively correlated with *κ* in agreement with Callaway-Debye model. The comparison between our calculated *κ* and *κ*_L_ with the experimental lattice thermal conductivity (*κ*_*L*_)_exp_ from Jiang’s work^66^ is listed in Table S10 showing similar trends. Melting temperature (*T*_melt_) is correlated to *Θ*_*D*_, *κ*_L_, and thermal expansion. Materials with low *T*_melt_ tend to have lower *Θ*_*D*_ and higher thermal expansion. For high temperature applications such as thermoelectric applications, it is crucial to identify the thermal limits or melting temperature (*T*_melt_) of a material. More information about the formula used to calculate* T*_melt_ can be found in (S38). The calculated *T*_melt_ for m0 and nine solid solutions are presented in Table S10. m1, m5, and m8 have the lowest *T*_melt_. Thermal expansion is the tendency of material to change the shape, volume, and density in response to a change in temperature. Thermal expansion is designated by thermal expansion coefficient (*α*). *α* for m0 and nine solid solutions can be estimated from the formula (S39). Table S10 shows that m1 and m5 have the largest α. The lattice vibrations in materials have a huge contribution to several physical properties, such as electrical conductivity, thermo-power, and thermal conductivity. The wavelength at which the heat phonon spectra or the phonon energy distribution curve strikes its maximum value is called the dominant phonon wavelength (*λ*_*dom*_).

**Table 4:** Lattice thermal conductivities *κ*_*L*_ (W⋅m^–1^. K^–1^) at 300 K calculated by using mixed model, *κ*_L_ contributed by acoustic phonons (*κ*_a_), and *κ*_L_ contributed by optical phonons (*κ*_o_) for ten models in Ge-Te based high-entropy chalcogenides.

Model	Mixed model *κ*_L_ (W.m^–1^.K^–1^)	Mixed model *κ*_a_ (W.m^–1^.K^–1^)	Mixed model *κ*_o_ (W.m^–1^.K^–1^)
m0	1.6750	1.5899	0.0851
m1	0.5743	0.5083	0.0660
m2	0.5457	0.4800	0.0658
m3	0.9048	0.8316	0.0732
m4	0.9708	0.8967	0.0741
m5	0.5740	0.5178	0.0562
m6	0.9290	0.8558	0.0732
m7	0.8963	0.8239	0.0725
m8	0.6142	0.5482	0.0661
m9	0.9696	0.8956	0.0740

Dominant phonon wavelength carries the majority of heat in most materials. It is important to calculate *λ*_*dom*_ to identify the total energy of phonons or the maximum energy of phonons at a certain temperature. *λ*_*dom*_ and the mean free path (MFP) (the average distance that a phonon travels between two successive inelastic collisions) are positively correlated. These two parameters play a significant role in controlling *κ*_L_. Shifting the heat phonon spectra towards shorter wavelengths (smaller *λ*_*dom*_) and shorter mean free paths may increase the scattering of phonons and reduce *κ*_L_^119^. *λ*_*dom*_ can be roughly estimated at 300 K by using the formula (S40). The calculated *λ*_*dom*_ for m0 and nine solid solutions are represented in Table S10. *λ*_*dom*_ has been shortened from 1.02 Å for m0 to 0.692 Å for m5 which indicates that the phonon heat spectra are strongly modified by HE alloying.

### PbSe-based high-entropy chalcogenides

#### Pb_0.99-y_Sb_0.012_Sn_y_Se_1-2x_Te_x_S_x_ (x = 0.1, 0.2, 0.25, 0.3, 0.35, 0.4, 0.45, with y = 0) solid solutions

**Figure 2:**
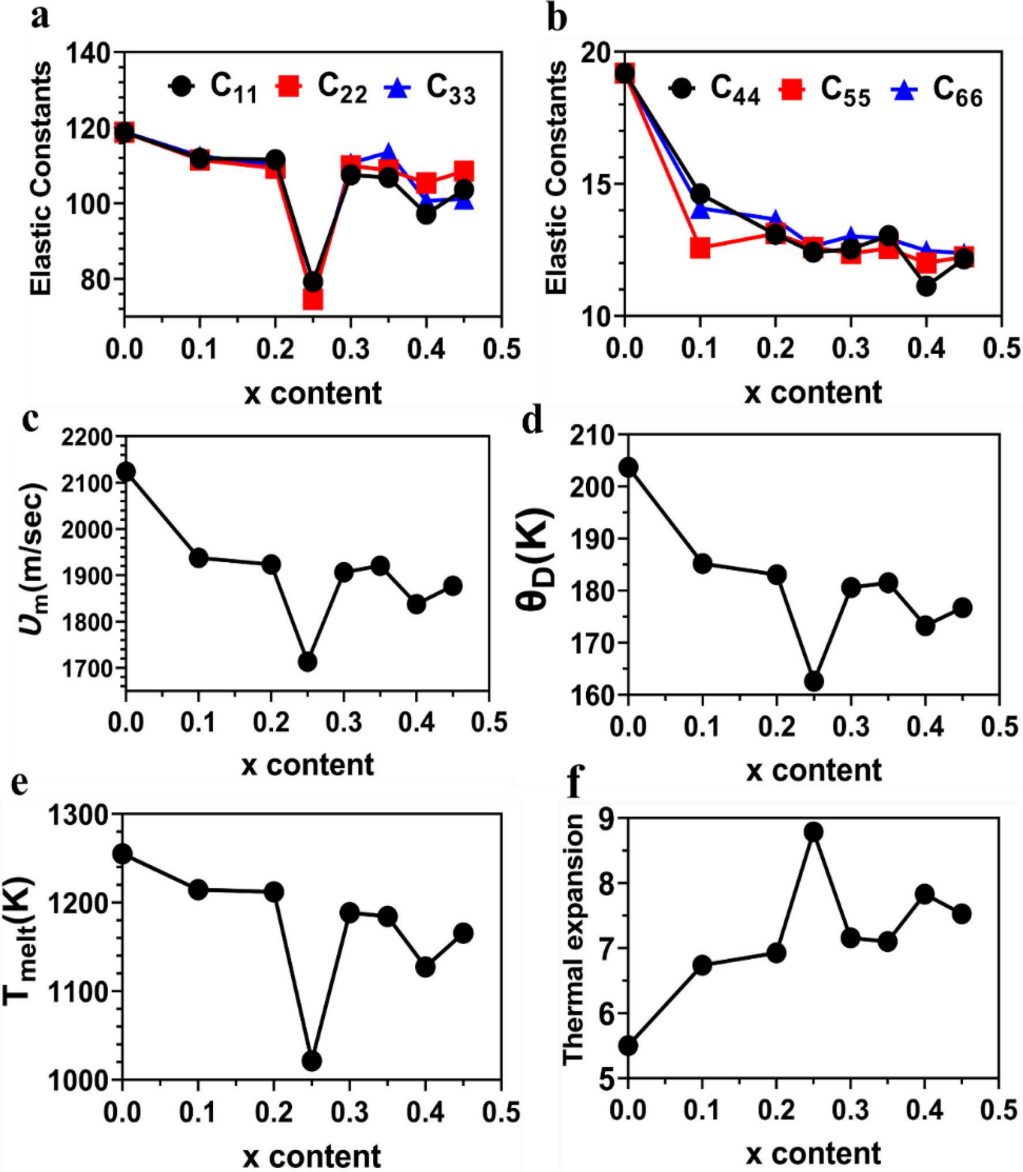
(**a**)The distribution of the elastic constants C_11_, C_22_, C_33_, (**b**) C_44_, C_55_, C_66_, (**c**) Average sound velocity, (**d**) Debye temperature, (**e**) melting temperature, and (**f**) thermal expansion of Pb_0.99-y_Sb_0.012_Sn_y_Se_1-2x_Te_x_S_x_ solid solution models with respect of x content (x = 0.1, 0.2, 0.25, 0.3, 0.35, 0.4, 0.45) and y = 0.

In this section, we focus on results of solid solutions Pb_0.99-y_Sb_0.012_Sn_y_Se_1-2x_Te_x_S_x_ (x = 0.1, 0.2, 0.25, 0.3, 0.35, 0.4, 0.45, and y = 0). The calculated elastic constants of pure PbSe and eight solid solutions are listed in Table S11. Some of these results are also shown in Fig. 2a and b. Figure 2a shows a sharp reduction of C_11_, C_22_, and C_33_ of Pb_0.99-y_Sb_0.012_Sn_y_Se_1-2x_Te_x_S_x_ (with y = 0 and x = 0.25) or Pb_0.99_Sb_0.012_Se_0.5_Te_0.25_S_0.25._ With increase of x content, a gradual reduction of C_44_, C_55_, and C_66_ is observed in Fig. 2b. The reduction of elastic constants with increase of x content implies an increase of the elastic strains. PbSe-based HE solid solution models have a cubic structure. Thus, a different empirical formula shown in Eq. (S41) was used to estimate the melting temperature (*T*_*melt*_) of both Pb_0.99-y_Sb_0.012_Sn_y_Se_1-2x_Te_x_S_x_(x = 0.1, 0.2, 0.25, 0.3, 0.35, 0.4, 0.45, and y = 0) and Pb_0.99-y_Sb_0.012_Sn_y_Se_1-2x_Te_x_S_x_(y = 0.00, 0.05, 0.10, 0.15, 0.20, 0.25, and x = 0.25). The theoretical density (*ρ*), the calculated sound velocity (longitudinal *ν*_*l*_, transverse *ν*_*s*_, and average *ν*_*m*_), *Θ*_D_, *T*_*melt*_, *α*, and *λ*_*dom*_ for Pb_0.99-y_Sb_0.012_Sn_y_Se_1-2x_Te_x_S_x_ ((x = 0.1, 0.2, 0.25, 0.3, 0.35, 0.4, 0.45) and y = 0) solid solutions are listed in Table S12. In general, *λ*_*dom*_ has been shortened for all models. *λ*_*dom*_ has been shortened from its highest value(0.890 Å) in pure PbSe to its lowest value(0.718 Å) for Pb_0.99_Sb_0.012_Se_0.5_Te_0.25_S_0.25_ model. Shortening *λ*_*dom*_ reduces the mean free path of phonons which increases scattering of phonons resulting in a reduced *κ*_L_.

Figure 2c–f depict the calculated *ν*_*m*_, *Θ*_*D*_. *T*_*melt*_, and *α* versus x content of Pb_0.99-y_Sb_0.012_Sn_y_Se_1-2x_Te_x_S_x_. *ν*_*m*_, *Θ*_*D*_, and *T*_*melt*_ decreases with increase of x content. The same trend is noticed for elastic constants. While thermal expansion increases with increase of x content, a sharp reduction in *ν*_*m*_, *Θ*_*D*_, and *T*_*melt*_ is notable for Pb_0.99-y_Sb_0.012_Sn_y_Se_1-2x_Te_x_S_x_ (with y = 0 and x = 0.25) or Pb_0.99_Sb_0.012_Se_0.5_Te_0.25_S_0.25_ (the same trend is common with elastic constants C_11_, C_22_, and C_33_). A sharp increase of *α* is noted for Pb_0.99_Sb_0.012_Se_0.5_Te_0.25_S_0.25_. Lower *ν*_*m*_ and *Θ*_*D*_ indicates weaker chemical bonds and a lower *κ*_*L*_. The reduction in *ν*_*m*_ with increase of x content is large in the current study (see Fig. 2c). A small decrease in *ν*_*m*_ corresponds to large decrease in *κ*_*L*_. The large reduction in *Θ*_*D*_ with increase of x content (see Fig. 2d) indicates weaker chemical bonds which results in higher anharmonicity or higher Grüneisen parameter, thus higher phonon scattering and lower *κ*_*L*_. Clearly, increasing x content results in higher strains or lower elastic constants. Thus, the sound velocities or phonon velocities (longitudinal (*ν*_*l*_) and transverse (*ν*_*s*_)) decrease.

**Figure 3:**
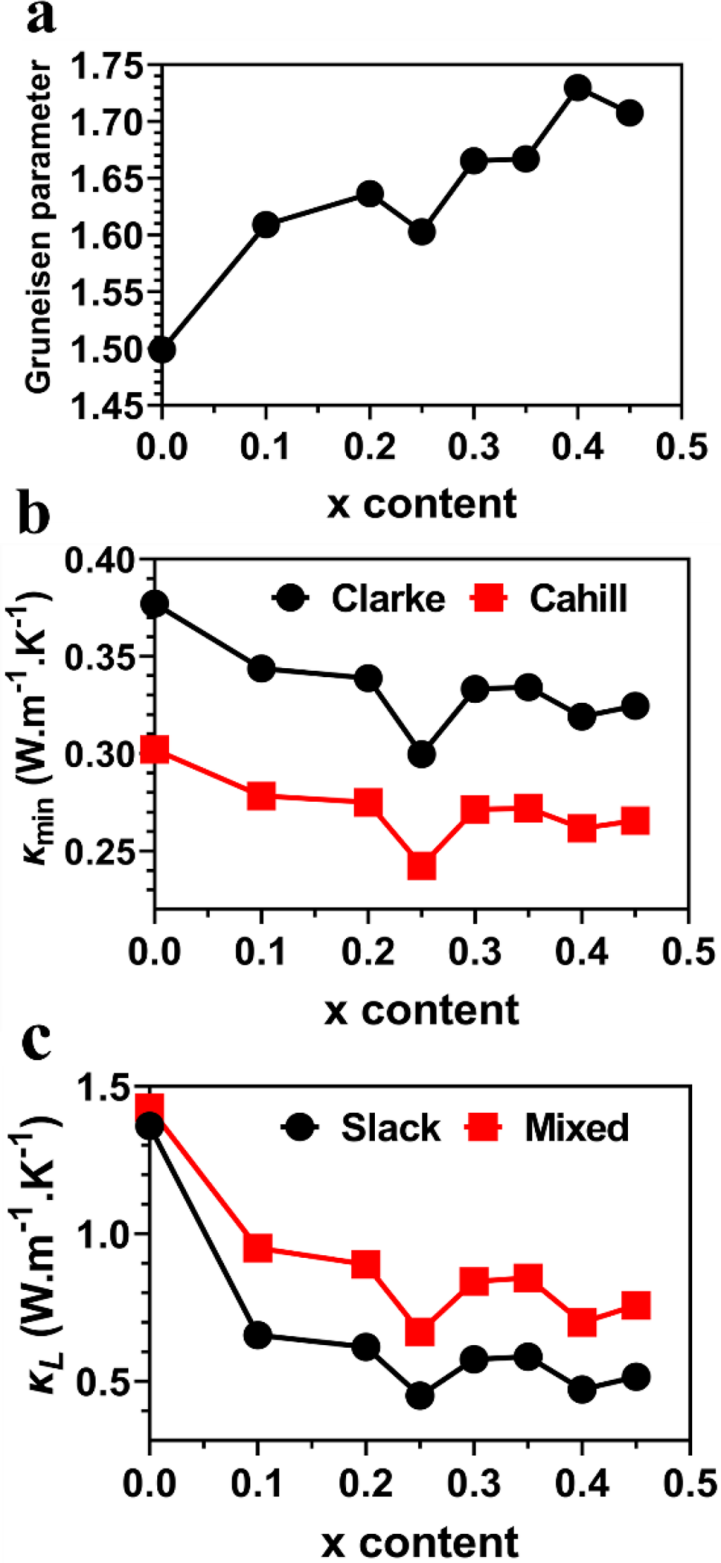
(**a**) Acoustic Grüneisen constant, (**b**) Minimum thermal conductivities, and (**c**) Lattice thermal conductivities at 300 K for Pb_0.99-y_Sb_0.012_Sn_y_Se_1-2x_Te_x_S_x_ solid solution models with respect to x content with y = 0.

This can be understood via the correlation between the phonon velocity and the elastic constants shown in Eqs. (2) and (3). Reducing the sound or phonon velocity via increasing the strains is one of the engineering methods for reducing *κ*_*L*_ and consequently enhancing the TE performance. Phonon velocities and *κ*_*L*_ are correlated by the Eq. (4)^120^:

4 $$\begin{array}{c}{\kappa }_{ij}=\sum_{\alpha }{C}_{\alpha }{\tau }_{\alpha }{v}_{i}{v}_{j}, \end{array}$$

where ${C}_{\alpha }, {\tau }_{\alpha },$ and $\upsilon$ are the heat capacity, phonon scattering time or relaxation time, and phonon velocity, respectively. $i, j$ refers to the three principal axes of the chosen coordinate system. The element substitution creates a disorder by modify the atomic positions. Thus, weak displacements of the atoms and bonds were produced, resulting in enhancement of bond anharmonicity which results in higher *γ*_*α*_. Figure 3a shows the Grüneisen parameter increases with increase of x content of Pb_0.99-y_Sb_0.012_Sn_y_Se_1-2x_Te_x_S_x_, which results in a reduced *κ*_*L*_ (see Fig. 3c). Figure 3b and c show *κ*_*min*_ and *κ*_*L*_ are reduced with increase of x content. This is an evidence of elastic strain’s effect on thermal conductivity. The calculated thermal conductivities and Grüneisen parameter of Pb_0.99-y_Sb_0.012_Sn_y_Se_1-2x_Te_x_S_x_ models are listed in Table 5.

**Table 5:** Calculated thermal (*κ*) and minimum thermal (*κ*_min_) conductivities (W⋅m^–1^. K^–1^) at 300 K, lattice thermal conductivities *κ*_*L*_ (W⋅m^–1^. K^–1^) at 300 K, *κ*_L_ contributed by acoustic phonons (*κ*_a_), and *κ*_L_ contributed by optical phonons (*κ*_o_), and Grüneisen parameter (*γ*_a_) for pure PbSe and Pb_0.99-y_Sb_0.012_Sn_y_Se_1-2x_Te_x_S_x_ solid solutions.

x	Clarke model *κ*_min_ (W.m^–1^.K^–1^)	Cahill model *κ*_min_ (W.m^–1^.K^–1^)	Slack model *κ* (W.m^–1^.K^–1^)	Mixed model *κ*_L_ (W.m^–1^.K^–1^)	*κ*_a_(W.m^–1^.K^–1^)	*κ*_o_ (W.m^–1^.K^–1^)	*γ*_a_
–	0.3772	0.3019	1.3656	1.4273	1.3484	0.0789	1.499
0.10	0.3437	0.2781	0.6566	0.9510	0.8795	0.0715	1.609
0.20	0.3388	0.2749	0.6164	0.8961	0.8258	0.0703	1.636
0.25	0.2996	0.2423	0.4517	0.6675	0.6051	0.0623	1.603
0.30	0.3331	0.2712	0.5744	0.8385	0.7694	0.0691	1.665
0.35	0.3340	0.2720	0.5835	0.8509	0.7816	0.0693	1.667
0.40	0.3189	0.2617	0.4726	0.6991	0.6331	0.0659	1.730
0.45	0.3245	0.2655	0.5156	0.7578	0.6907	0.0672	1.708

The calculated Young’s modulus (E), bulk modulus (K), shear modulus (G), Poisson’s ratio (η), Pugh’s ratio (G/K), Vicker’s hardness (*H*_*V*_), Kleinman parameter(ζ), machinability index(*µ*_*M*_), Cauchy pressures (CP), and Lame’s constants (*λ*, *µ*) for Pb_0.99-y_Sb_0.012_Sn_y_Se_1-2x_Te_x_S_x_ solid solutions (x = 0.1, 0.2, 0.25, 0.3, 0.35, 0.4, 0.45) and y = 0 are listed in Table S13. Grüneisen parameter versus *v*_*l*_ /*v*_*s*_ plot is shown in Fig. S20 which has a positive correlation similar to GeTe and its solid solutions. Figure S21a–d shows the distribution of the E, K, G, and η of Pb_0.99-y_Sb_0.012_Sn_y_Se_1-2x_Te_x_S_x_ versus x content. As can be seen, there is a gradual reduction of E, K, and G for all solid solution models with increase of x content with a notable sharp reduction with y = 0 and x = 0.25. On the other hand, Poisson’s ration increases with increase of x content. A gradual increase of the machinability index for all solid solution models with increase of x content, with a notable dip at 0.25 × content in Fig. S22a. In Fig. S22b, the first Lame’s constant (*λ*) is larger than the second Lame’s constant (*μ*) for all solid solutions models indicating a higher compressibility than shear.

Pugh’s ratio (G/K) is also listed in Table S13. All solid solution models tend to be ductile, except pure PbSe which tends to be brittle. Cauchy pressure (CP) is calculated by using the formula: (C_12_–C_44_)^121^. The calculated CP of Pb_0.99-y_Sb_0.012_Sn_y_Se_1-2x_Te_x_S_x_ versus x (x = 0.1, 0.2, 0.25, 0.3, 0.35, 0.4, 0.45) and y = 0 are listed in Table S13. Large positive CP value is noted for Pb_0.99-y_Sb_0.012_Sn_y_Se_1-2x_Te_x_S_x_ (x = 0.25, y = 0) or Pb_0.99_Sb_0.012_Se_0.5_Te_0.25_S_0.25_ which indicates dominant ionic bonds. The calculated* A*^*U*^, *G*_*V*_, *G*_*R*_, *K*_*V*_, and *V*_*R*_ are given in Table S14. *A*^*U*^ of Pb_0.99-y_Sb_0.012_Sn_y_Se_1-2x_Te_x_S_x_ varies from unity in different degrees. However, Pb_0.99-y_Sb_0.012_Sn_y_Se_1-2x_Te_x_S_x_ (x = 0.25, y = 0) or Pb_0.99_Sb_0.012_Se_0.5_Te_0.25_S_0.25_ shows isotropic nature compared to other solid solutions.

#### Pb_0.99-y_Sb_0.012_Sn_y_Se_1-2x_Te_x_S_x_ (y = 0.0, 0.05, 0.1, 0.15, 0.2, 0.25, and x = 0.25) solid solutions

In this section, we focus on results of solid solutions Pb_0.99-y_Sb_0.012_Sn_y_Se_1-2x_Te_x_S_x_ (y = 0.0, 0.05, 0.1, 0.15, 0.2, 0.25, and x = 0.25). The calculated elastic constants, Young’s modulus, bulk and shear moduli, Poisson’s ration, Vicker’s hardness, Kleinman parameter, machinability index, Cauchy pressure, Lame’s constants, and the universal anisotropic index of Pb_0.99-y_Sb_0.012_Sn_y_Se_1-2x_Te_x_S_x_ (y = 0.0, 0.05, 0.1, 0.15, 0.2, 0.25, and x = 0.25) solid solutions are listed in Tables S16, S17, S18 and S19. Figure S23a to d show the calculated *ν*_*m*_, *Θ*_*D*_, *T*_*melt*_, and *α* versus y content of Pb_0.99-y_Sb_0.012_Sn_y_Se_1-2x_Te_x_S_x_(y = 0.0, 0.05, 0.1, 0.15, 0.2, 0.25) and x = 0.25. The first model: Pb_0.99-y_Sb_0.012_Sn_y_Se_1-2x_Te_x_S_x_ (y = 0.0, x = 0.25) is discussed in the previous section. It is noticed that *ν*_*m*_ and *Θ*_*D*_ have a sharp peak at y = 0.05 and then a gradual decrease with the increase in y content. A sharp reduction in *T*_melt_ is notable for Pb_0.99-y_Sb_0.012_Sn_y_Se_1-2x_Te_x_S_x_ (with y = 0.1 and x = 0.25). Thermal expansion coefficient has a sharp decrease at y = 0.05 and y = 0.1 and then gradually increases. Clearly, increasing y content while keeping x = 0.25 results in lower *ν*_*m*_, *Θ*_*D*_, and *T*_*melt*_. The calculated thermal conductivities and Grüneisen parameter of Pb_0.99-y_Sb_0.012_Sn_y_Se_1-2x_Te_x_S_x_ ((y = 0.0, 0.05, 0.1, 0.15, 0.2, 0.25) with x = 0.25) are listed in Table S20. The calculated density (*ρ*), *ν*_*l*_, *ν*_*s*_, *ν*_*m*_, *Θ*_*D*_, *T*_*melt*_, *α*, and *λ*_*dom*_(Å) for Pb_0.99-y_Sb_0.012_Sn_y_Se_1-2x_Te_x_S_x_ ((y = 0.0, 0.05, 0.1, 0.15, 0.2, 0.25) and x = 0.25) are listed in Table S21. In Fig. S24a, we notice the Grüneisen parameter of Pb_0.99-y_Sb_0.012_Sn_y_Se_1-2x_Te_x_S_x_ has a notable dip at 0.1 y-content and then increasing rapidly with increase of y content. In Fig. S24b, *κ*_*min*_ starts to increase till 0.05 of y-content and then gradually decreases thereafter. In Fig. S25, *κ*_*L*_ starts to increase till 0.1 y-content and then decreases thereafter. y-content above 0.1 in Pb_0.99-y_Sb_0.012_Sn_y_Se_1-2x_Te_x_S_x_ (x = 0.25) models results in much higher Grüneisen parameter and consequently, a reduced *κ*_*L*_.

## Discussion

In GeTe-based HE models, Alloying by Pb and Bi atoms at the Ge atom sites produced higher mass and strain field fluctuations to scatter phonons due to the larger atomic size and heavier mass of Bi and Pb than Ge. This may be the main reason for the lower *κ*_*L*_ in most nine HE models under study. Furthermore, the alloy defects may have strengthened the phonon scattering, resulting in much depressed *κ*_*L*_. Our results for *κ*_*L*_ show the same trend as the experimental data of Jiang et al.^66^. Materials with low *Θ*_*D*_ and low thermal conductivity have remarkable applications as TE and thermal barrier coatings (TBC) materials. We revealed that there is a positive correlation between Debye temperature and thermal conductivity for the investigated nine HE models. A strong anharmonicity (high Grüneisen parameter) indicates weak atomic bonds, and thus low *κ*_*L*_^122, 123^. There is a positive correlation between sound velocity and the strength of interatomic interactions. Lower sound velocity indicates weaker interatomic interactions between atoms as indicated by large Grüneisen parameter^124^. We know from Eq. (4) that *κ*_*L*_ can be reduced by minimizing three parameters: heat capacity (C_α_), phonon relaxation time (τ_α_), and phonon velocity (υ). If the speed of sound (i.e. lattice stiffness) can be manipulated via some methods, *κ*_*L*_ can be reduced significantly. One of the techniques for reducing speed of sound is inducing internal-strain fields, which are induced by lattice defects such as dislocations, alloying, and doping^125^. Internal- strains change the speed of sound which results in higher phonon scattering and lower *κ*_*L*_. At small strains, the phonon frequency (ω) and the speed of sound, the Grüneisen tensor (*γ*_*ij*_), and the strain tensor (*ε*_*ij*_) are correlated by the following formula^126^:

5 $$\begin{array}{c}\omega ={\omega }_{0}\left(1-{\gamma }_{ij}{\varepsilon }_{ij}\right), \end{array}$$

where ${\omega }_{0}$ is the phonon frequency at zero strain. Clearly, increasing $\gamma$ and *ε* will reduce the phonon frequency and thus reduce *κ*_*L*_. Compared to m0 (pure GeTe), the lattice parameters have been increased in m5 (Ge_0.61_Ag_0.11_Sb_0.13_Pb_0.12_Bi_0.01_Te) due to the alloying (see Table S2), which indicates an increase in atomic separation distances. This increase in atomic separation distances weakens the chemical bond strength between atoms, thereby elastic constants significantly decreased in m5 (see Table S5). m5 has been softened (much lower Young’s modulus, bulk and shear moduli) due to the weak bond strength (see Table 1). Softening the chemical bonds also decreases phonon group velocity (see Table 2 for m5 model). The dramatic change of the values of elastic properties in m5 indicates that the deformation resistance of this model has a significant change. The current DFT calculations cannot fully explain the reason behind the increase of elastic properties when alloying with a small fraction of element Cu in m6 (Ge_0.61_Ag_0.11_Sb_0.13_Pb_0.12_Bi_0.01_Cu_0.003_Te), subsequent to their suppression in m5. Multiple elements alloying introduces atomic disorder (either substitutionally or interstitially) in the HE model lattice, which in general is considered as point defects. Alloying also causes severe lattice distortion which together with the point defects are favorable for dislocation formation^127^. This results in a strong strain field to reduce lattice thermal conductivity. Dislocations can significantly change the mechanical properties. More dislocations in the lattice results in much lower Young’s modulus, bulk and shear moduli. However, Cu substitutional alloying in m6 may suppress the dislocation formation, resulting in higher elastic properties than m5.

In PbSe-based HE models, both strains and Grüneisen parameter have been increased through alloying by increasing x content of Pb_0.99-y_Sb_0.012_Sn_y_Se_1-2x_Te_x_S_x_. Rafal et al.^116^ has shown that enhancing bond anharmonicity by alloying of Pb_1-x_Sn_x_Te results in larger *γ*_*α*_ which in return results in a reduced *κ*_*L*_. We conclude that alloying with Sb, Sn, Te, and S atoms caused an increase of elastic strains and *γ*_*α*_, a reduction of phonon velocity, Debye temperature, and consequently a significant reduction in* κ*_*L*_. There is a dramatic decrease in the values of elastic constants (C_11_, C_22_, C_33_) as x content increases to 0.25 (see Fig. 2a), indicating a significant change in the deformation resistance of the Pb_0.99_Sb_0.012_Se_0.5_Te_0.25_S_0.25_ model. This dramatic decrease (at x = 0.25) in the values of elastic constants causes a dramatic decline of *ν*_*s*_, *ν*_*m*_, and *θ*_*D*_ of Pb_0.99_Sb_0.012_Se_0.5_Te_0.25_S_0.25_ (see Fig. 2c and d). Elastic moduli exhibit a decline as the x content increases, and the identical trend is observed for mechanical properties–Young’s, bulk, and shear moduli. However, there is dramatic decrease in mechanical properties at the x content of 0.25 (see Fig.S21a–c). This indicates a dramatic decline of the deformation resistance of this HE solid solution (Pb_0.99_Sb_0.012_Se_0.5_Te_0.25_S_0.25_). At x = 0.25, *κ* and *κ*_*L*_ have also the smallest values (see Fig. 3b and c), indicating larger lattice distortion, which can strengthen phonon scattering due to mass and strain field fluctuations in matrix lattice. Lattice distortion introduced by Te and S alloying in PbSe is favorable for dislocation formation. Higher dislocation density indicates very small *κ*_*L*_. Our calculations show that the denser dislocation may occur at x = 0.25 (Pb_0.99_Sb_0.012_Se_0.5_Te_0.25_S_0.25_). After x = 0.25, the dislocation density starts decreasing and causing higher *κ*_*L*_ and elastic properties.

## Conclusion

The elastic and thermal properties of pure GeTe(m0) and nine high-entropy models: Ge_0.77_Ag_0.11_Pb_0.12_Te(m1),Ge_0.75_Sb_0.13_Pb_0.12_Te(m2),Ge_0.74_Ag_0.11_Sb_0.13_Te(m3), Ge_0.62_Ag_0.11_Sb_0.13_Pb_0.12_Te(m4),Ge_0.61_Ag_0.11_Sb_0.13_Pb_0.12_Bi_0.01_Te(m5), Ge_0.61_Ag_0.11_Sb_0.13_Pb_0.12_Bi_0.01_Cu_0.003_Te(m6),Ge_0.61_Ag_0.11_Sb_0.13_Pb_0.12_Cd_0.05_Bi_0.01_Te(m7), Ge_0.61_Ag_0.11_Sb_0.13_Pb_0.12_Mn_0.05_Bi_0.01_ T(m8), and Ge_0.61_Ag_0.11_Sb_0.13_Pb_0.12_Sn_0.05_Bi_0.01_Te(m9) were investigated using first-principles calculations. The alloying of these solid solutions was carried out using the random solid solution model (RSSM) which is efficient for large supercells of 1080 atoms. Based on our calculations, m0 and all nine solid solution models are mechanically stable. m5 has the lowest Young’s, bulk and shear moduli. Generally speaking, random alloying of GeTe by Ag, Sb, Pb, Bi, Cu, Mn, Cd, and Sn elements at the Ge sites results in a reduced hardness, sound velocity, Debye temperature, and thermal conductivity. Among the nine solid solution models, m2, m5, m8 have the lowest Debye temperature and thermal conductivity. m1, m2, m5, and m8 have much lower lattice thermal conductivity compared to pure GeTe (m0). The models m2, m5, and m8 have the lowest and comparable values of *κ*_*L*_ (see Fig. 1f). Mechanical and thermal properties of thirteen PbSe-based high-entropy chalcogenide models: Pb_0.99-y_Sb_0.012_Sn_y_Se_1-2x_Te_x_S_x_ (x = 0.1, 0.2, 0.25, 0.3, 0.35, 0.4, 0.45, and y = 0) and Pb_0.99-y_Sb_0.012_Sn_y_Se_1-2x_Te_x_S_x_ (y = 0.05, 0.1, 0.15, 0.2, 0.25 and x = 0.25) are investigated in detail. The reduction in speed of sound and lattice thermal conductivity correspond to an increase in internal elastic strains. We conclude that alloying with Sb, Sn, Te, and S atoms caused an increase of elastic strains and *γ*_*α*_, a reduction of phonon velocity, Debye temperature, and consequently a significant reduction in* κ*_*L*_. Despite a large number of complex calculations on mechanical and thermal properties of very large supercells of high-entropy chalcogenides were done for the first time, there are obviously some drawbacks such as ignoring the lattice dynamics(phonon calculations), However, phonon calculations is almost impossible to be carried out for such large supercells. We are encouraged by the current results and aspire to continue research in this direction for more complex and interesting high-entropy chalcogenides. It is desirable to improve the DFT calculations with better options, such as using either hybrid potential or Becke–Johnson potential. Finally, we believe our results can facilitate the design of new high-entropy chalcogenides with better thermoelectric performance.

## Materials and methods

### Density functional theory method

Density functional theory (DFT) based method is utilized to perform the calculations implemented in the *Vienna *Ab initio Simulation Package (VASP)^128^. VASP is used to optimize the structures of solid solution models and calculate the mechanical properties. The one-electron orbitals are expanded in the plane wave basis set with an energy cut-off of 600 eV. The generalized gradient approximation (GGA) of Perdew, Burke, and Ernzerhof (PBE)^129^ is used as the exchange and correlation potential for solving the Kohn–Sham equation. The electronic and ionic force convergence criteria are set at 10^−6^ eV and 10^−5^ eV/Å respectively. The Monkhorst scheme^130^ is used with k-point meshes of 2 × 2 × 1 for GeTe-based HE solid solutions, while k-point meshes of 1 × 1 × 1 were used for all PbSe-based HE solid solutions. The stress vs strain scheme is used to calculate the elastic tensor. The following linear equation, also called the Hooke’s law, can be solved to obtain the elastic coefficient, *C*_*ij*_:

6 $$\begin{array}{c}{\sigma }_{i}=\sum_{j=1}{C}_{ij}{\epsilon }_{j}, \end{array}$$

where $i$ and $j$ range from 1 to 6. The *σ*_*i*_ is obtained by applying a strain *є*_*j*_ of + 0.50% and − 0.50% to the equilibrium structure. More details on mechanical properties calculation are described in SI.

### Supercell construction

GeTe-based supercells for high-entropy chalcogenide models in trigonal lattice are constructed based on the random solid solution model (RSSM). In RSSM method, the determination of the number of atoms, N, in the supercell is crucial. *N* in all ten models is calculated by the simple formula: *N* = 6 × (*n*^2^ × *m*), where the number 6 is the number of atoms in the GeTe simple cell. *n* and *m* are set to be 6 and 5, respectively, so *N* is 1080 atoms in the trigonal supercell. The lengths of the supercells equal to *n* × *a* and *m* × *c*, where *a* and *c* are the lattice constants of the simple trigonal GeTe crystal. RSSM method requires a large supercell with large number of atoms. A 1080-atom supercell can be considered to be the minimal size to justify the use of RSSM with high confidence. It is necessary to use “supercell” to capture different possible structural configurations of the HE models. In the present study, we assure that the statistical distribution of random distribution of alloying elements (Ag, Sb, Pb, Bi, Cu, Cd, Mn, and Sn) is sufficient due to sufficiently large supercell with periodical boundary conditions that can account for the random distribution of the NN, second NN, and the third NN for each atom in the model. In compliance with the spirit of RSSM, it is carried out by writing a small script such that the atomic occupation of each site is completely random with no restriction to their NN atoms and beyond^131^. For PbSe-based HE models, the same procedure was followed, except that the formula of *N* was set to be: *N* = 8 × (*n*^3^), where the number 8 is the number of atoms in the PbSe simple cell and *n* is set to be 5. The lengths of the supercells equal to *n* × *a* where *a* is the lattice constant of the simple cubic PbSe crystal.

### Supplementary Information

Supplementary Information.

## Data Availability

All the data in this paper including those in the supplementary information materials are freely available by contacting one of the corresponding authors (chingw@umkc.edu).
