# Supplementary material for: Ab initio study of mechanical and thermal properties of GeTe-based and PbSe-based high-entropy chalcogenides
Source: Sci Rep. 2023 Sep 27;13:16218. doi: 10.1038/s41598-023-42101-5 (PMC10533554; doi:10.1038/s41598-023-42101-5)
Supplement: Supplementary file 1 — Supplementary Information. [file 41598_2023_42101_MOESM1_ESM.pdf]

Supplementary Information for

Ab initio study of mechanical and thermal properties of GeTe-based and PbSe-based high-entropy chalcogenides

Sahib Hasan[1,2], Puja Adhikari[1], Saro San[1] and Wai-Yim Ching[1,*]

1. Department of Physics and Astronomy, University of Missouri-Kansas City, Kansas City, MO 64110, USA
2. Department of Sciences, College of Basic Education, Al Muthanna University, Samawah 66001, Iraq

***** Correspondence: chingw@umkc.edu

This PDF file includes:

Materials and Methods
Supplementary Text
Figs. S1 to S29
Tables S1 to S21
References (1 to 36)

Materials and Methods

Mechanical properties calculations

Ab initio calculation method within the density functional theory (DFT) is a very powerful method these days for mechanical properties calculations. In this section, we followed the method of Hongzhi Yao *et al*. work[1] to calculate the mechanical properties. A scheme has been applied to calculate the elastic constants of the high entropy solid solutions. Starting with the simple Hook's famous law[2,3] that relates the stress components σ_i with the strain components ε_j by the relation:

$$\sigma_i = \sum_{j=1}^{6} C_{ij}\, \varepsilon_j \qquad (S1)$$

C_{ij} is the elastic constants. From knowing the elastic constants, we calculate the other mechanical properties: compliance tensor S_{ij}, Young's modulus (E), Bulk modulus (K), Shear modulus (G), and Poisson's ratio (η). Here, the Voigt – Reuss –Hill (VRH)[4,5] approximation has been used to derive the above mechanical parameters. According to this approximation, the upper and lower bounds for the structural parameters, such as bulk and shear modulus are respectively given by:

$$K_V = \frac{(C_{11} + C_{22} + C_{33})}{9} + \frac{2(C_{12} + C_{13} + C_{23})}{9} \qquad (S2)$$

$$K_R = \frac{1}{(S_{11}+S_{22}+S_{33})+2(S_{12}+S_{13}+S_{23})} \qquad (S3)$$

$$G_V = \frac{(C_{11} + C_{22} + C_{33} - C_{12} - C_{13} - C_{23})}{15} + \frac{(C_{44} + C_{55} + C_{66})}{5} \qquad (S4)$$

$$G_R = \frac{15}{4(S_{11}+S_{22}+S_{33})-4(S_{12}+S_{13}+S_{23})+3(S_{44}+S_{55}+S_{66})} \qquad (S5)$$

So, the average values of the mechanical parameters are given by:

$$K = \frac{(K_V + K_R)}{2} \qquad (S6)$$

$$G = \frac{(G_V + G_R)}{2} \qquad (S7)$$

$$E = \frac{9KG}{(3K+G)} \qquad (S8)$$

$$\eta = \frac{(3K - 2G)}{2(3K + G)} \qquad (S9)$$

The dimensionless Kleinman parameter (ζ) is calculated by using the following formula[6]:

$$\zeta = \frac{C_{11} + 8C_{12}}{7C_{11} + 2C_{12}} \qquad (S10)$$

The machinability index (μ_M) can be expressed as follows [7]:

$$\mu_M = \frac{K}{C_{44}} \qquad (S11)$$

The formula of Tian *et al.* [8] was used to calculate the macro Vicker's hardness parameter H_V:

$$H_V = 0.92\left(\frac{G}{K}\right)^{1.137} G^{0.708} \qquad (S12)$$

Another semi-empirical formula [9] was used to estimate the micro-hardness (H):

$$H = \frac{E(1 - 2\eta)}{6(1 + \eta)} \qquad (S13)$$

Lame's constants (λ, μ) are derived from Young's modulus and Poisson's ratio as following [10]:

$$\lambda = \frac{\eta E}{(1 + \eta)(1 - 2\eta)} \qquad (S14)$$

$$\mu = \frac{E}{2(1 + \eta)} \qquad (S15)$$

The elastic anisotropy parameter is characterized by the universal anisotropic index A^U [11]:

$$A^U = 5\frac{G_V}{G_R} + \frac{K_V}{K_R} - 6 \qquad (S16)$$

Where the subscripts in G_V, G_R, K_V, and V_R are the shear and bulk modulus under Voigt and Reuss estimates respectively.

The percentage of anisotropy in compression and shear are given by[12]:

$$A_{comp} = \frac{K_V - K_R}{K_V + K_R} \qquad (S17)$$

$$A_{shear} = \frac{G_V - G_R}{G_V + G_R} \qquad (S18)$$

The shear anisotropic factor for the {100} shear planes between the [011] and [010] directions (A_1) is given by:

$$A_1 = \frac{4C_{44}}{C_{11} + C_{33} - 2C_{13}} \qquad (S19)$$

The shear anisotropic factor for the {010} shear planes between the [101] and [001] directions (A_2) is given by:

$$A_2 = \frac{4C_{55}}{C_{22} + C_{33} - 2C_{23}} \qquad (S20)$$

The shear anisotropic factor for the {001} shear planes between the [110] and [010] directions (A_3) is given by:

$$A_3 = \frac{4C_{66}}{C_{11} + C_{22} - 2C_{12}} \qquad (S21)$$

Debye temperature and thermal conductivity calculations

Debye temperature (Θ_D) and the average sound velocity (v_m) can be calculated as follows [13–16]:

$$\Theta_D = \frac{h}{k_B}\left[\frac{3n}{4\pi}\left(\frac{N_A.\rho}{M}\right)\right]^{\frac{1}{3}} v_m \qquad (S22)$$

$$v_m = \left[\frac{1}{3}\left(\frac{2}{v_s^3} + \frac{1}{v_l^3}\right)\right]^{-\frac{1}{3}} \qquad (S23)$$

Here, ρ is the theoretical density of the solid solution model, h, k_B, and N_A are Planck's constant, Boltzmann constant, and Avogadro's number, respectively. M is the molecular weight and n is the number of atoms in the supercell. v_s and v_l are the transverse (shear) and longitudinal sound velocities respectively. The compressional (longitudinal) waves and shear waves are estimated by using the values of bulk modulus (K) and shear modulus (G) according to Voigt-Reuss-Hill approach based on the following formulas [17]:

$$v_s = \sqrt{\frac{G}{\rho}} \qquad (S24)$$

$$v_l = \sqrt{\frac{3K + 4G}{3\rho}} \qquad (S25)$$

The acoustic impedance parameter (Z) value of m0 and nine solid solutions can be estimated by using the following formula [18,19]:

$$Z = \sqrt{\rho G} \tag{S26}$$

Clarke's formula for the minimum thermal conductivity ($\kappa_{\min}$) is given as follows[20,21]:

$$\kappa_{min} = 0.87 k_B \left(\frac{N_A . n . \rho}{M}\right)^{\frac{2}{3}} \sqrt{\frac{E}{\rho}} \tag{S27}$$

Cahill's formula for the minimum thermal conductivity is given by[22,23]:

$$\kappa_{min} = \frac{k_B}{2.48} \left(\frac{n}{V}\right)^{\frac{2}{3}} (\nu_l + 2\nu_s) \tag{S28}$$

Slack's formula[24,25] for thermal conductivity (κ) or for lattice thermal conductivity (κ_L) (in case of the electronic part of thermal conductivity (κ_e) is negligible) is given by:

$$\kappa = A \frac{M_{ave} {\Theta_D}^3 \Omega}{\left(\gamma_\alpha^2 . n^{\frac{2}{3}} . T\right)} \tag{S29}$$

$$M_{ave} = \frac{M}{n . N_A} \tag{S30}$$

Where V is the volume of the supercell, A is constant that can be approximated to be 3.1e-6[25] when κ in W.m^{-1}.K^{-1}. γ_α is the acoustic Grüneisen parameter, Ω is the volume per atom, and T is the temperature in Kelvin unit.

The acoustic Grüneisen constant (γ_α) was calculated by using the following formula [26]:

$$\gamma_\alpha = \frac{3}{2}\left(\frac{3\nu_l^2 - 4\nu_s^2}{\nu_l^2 + 2\nu_s^2}\right) \tag{S31}$$

Another formula that can be used to estimate the Grüneisen parameter [27,28] is:

$$\gamma_\alpha = \frac{3(1+\eta)}{2(2-3\eta)} \tag{S32}$$

Another formula that can be used to estimate the Poisson's ratio[29]

$$\eta = \frac{1 - 2(\frac{\nu_s}{\nu_l})^2}{2 - 2(\frac{\nu_s}{\nu_l})^2} \tag{S33}$$

Julian [24,27,30] derived the following formula for the constant A in the Slack's formula(formula (S29)) for κ:

$$A = \frac{2.436 \times 10^{-6}}{1 - \frac{0.514}{\gamma_\alpha} + \frac{0.228}{\gamma_\alpha^2}} \quad (S34)$$

The mixed model [31] can give an empirical formula for lattice thermal conductivity(κ_L) as follows:

$$\kappa_L = \frac{(6\pi^2)^{\frac{2}{3}}}{4\pi^2} . \frac{M_{ave} v_s^3}{V^{\frac{2}{3}} \gamma^2 T} \quad (S35)$$

For complex materials, κ_L mainly has two contributions: the acoustic (κ_a) and optical (κ_o) contributions separately, which are given by [31]:

$$\kappa_a = \frac{(6\pi^2)^{\frac{2}{3}}}{4\pi^2} . \frac{M_{ave} v_s^3}{V^{\frac{2}{3}} \gamma^2 T} \left(\frac{1}{n^{\frac{1}{3}}} \right) \quad (S36)$$

$$\kappa_o = \frac{3 k_B v_s}{2 V^{\frac{2}{3}}} \left(\frac{\pi}{6} \right)^{\frac{1}{3}} \left(1 - \frac{1}{n^{\frac{2}{3}}} \right) \quad (S37)$$

An empirical formula developed by Fine *et al.* [32] is used for calculating a rough estimation of melting temperature(T_{melt}) of m0 and nine solid solutions(GeTe-based high-entropy chalcogenide models) with a standard error about ±300 K by using the elastic constants:

$$T_{melt} = 354K + \left(\frac{4.5K}{GPa} \right) \left(\frac{2C_{11} + C_{33}}{3} \right) \mp 300K \quad (S38)$$

Thermal expansion coefficient (α) can be roughly estimated from the following formula using the value of shear modulus [33]:

$$\alpha = \frac{1.6x10^{-3}}{G} \quad (S39)$$

The maximum value of phonon wavelength (λ_{dom}) can be roughly estimated at any temperature by using the following formula [20]:

$$\lambda_{dom} = \frac{12.566 v_m}{T} x10^{-12} \quad (S40)$$

A different empirical formula was used for calculating an estimation of melting temperature(T_{melt}) of $Pb_{0.99-y}Sb_{0.012}Sn_ySe_{1-2x}Te_xS_x$(x=0.1, 0.2, 0.25, 0.3, 0.35, 0.4, 0.45, and y=0) and $Pb_{0.99-y}Sb_{0.012}Sn_ySe_{1-2x}Te_xS_x$(y=0.00, 0.05, 0.10, 0.15, 0.20, 0.25, and x= 0.25) solid solutions with a standard error about ±300 K by using the elastic constants[32,34,35]:

$$T_{melt} = \left[553K + \left(\frac{5.91K}{GPa}\right) C_{11}\right] \mp 300K \qquad (S41)$$

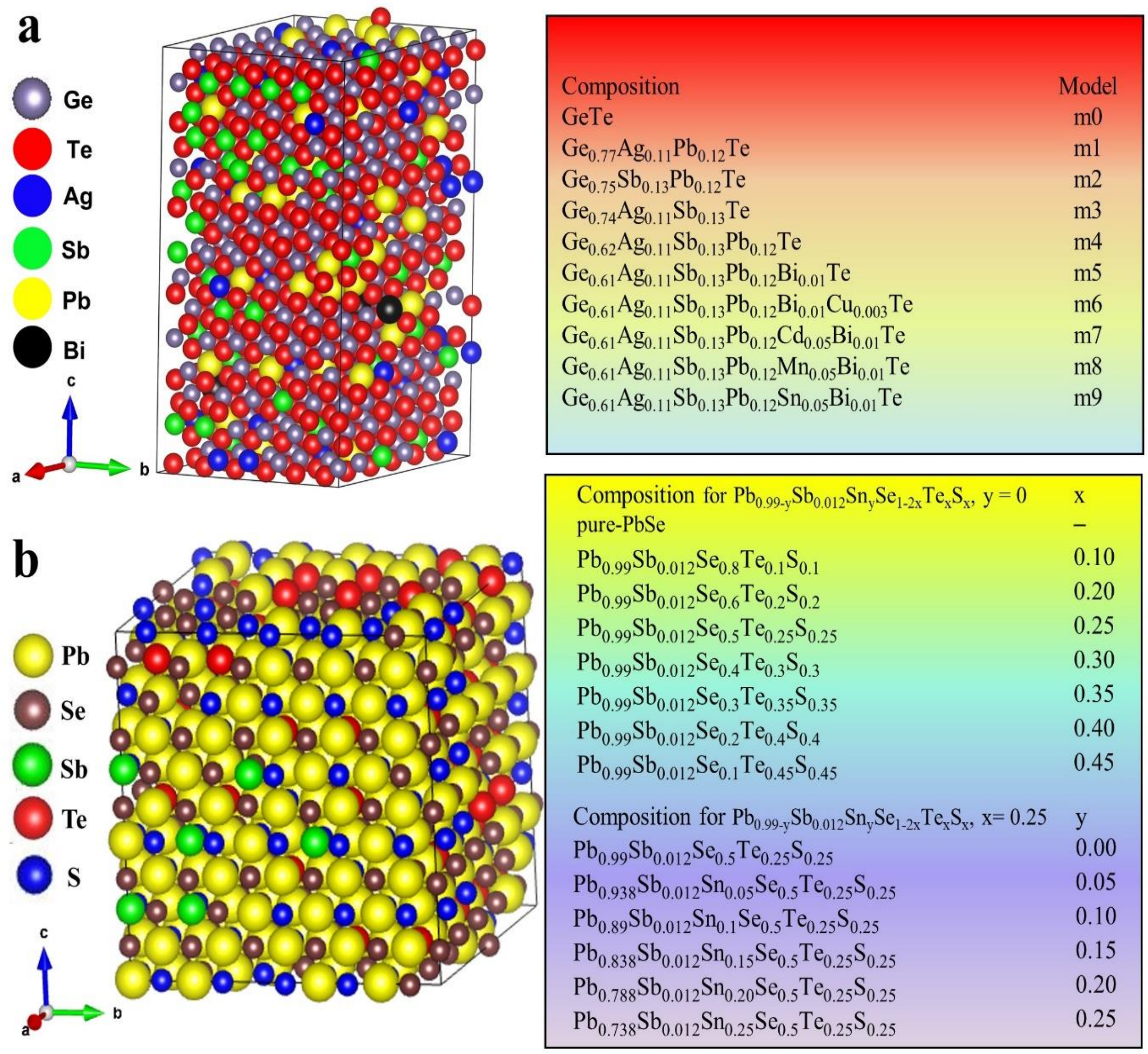

Fig. S1.
(a) Ball and stick structure of $Ge_{0.61}Ag_{0.11}Sb_{0.13}Pb_{0.12}Bi_{0.01}Te$ (m5) with their solid solution composition and model numbers in the box at right. (b) Ball and stick structure of $Pb_{0.99}Sb_{0.012}Se_{0.5}Te_{0.25}S_{0.25}$ with their solid solution composition in the box on the right.

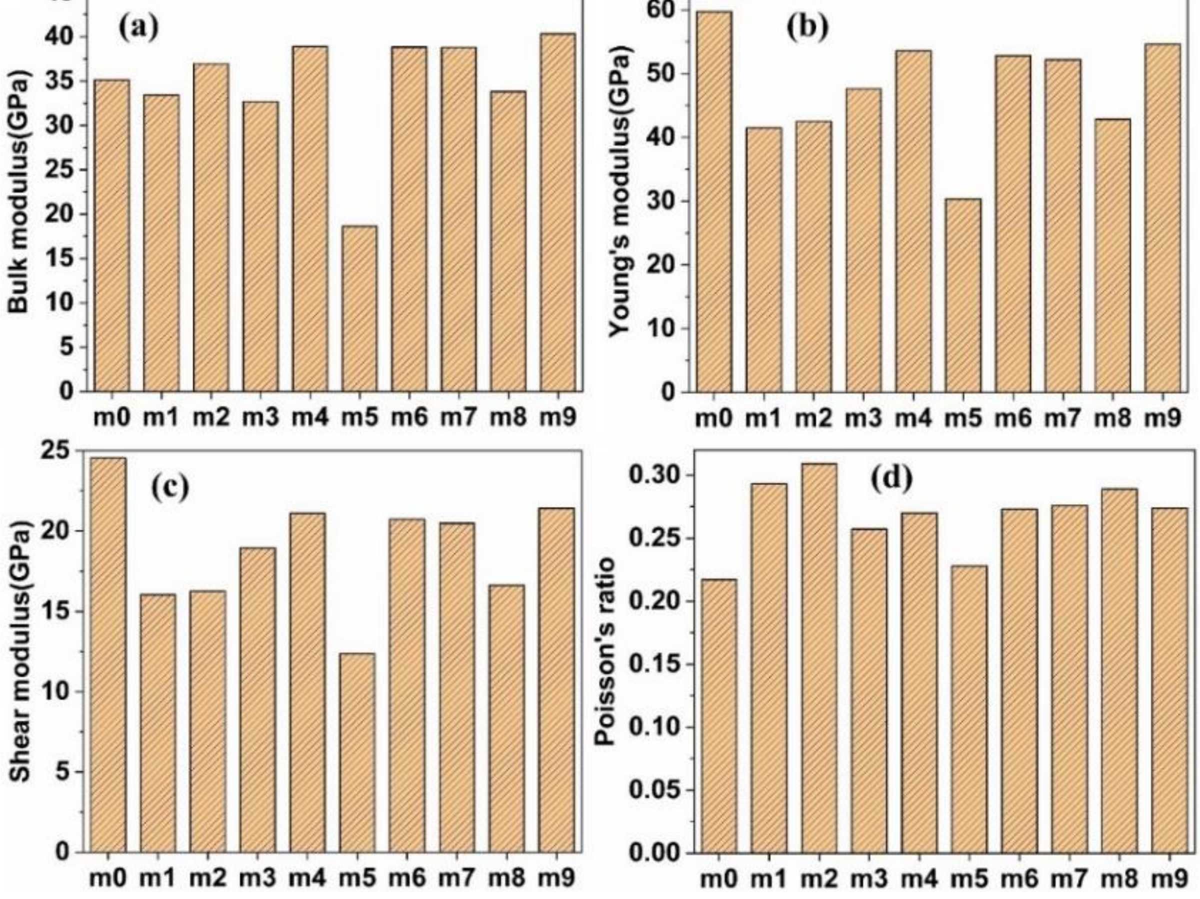

Fig. S2.
(a) Bulk modulus (K), (b) Young's modulus (E), (c) shear modulus (G), and (d) Poisson's ratio (η) for ten models in Ge-Te based high-entropy chalcogenides.

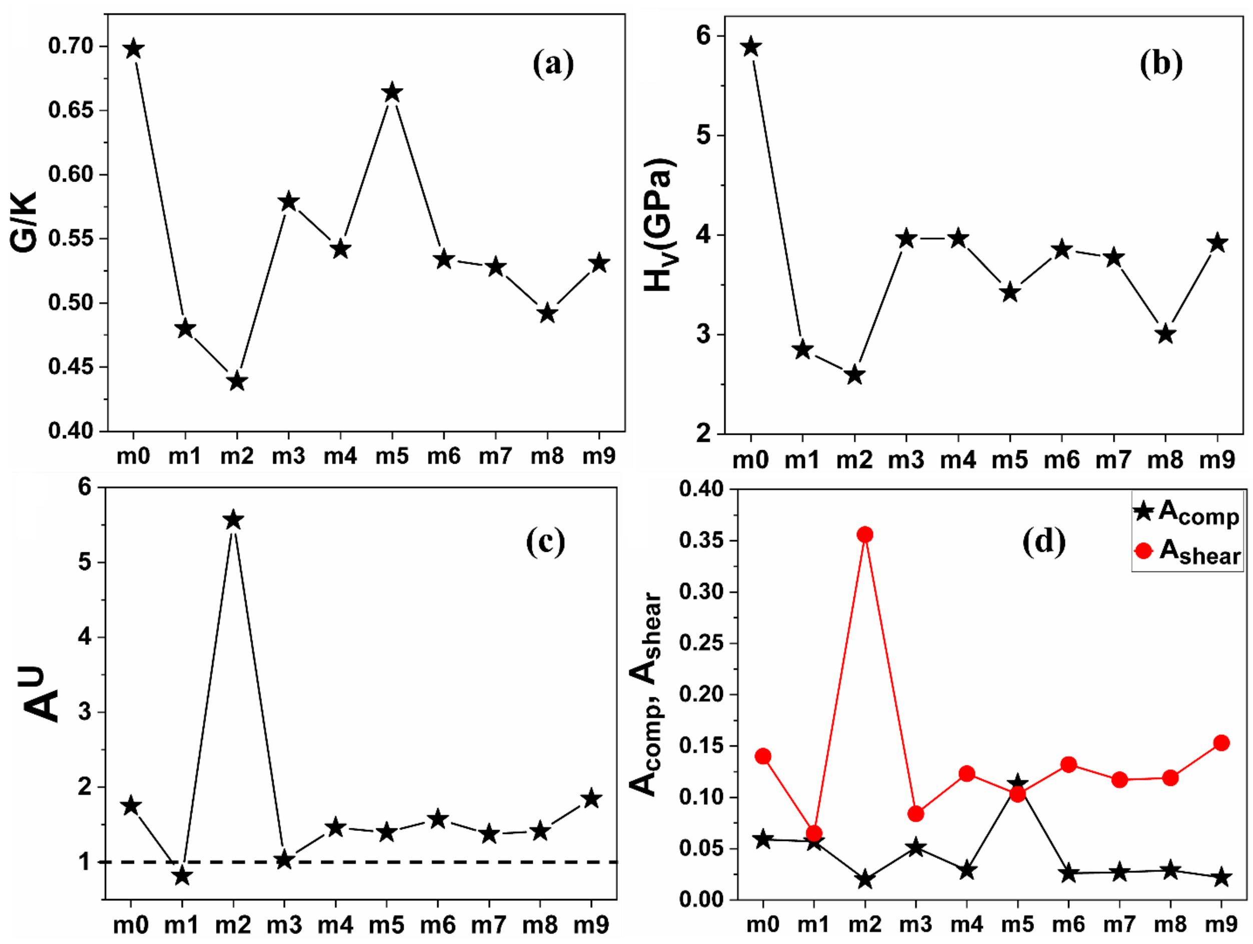

Fig. S3.
(a) Pugh's ratio (G/K), (b) Vicker's hardness (H_V), (c) the universal anisotropic index (A^U), and (d) the % of anisotropy in the compression and shear (A_{comp}, A_{shear}) for ten models in Ge-Te based high-entropy chalcogenides.

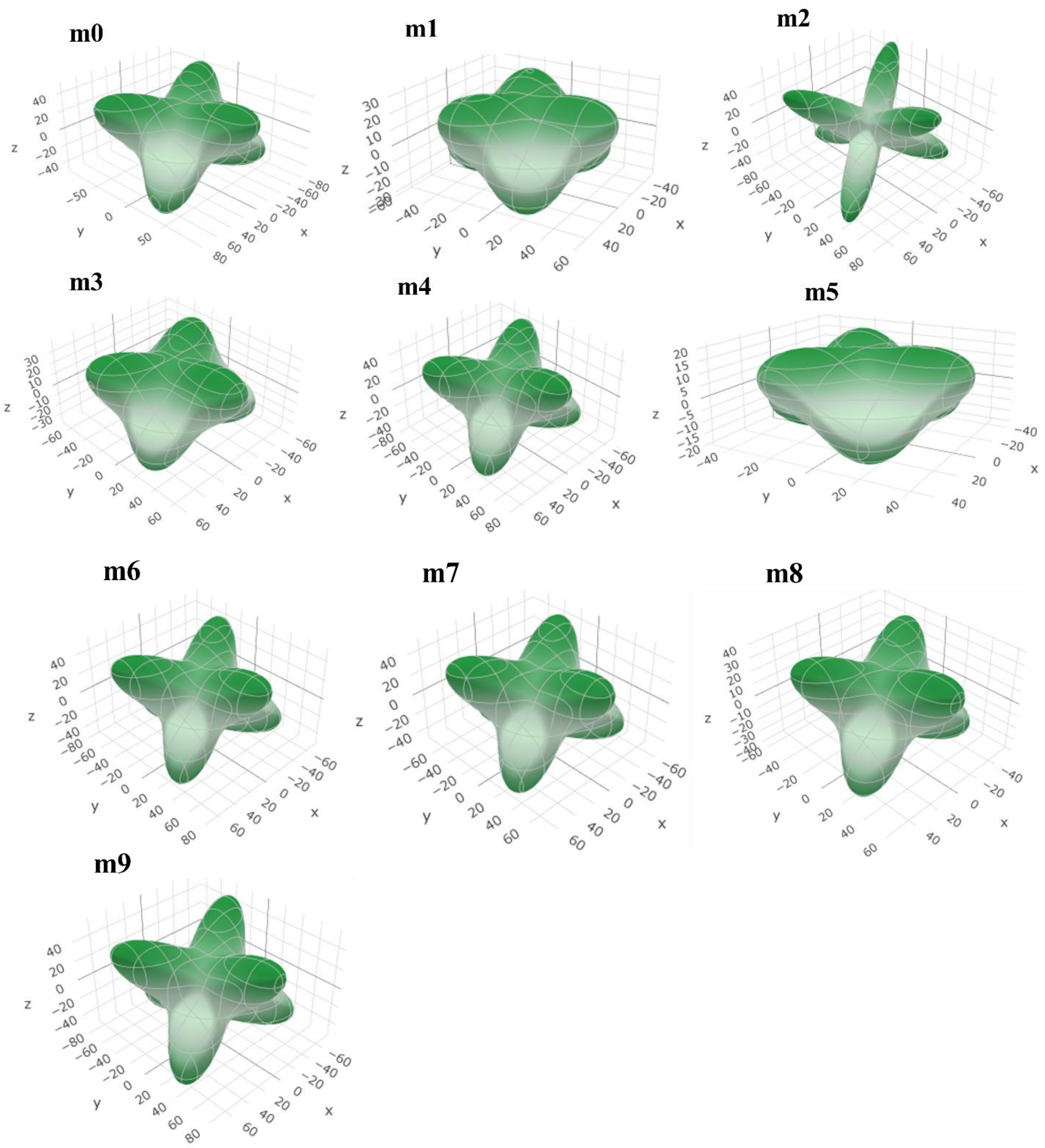

Fig. S4

The three-dimensional contour plots of the Young's modulus for m0 and other nine solid solutions.

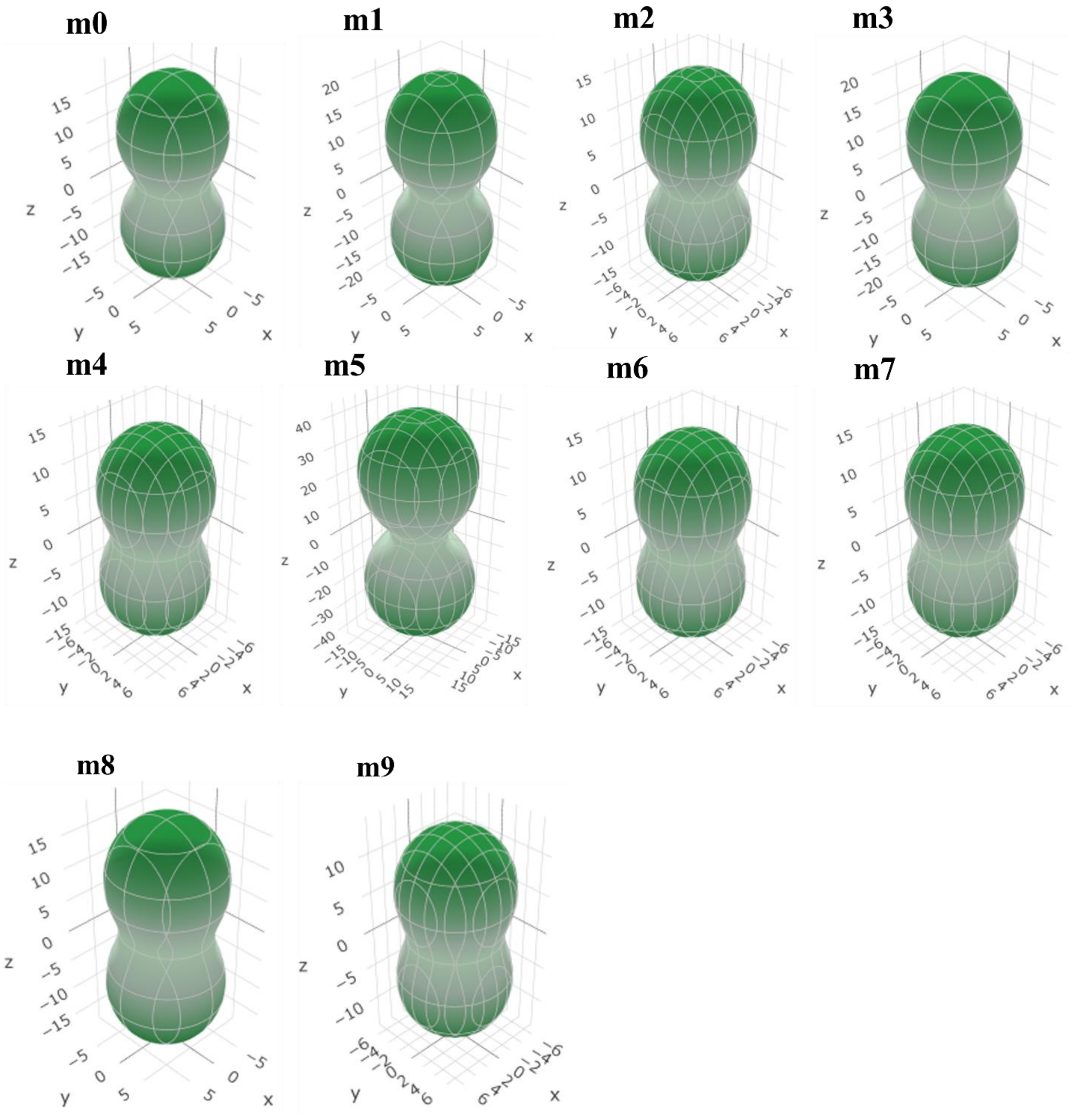

Fig. S5

The three-dimensional contour plots of the linear compressibility (1/K) for m0 and other nine solid solutions.

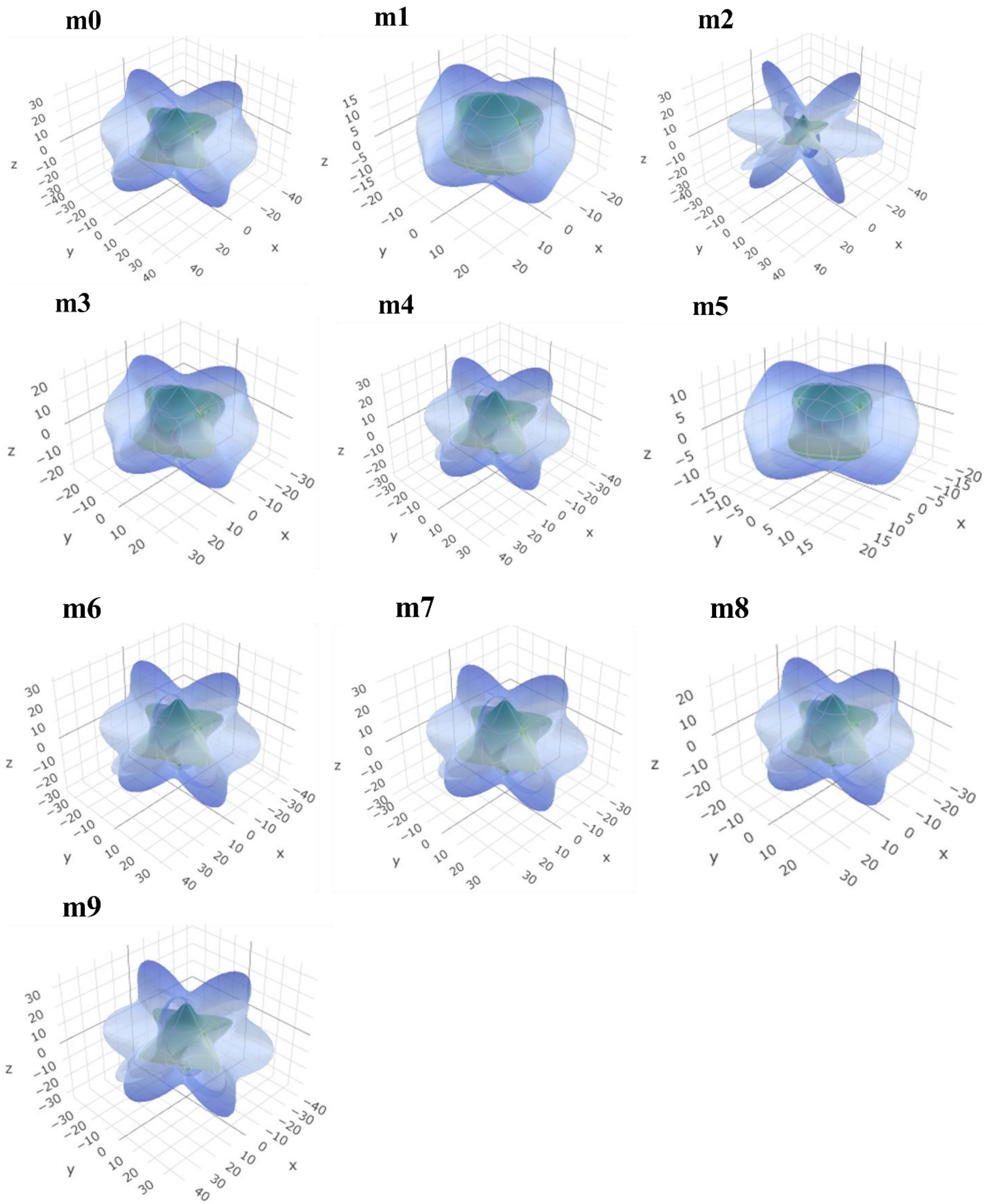

Fig. S6 the three-dimensional contour plots of shear modulus for m0 and other nine solid solutions.

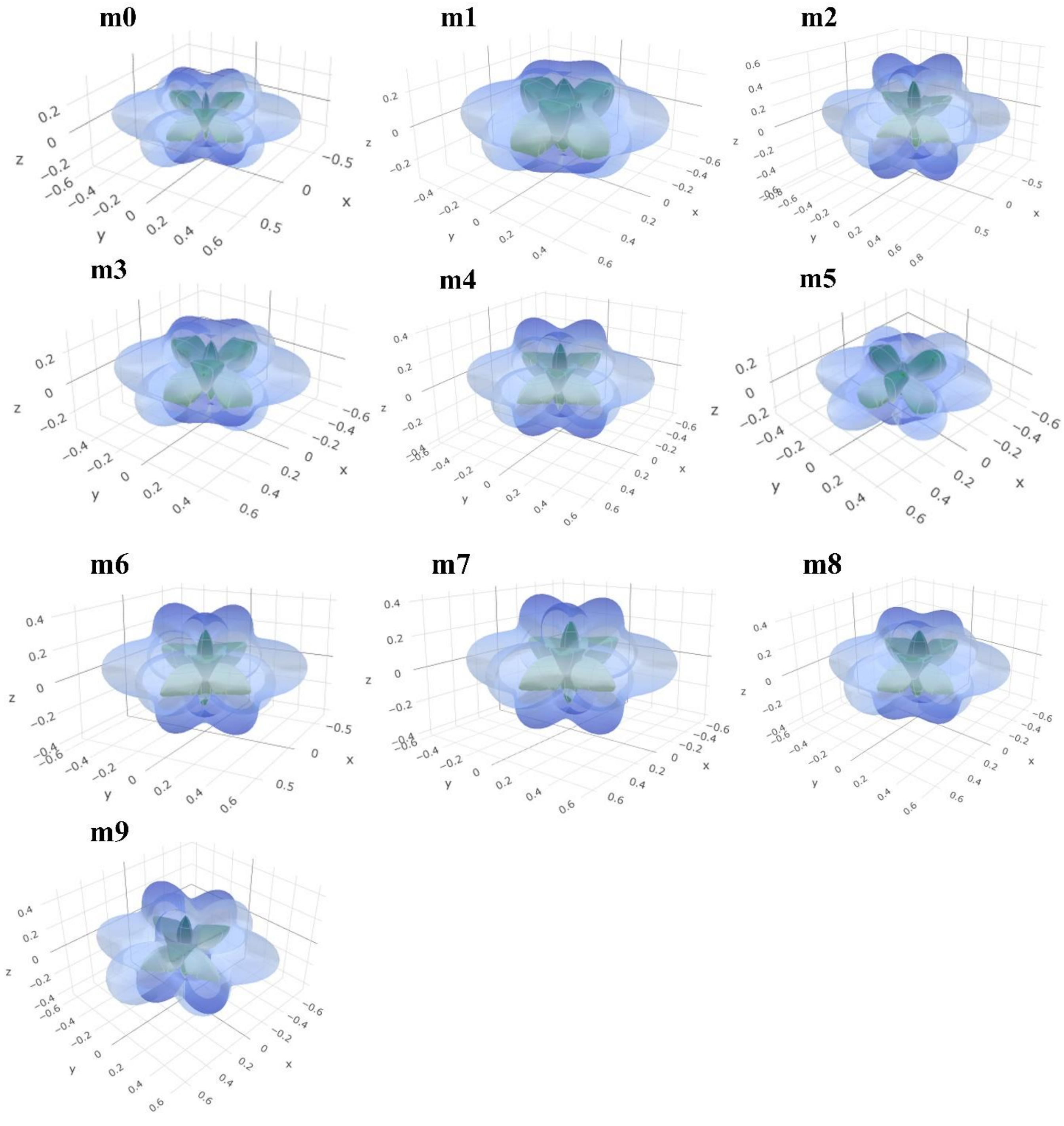

Fig. S7
The three-dimensional contour plots of the Poisson's ratio for m0 and other nine solid solutions.

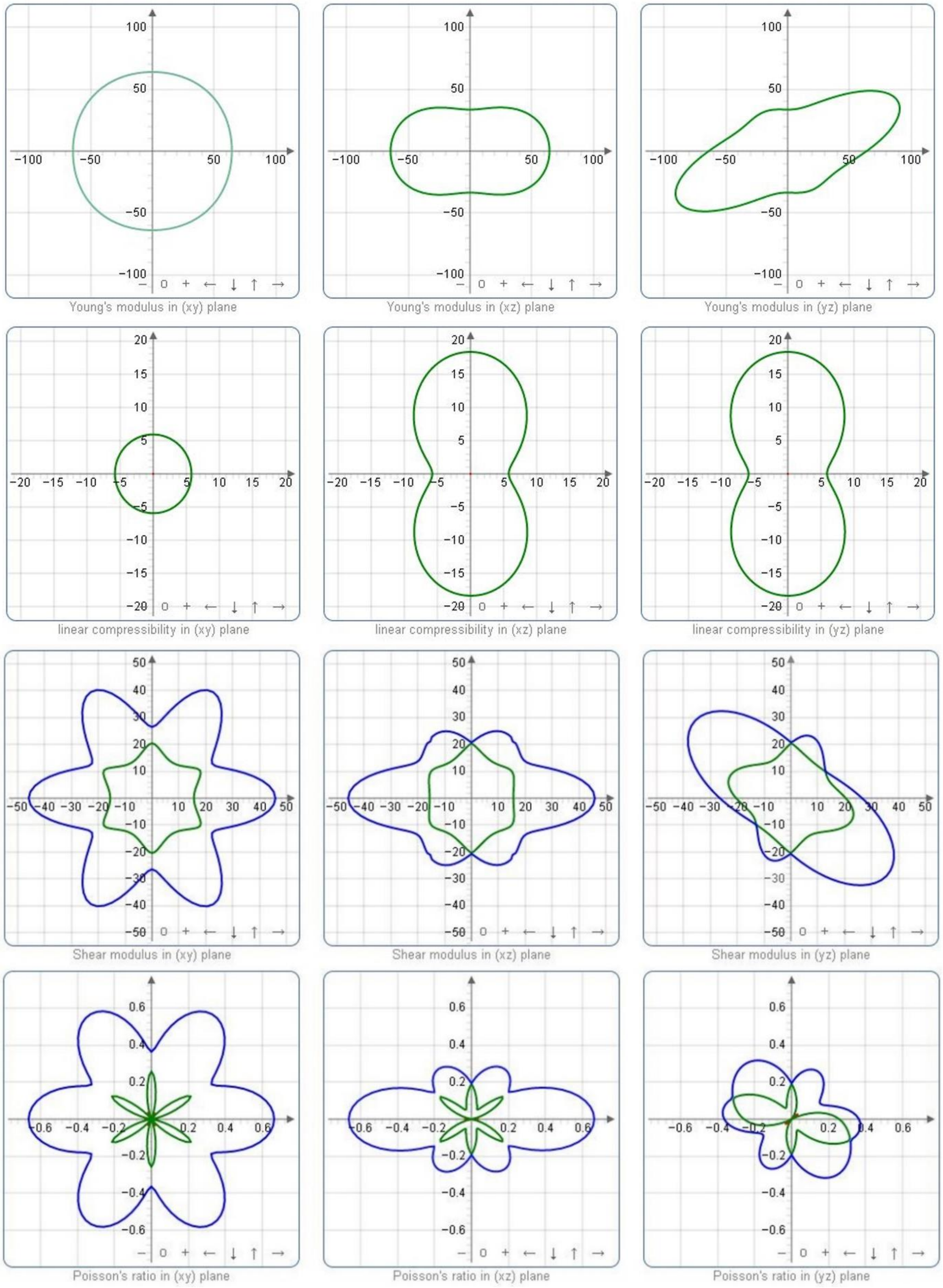

Fig. S8 the two-dimensional (2D) plot of the E, 1/K, G, and η for m0.

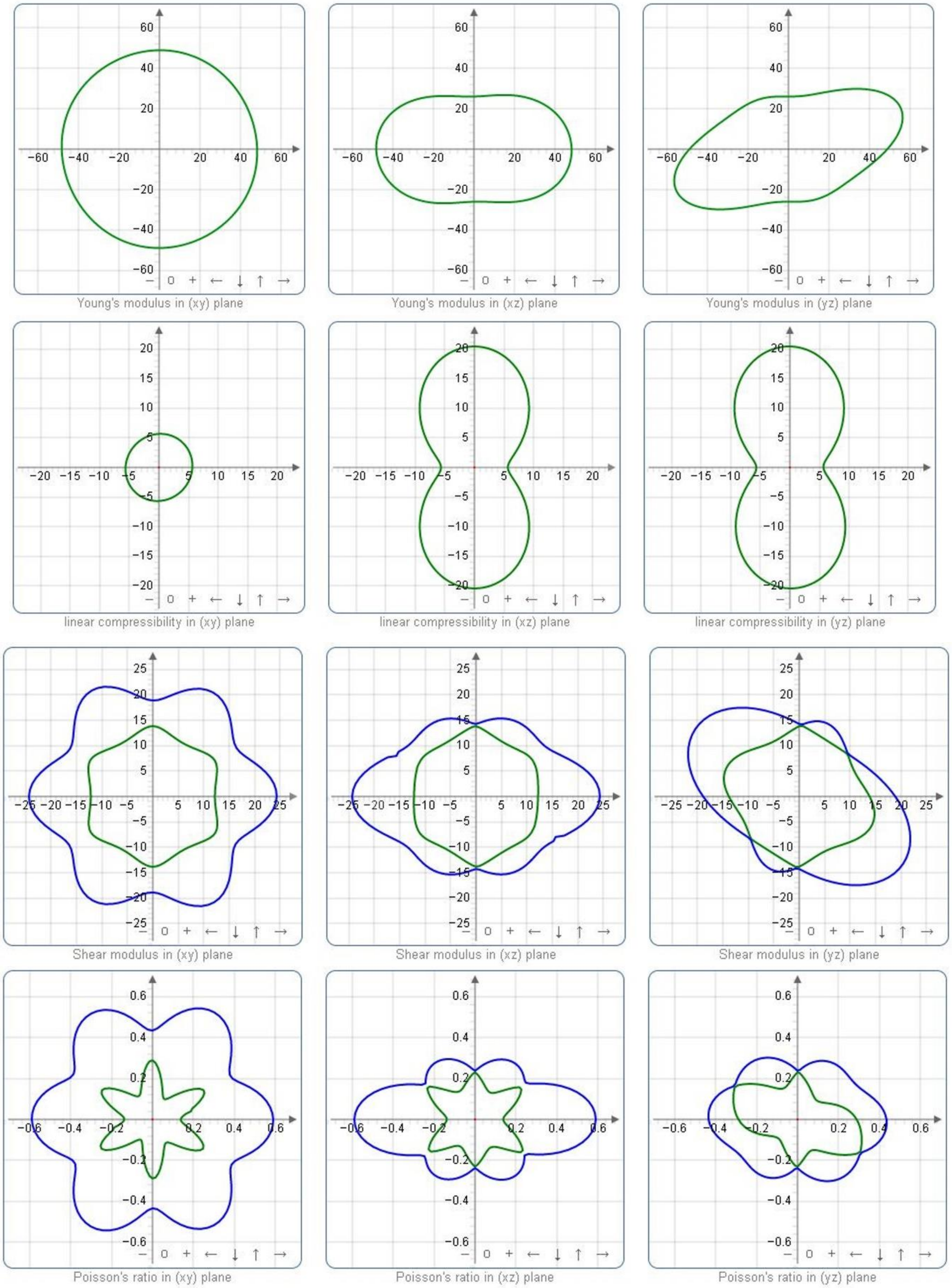

Fig. S9 the two-dimensional (2D) plot of the E, 1/K, G, and η for m1.

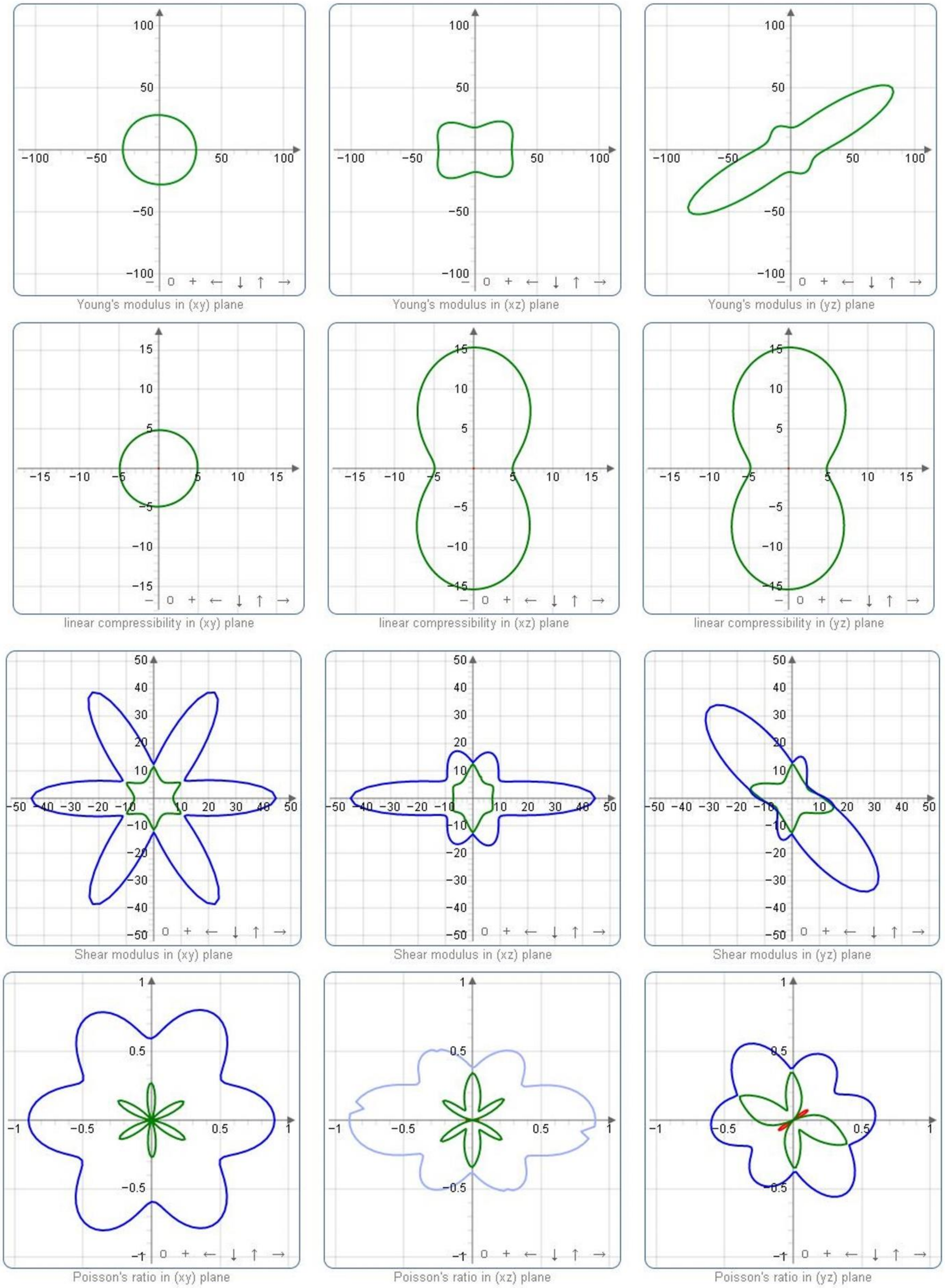

Fig. S10 the two-dimensional (2D) plot of the E, 1/K, G, and η for m2.

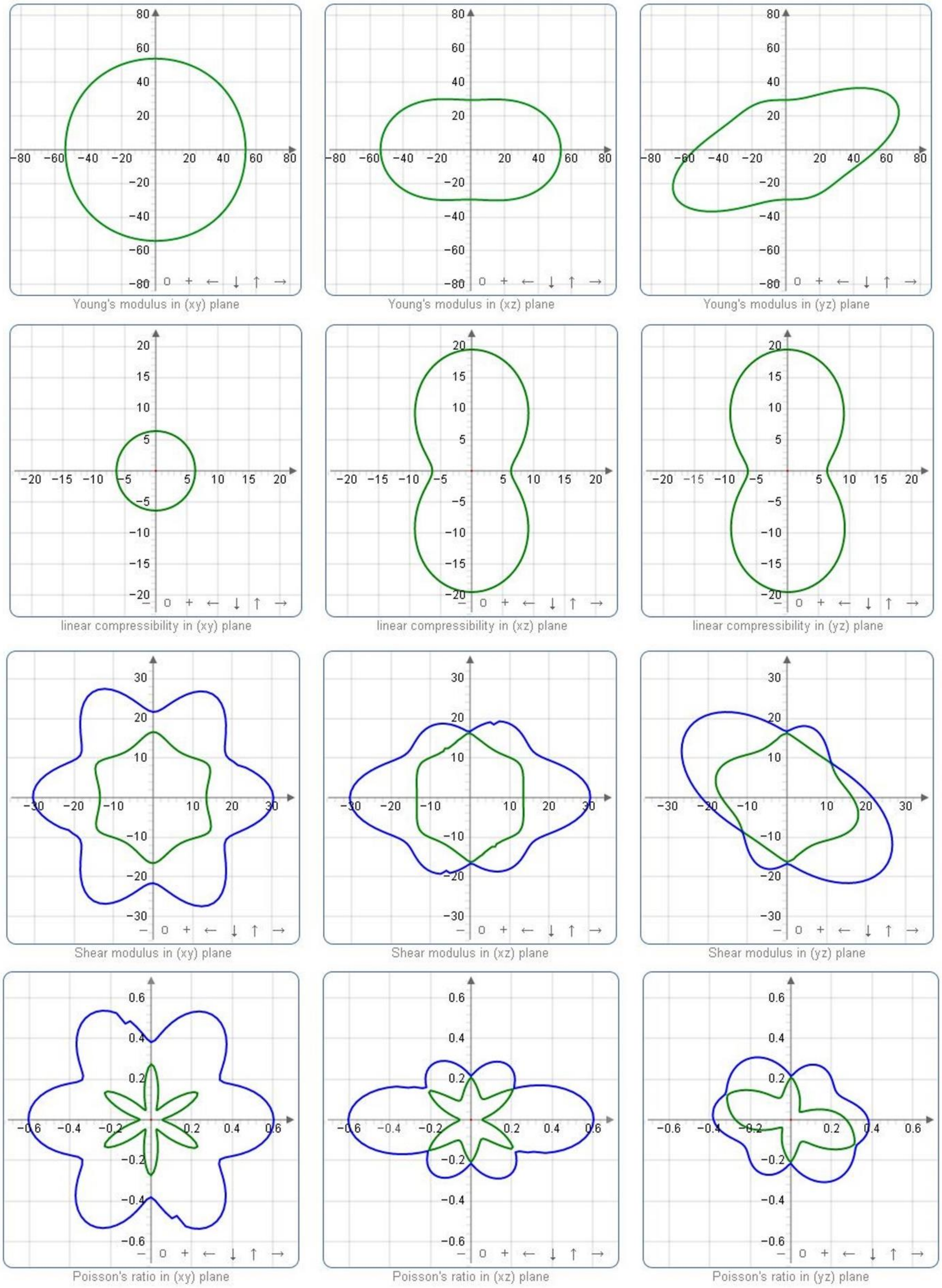

Fig. S11 the two-dimensional (2D) plot of the E, 1/K, G, and η for m3.

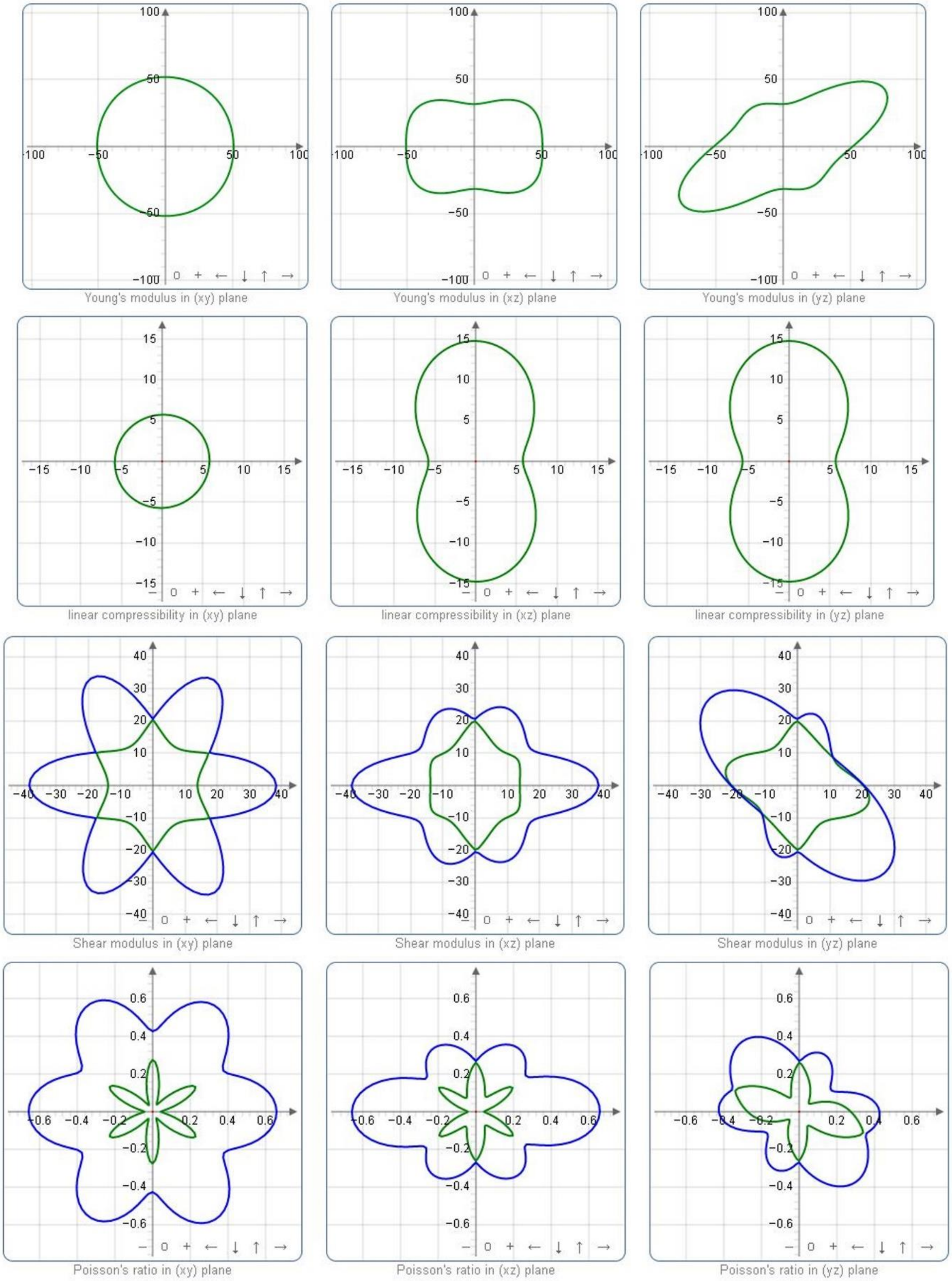

Fig. S12 the two-dimensional (2D) plot of the E, 1/K, G, and η for m4.

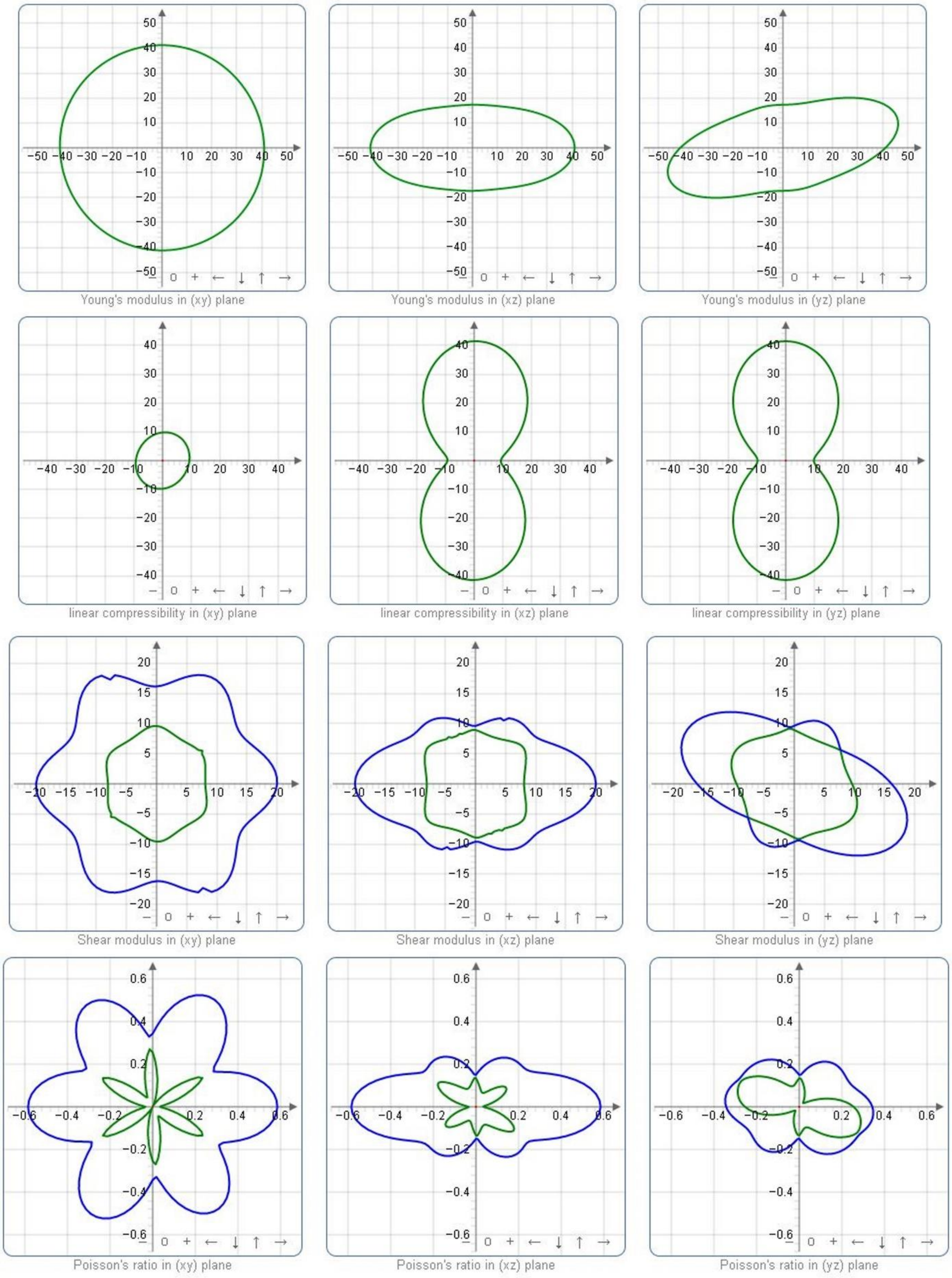

Fig. S13 the two-dimensional (2D) plot of the E, 1/K, G, and η for m5.

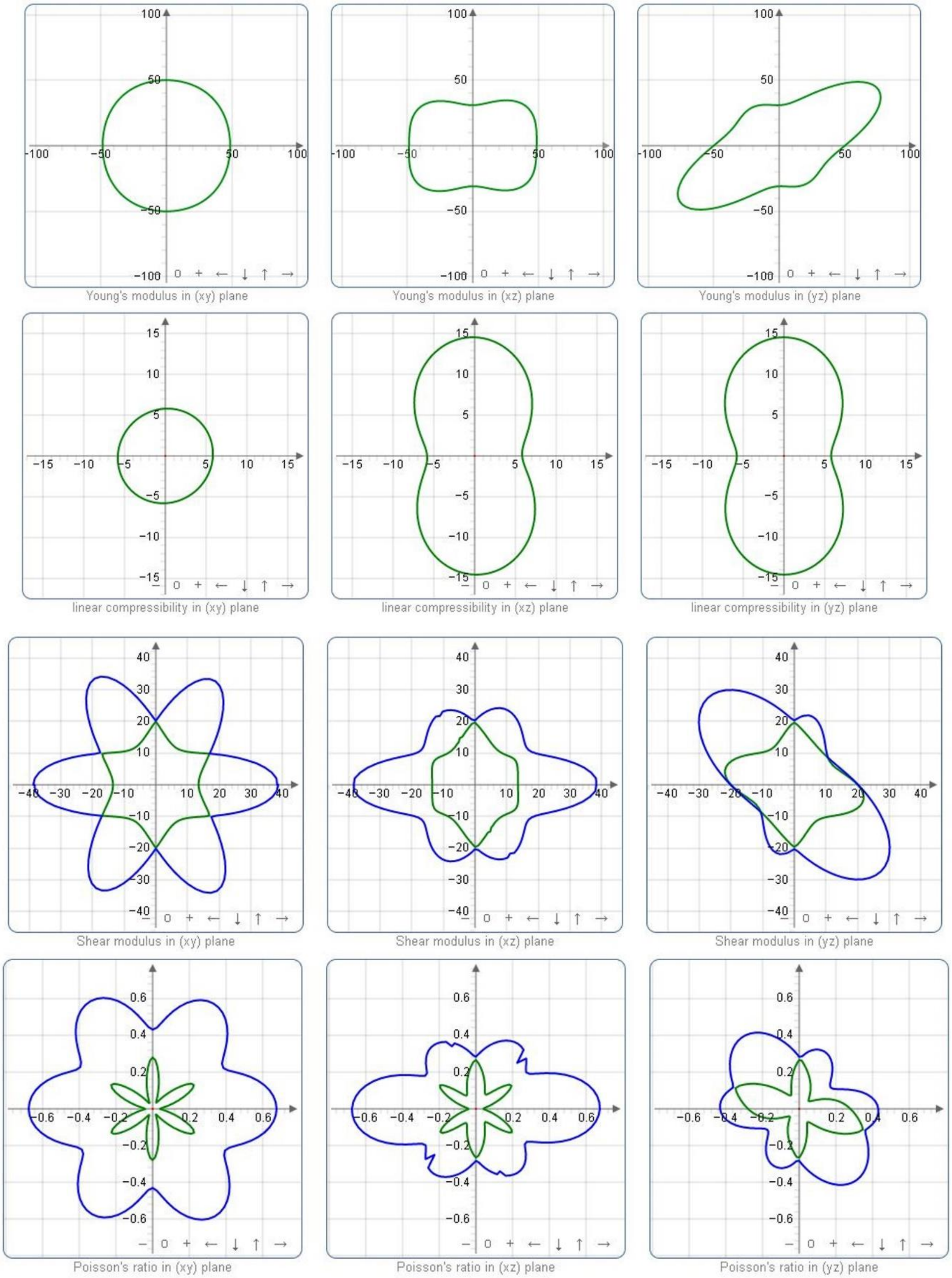

Fig. S14 the two-dimensional (2D) plot of the E, 1/K, G, and η for m6.

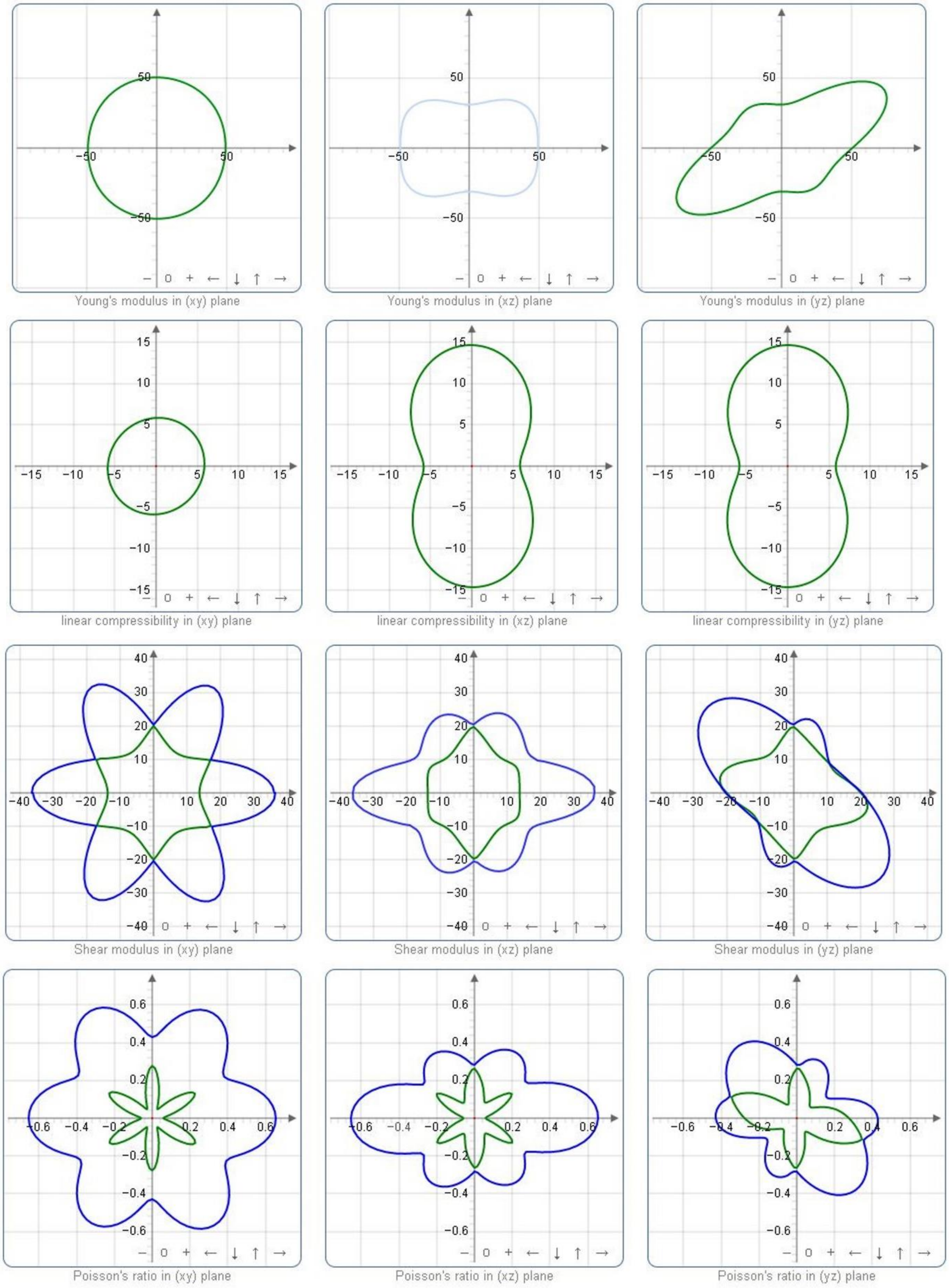

Fig. S15 the two-dimensional (2D) plot of the E, 1/K, G, and η for m7.

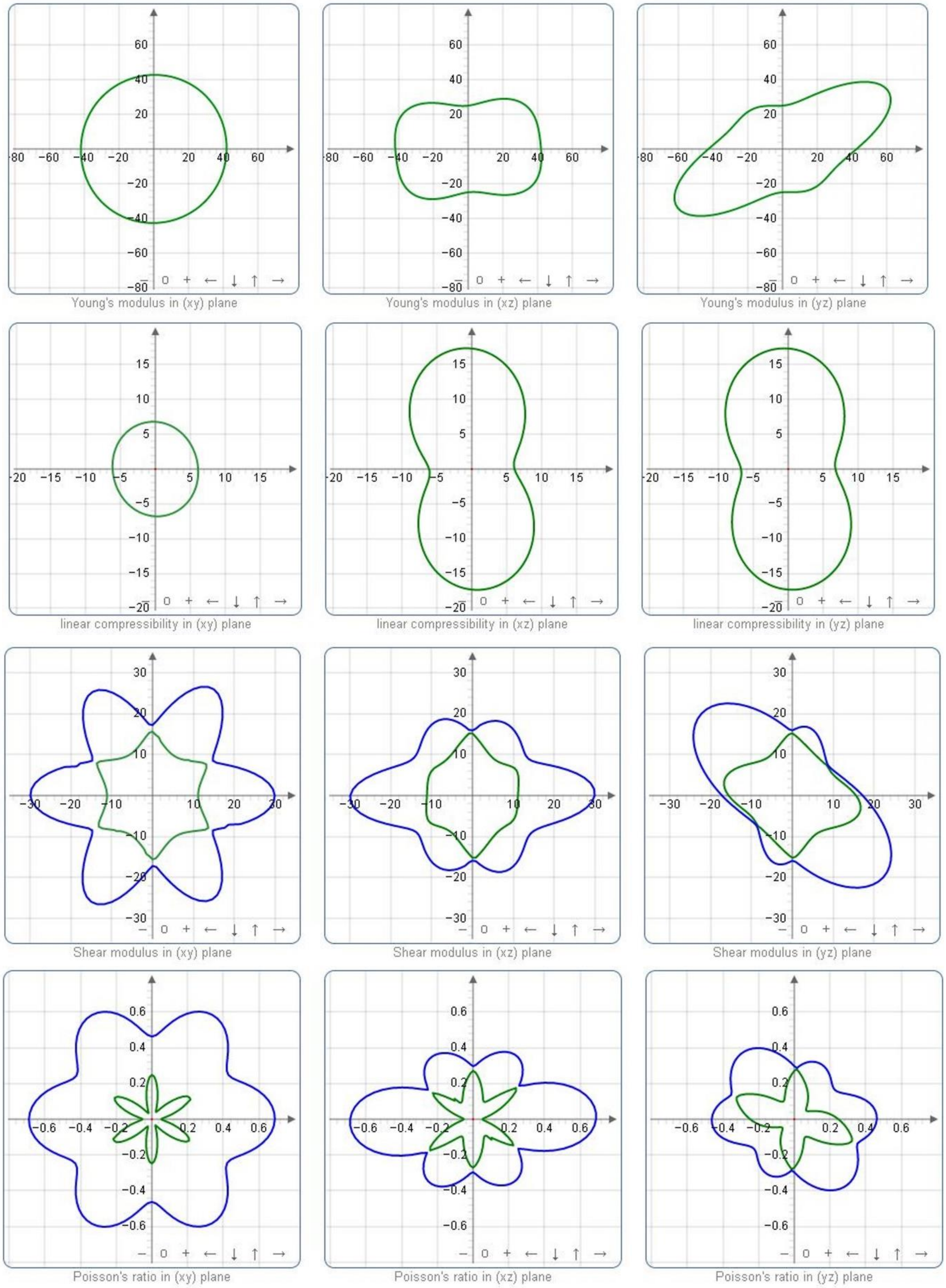

Fig. S16 the two-dimensional (2D) plot of the E, 1/K, G, and η for m8.

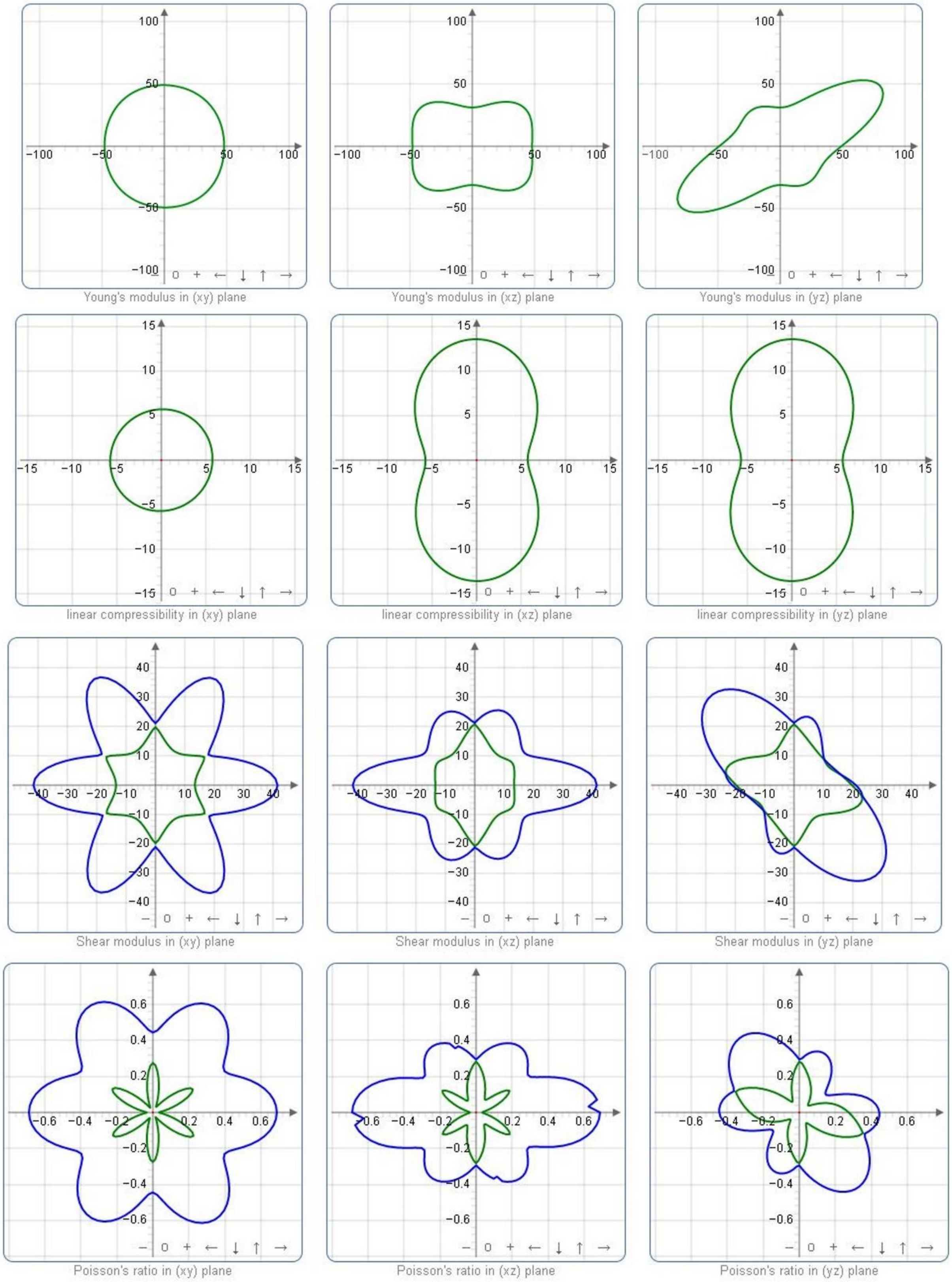

Fig. S17 the two-dimensional (2D) plot of the E, 1/K, G, and η for m9.

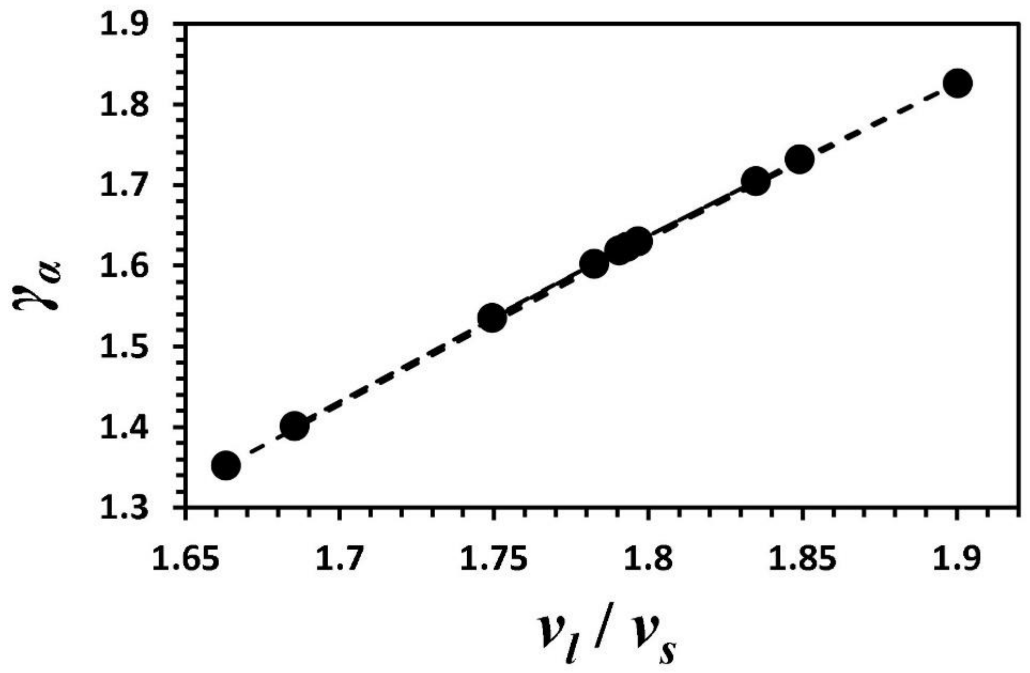

Fig. S18.
Gruneisen parameter versus the ratio v_l/v_s for the ten Ge-Te based high-entropy chalcogenide models.

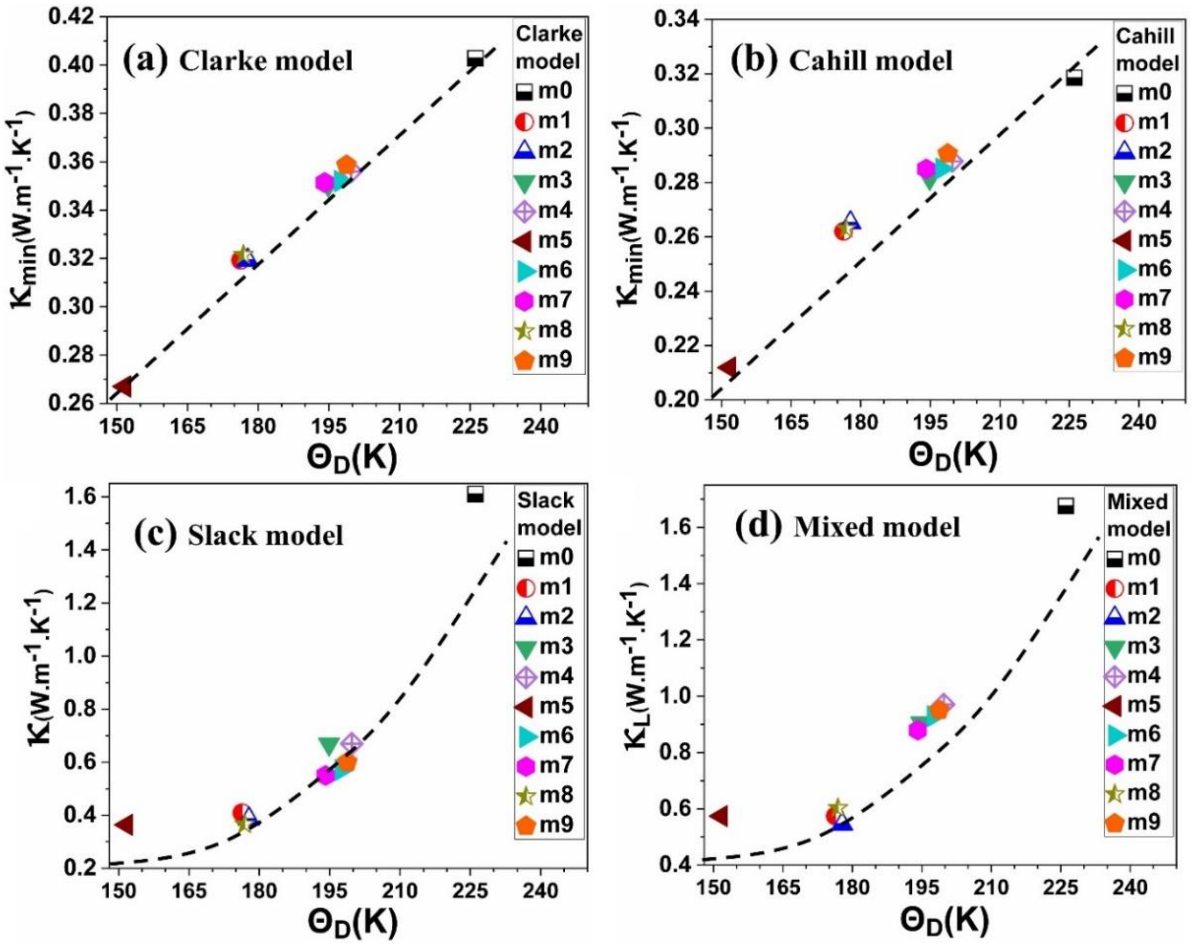

Fig. S19

Thermal conductivities vs Debye temperature for the ten Ge-Te based high-entropy chalcogenide models.

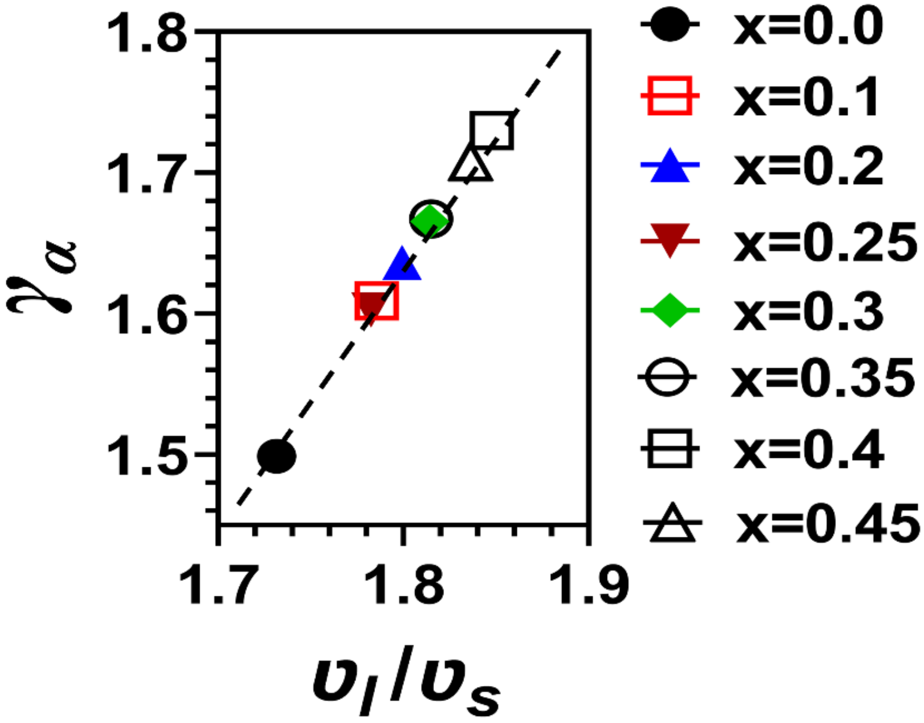

Fig. S20
Gruneisen parameter versus v_l /v_s ration for $Pb_{0.99\text{-}y}Sb_{0.012}Sn_ySe_{1\text{-}2x}Te_xS_x$ solid solutions(x=0.1, 0.2, 0.25, 0.3, 0.35, 0.4, 0.45) and y=0.

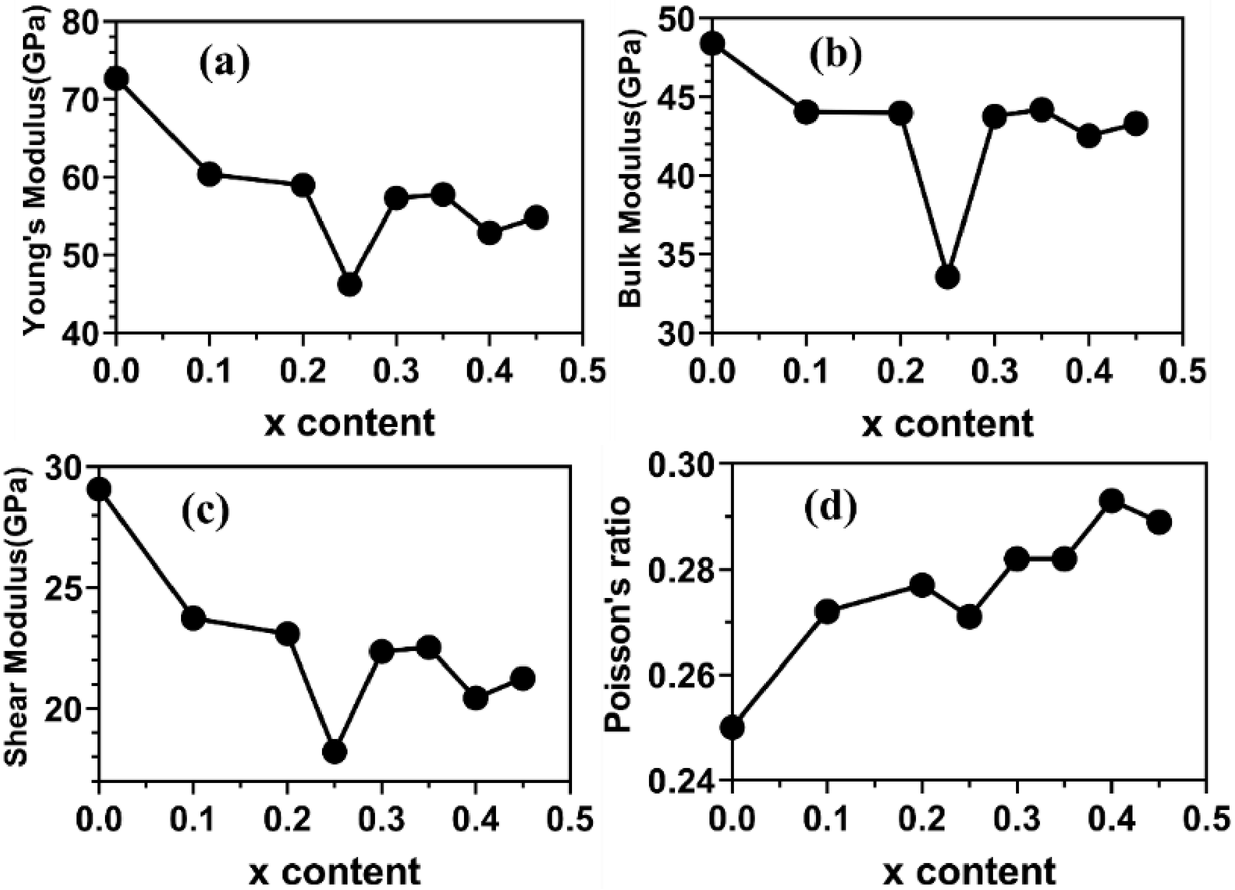

Fig. S21

(a) Young's modulus (E), (b) Bulk modulus (K) (c) shear modulus (G), and (d) Poisson's ratio (η) for $Pb_{0.99-y}Sb_{0.012}Sn_ySe_{1-2x}Te_xS_x$ solid solutions versus x content (x=0.1, 0.2, 0.25, 0.3, 0.35, 0.4, 0.45) and y=0.

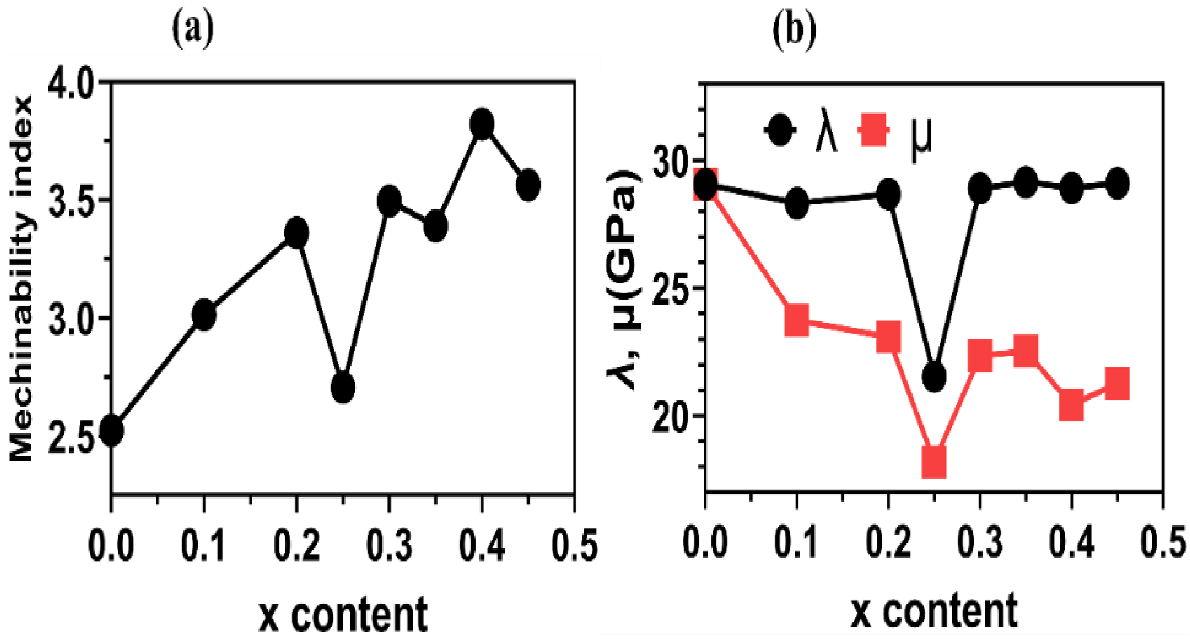

Fig. S22

(a) Machinability index, (b) Lame's constants (λ, μ) for $Pb_{0.99-y}Sb_{0.012}Sn_ySe_{1-2x}Te_xS_x$ solid solutions versus x content (x=0.1, 0.2, 0.25, 0.3, 0.35, 0.4, 0.45) and y=0.

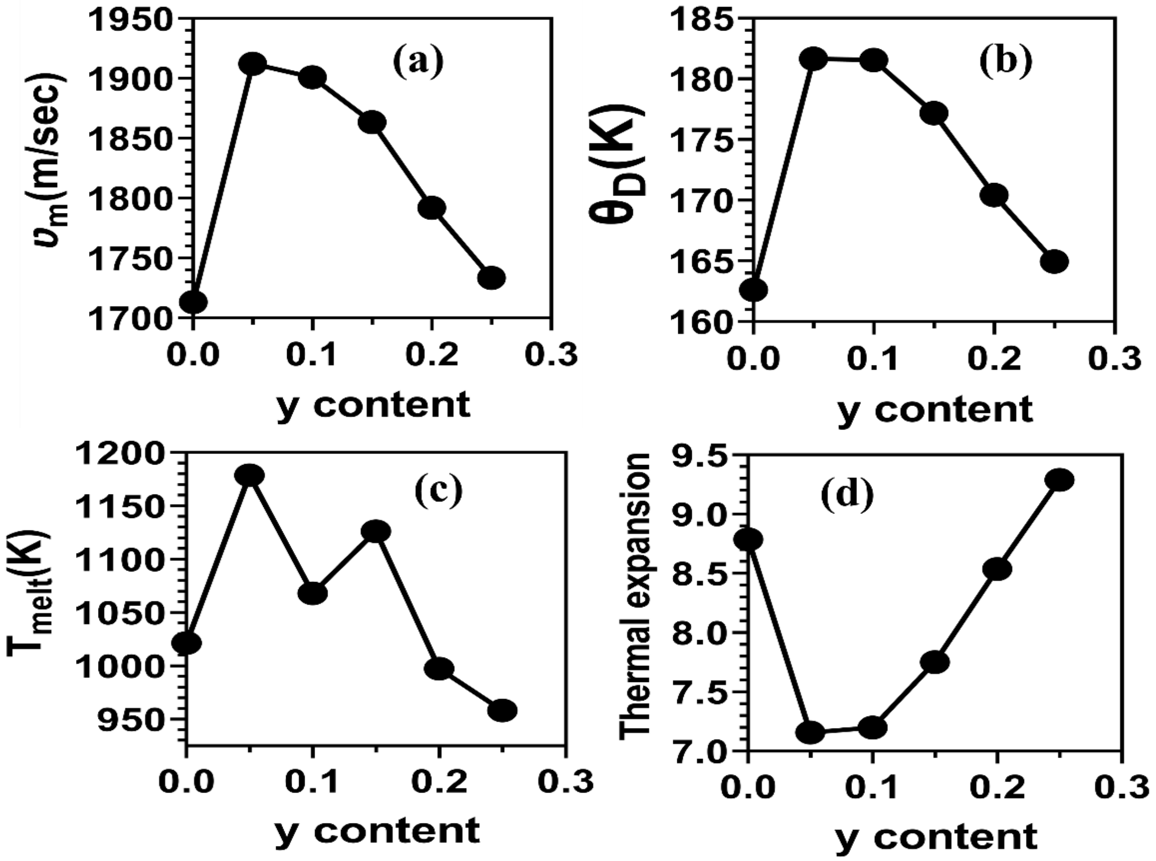

Fig. S23

(a) The average sound velocity, (b) Debye temperature, (c) melting temperature, and (d) the thermal expansion of $Pb_{0.99-y}Sb_{0.012}Sn_ySe_{1-2x}Te_xS_x$ solid solutions with respect of y content(y=0.0, 0.05, 0.1, 0.15, 0.2, 0.25) and x=0.25.

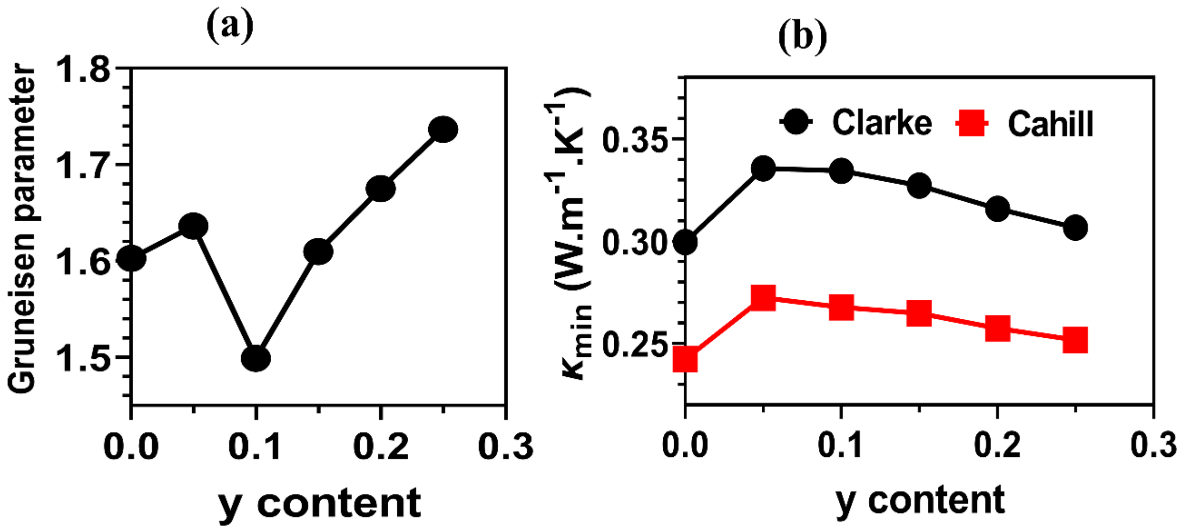

Fig. S24

(a) Acoustic Grüneisen constant. (b) Minimum thermal conductivities at 300 K for $Pb_{0.99-y}Sb_{0.012}Sn_ySe_{1-2x}Te_xS_x$ solid solutions with respect of y content(y=0.0, 0.05, 0.1, 0.15, 0.2, 0.25) and x=0.25.

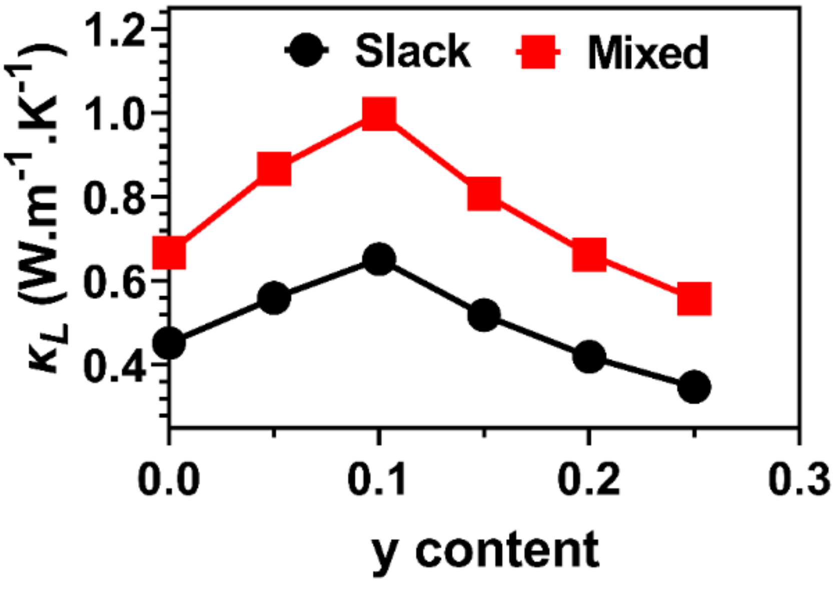

Fig. S25

Lattice thermal conductivities at 300 K with respect of y content for $Pb_{0.99-y}Sb_{0.012}Sn_ySe_{1-2x}Te_xS_x$ solid solutions(y=0.0, 0.05, 0.1, 0.15, 0.2, 0.25) and x=0.25.

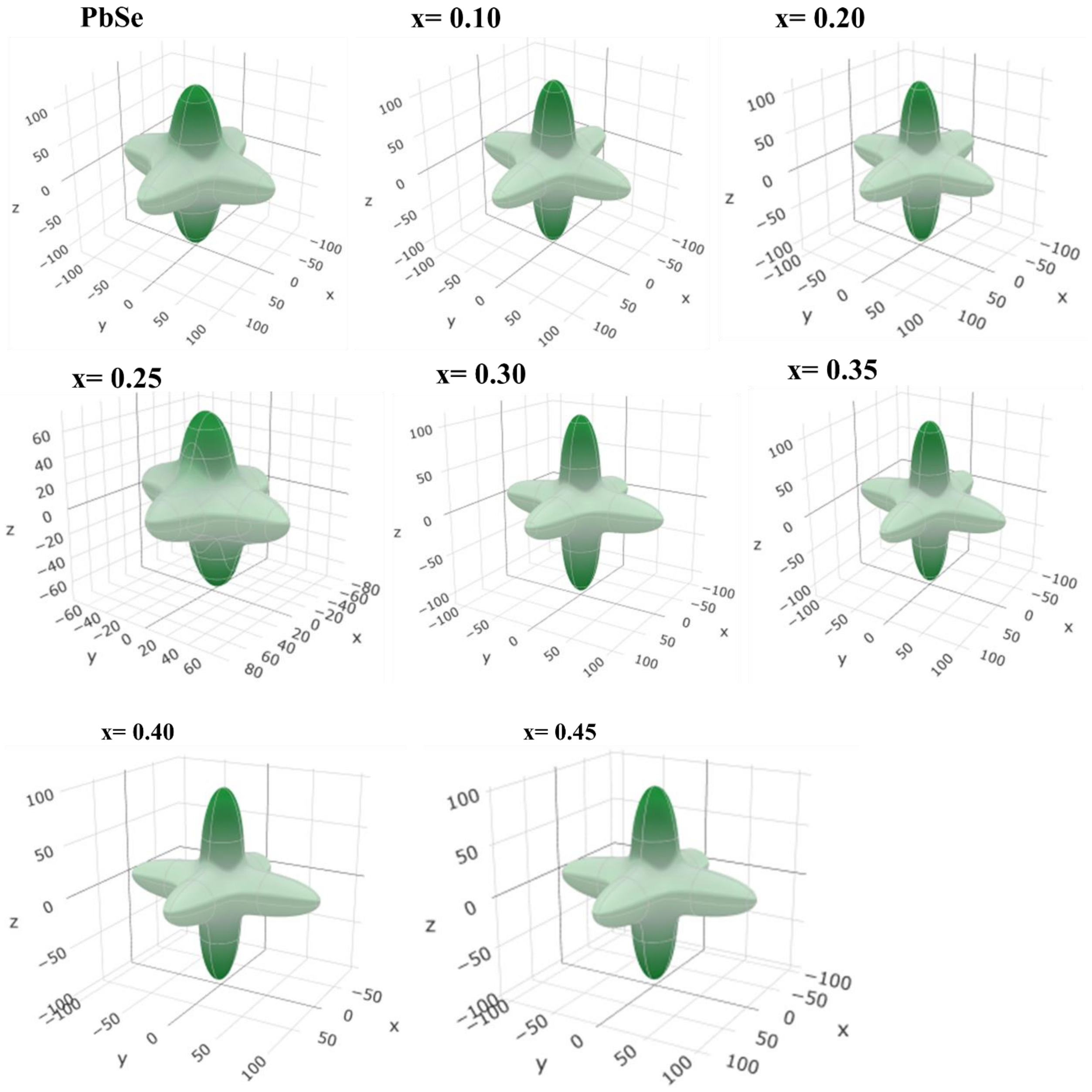

Fig. S26

The three-dimensional contour plots of the Young's modulus for $Pb_{0.99-y}Sb_{0.012}Sn_ySe_{1-2x}Te_xS_x$ solid solutions (x=0.1, 0.2, 0.25, 0.3, 0.35, 0.4, 0.45) and y=0.

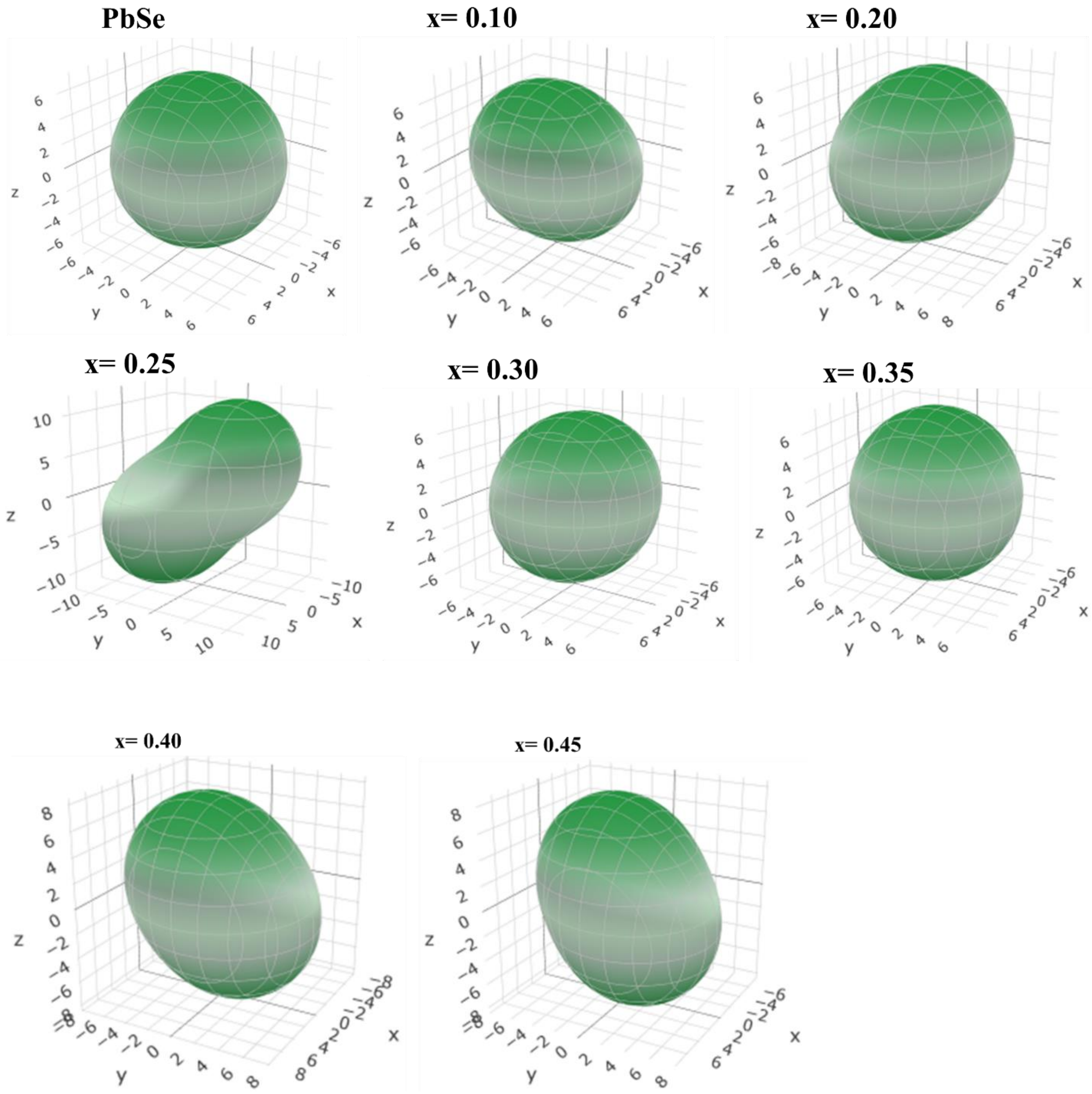

Fig. S27

The three-dimensional contour plots of the linear compressibility (1/K) for $Pb_{0.99-y}Sb_{0.012}Sn_ySe_{1-2x}Te_xS_x$ solid solutions (x=0.1, 0.2, 0.25, 0.3, 0.35, 0.4, 0.45) and y=0.

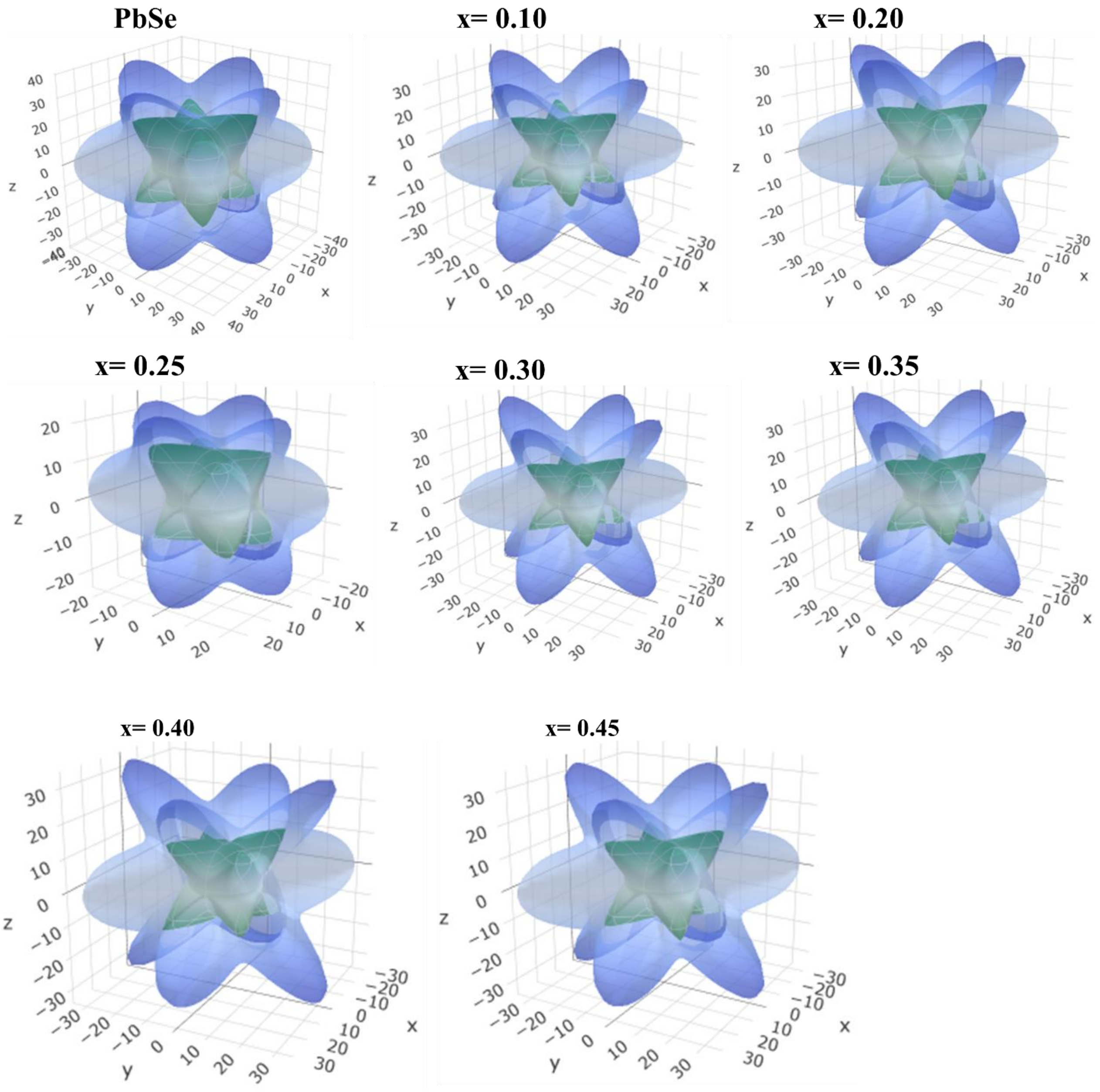

Fig. S28

The three-dimensional contour plots of the shear modulus for $Pb_{0.99-y}Sb_{0.012}Sn_ySe_{1-2x}Te_xS_x$ solid solutions (x=0.1, 0.2, 0.25, 0.3, 0.35, 0.4, 0.45) and y=0.

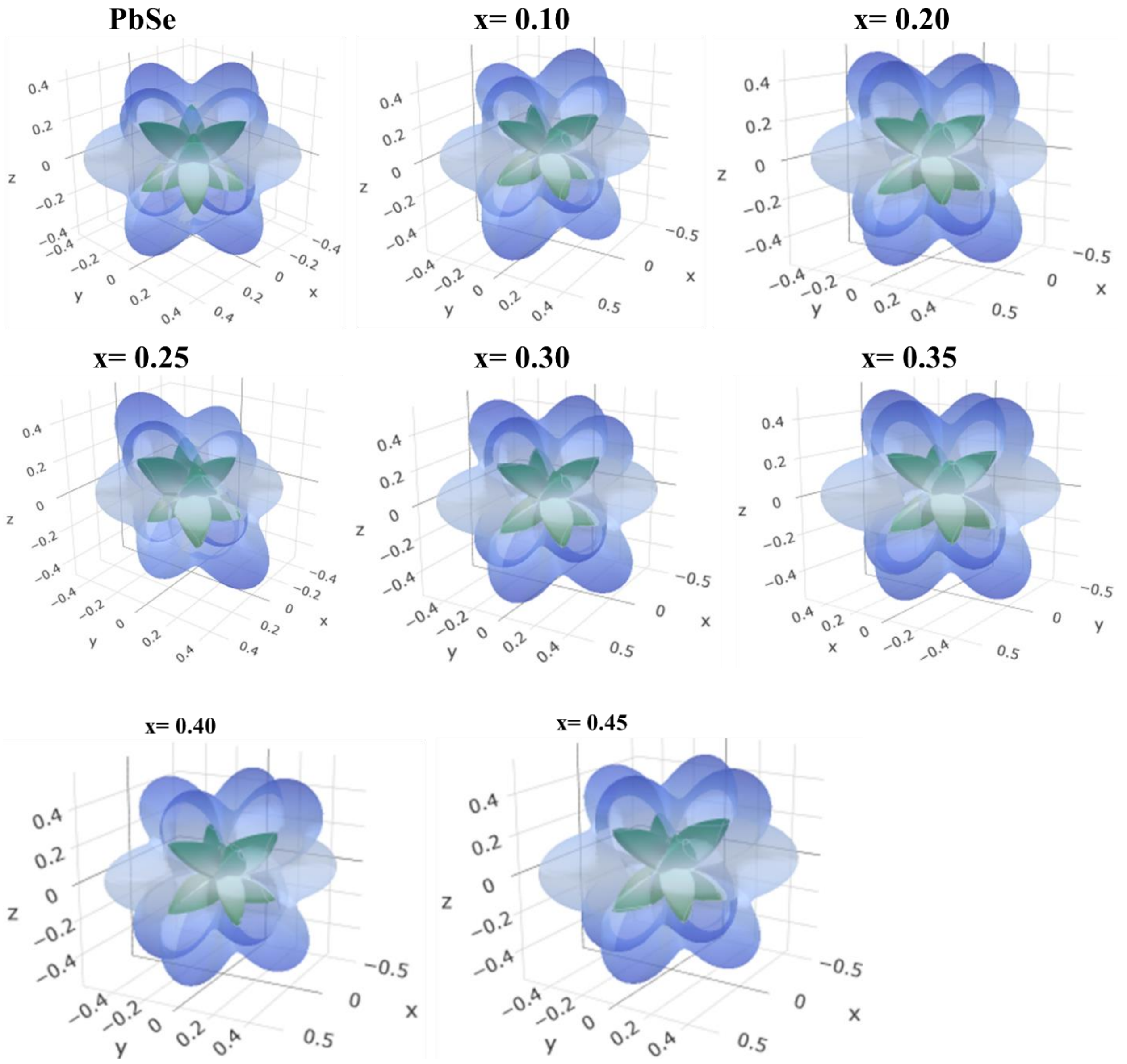

Fig. S29

The three-dimensional contour plots of the Poisson's ratio for $Pb_{0.99-y}Sb_{0.012}Sn_{y}Se_{1-2x}Te_{x}S_{x}$ solid solutions (x=0.1, 0.2, 0.25, 0.3, 0.35, 0.4, 0.45) and y=0.

Table S1.
Number of atoms for each element of the ten models in GeTe-based high-entropy chalcogenides.

Model	Composition	Number of atoms for each element
m0	GeTe	Ge (540), Te(540)
m1	$Ge_{0.77}Ag_{0.11}Pb_{0.12}Te$	Ge (416), Te(540), Ag (59) , Pb (65)
m2	$Ge_{0.75}Sb_{0.13}Pb_{0.12}Te$	Ge (405), Te(540), Sb (70) , Pb (65)
m3	$Ge_{0.74}Ag_{0.11}Sb_{0.13}Te$	Ge (411), Te(540), Ag (59) , Sb (70)
m4	$Ge_{0.62}Ag_{0.11}Sb_{0.13}Pb_{0.12}Te$	Ge (346), Te(540), Ag (59) , Sb (70), Pb (65)
m5	$Ge_{0.61}Ag_{0.11}Sb_{0.13}Pb_{0.12}Bi_{0.01}Te$	Ge (340), Te(540), Ag (59) , Sb (70), Pb (65), Bi (6)
m6	$Ge_{0.61}Ag_{0.11}Sb_{0.13}Pb_{0.12}Bi_{0.01}Cu_{0.003}Te$	Ge (338), Te(540), Ag (59) , Sb (70), Pb (65), Bi (6), Cu (2)
m7	$Ge_{0.61}Ag_{0.11}Sb_{0.13}Pb_{0.12}Cd_{0.05}Bi_{0.01}Te$	Ge (313), Te(540), Ag (59) , Sb (70), Pb (65), Bi (6), Cd (27)
m8	$Ge_{0.61}Ag_{0.11}Sb_{0.13}Pb_{0.12}Mn_{0.05}Bi_{0.01}Te$	Ge (313), Te(540), Ag (59) , Sb (70), Pb (65), Bi (6), Mn (27)
m9	$Ge_{0.61}Ag_{0.11}Sb_{0.13}Pb_{0.12}Sn_{0.05}Bi_{0.01}Te$	Ge (313), Te(540), Ag (59) , Sb (70), Pb (65), Bi (6), Sn (27)

Table S2.
Calculated lattice parameters of the ten models in GeTe based high-entropy chalcogenides.

Model	Composition	a, b, c (Å), α, β, γ (degree)
m0	GeTe (perfect crystal)	25.392, 25.392, 54.612, 90.00°, 90.00°, 120.00°
m1	$Ge_{0.77}Ag_{0.11}Pb_{0.12}Te$	25.577, 25.542, 54.707, 90.08°, 90.12°, 120.01°
m2	$Ge_{0.75}Sb_{0.13}Pb_{0.12}Te$	25.753, 25.789, 55.831, 90.34°, 89.71°, 119.82°
m3	$Ge_{0.74}Ag_{0.11}Sb_{0.13}Te$	25.612, 25.60, 54.043, 90.05°, 90.05°, 120.02°
m4	$Ge_{0.62}Ag_{0.11}Sb_{0.13}Pb_{0.12}Te$	25.946, 25.921, 54.734, 90.04°, 90.10°, 120.02°
m5	$Ge_{0.61}Ag_{0.11}Sb_{0.13}Pb_{0.12}Bi_{0.01}Te$	25.975, 25.952, 54.719, 90.06°, 90.17°, 120.01°
m6	$Ge_{0.61}Ag_{0.11}Sb_{0.13}Pb_{0.12}Bi_{0.01}Cu_{0.003}Te$	25.978, 25.959, 54.699, 89.99°, 90.24°, 120.02°
m7	$Ge_{0.61}Ag_{0.11}Sb_{0.13}Pb_{0.12}Cd_{0.05}Bi_{0.01}Te$	26.0335, 25.988, 54.468, 89.97°, 90.26°, 119.99°
m8	$Ge_{0.61}Ag_{0.11}Sb_{0.13}Pb_{0.12}Mn_{0.05}Bi_{0.01}Te$	25.840, 25.846, 54.004, 90.19°, 89.85°, 120.02°
m9	$Ge_{0.61}Ag_{0.11}Sb_{0.13}Pb_{0.12}Sn_{0.05}Bi_{0.01}Te$	26.054, 26.039, 54.800, 90.03°, 90.16°, 120.00°

Table S3.

Calculated lattice parameters for $Pb_{0.99-y}Sb_{0.012}Sn_ySe_{1-2x}Te_xS_x$, y = 0.

x	Model	a, b, c(Å), α, β, γ(degree)	Vol. (Å^3)
–	pure-PbSe	31.031, 31.031, 31.031, 90.00, 90.00, 90.00	29880.46
0.10	$Pb_{0.99}Sb_{0.012}Se_{0.8}Te_{0.1}S_{0.1}$	31.100, 31.167, 31.174, 89.97, 90.02, 89.99	30216.76
0.20	$Pb_{0.99}Sb_{0.012}Se_{0.6}Te_{0.2}S_{0.2}$	31.213, 31.301, 31.324, 89.86, 90.01, 89.99	30603.49
0.25	$Pb_{0.99}Sb_{0.012}Se_{0.5}Te_{0.25}S_{0.25}$	31.307, 31.396, 31.394, 89.69, 90.16, 90.26	30857.62
0.30	$Pb_{0.99}Sb_{0.012}Se_{0.4}Te_{0.3}S_{0.3}$	31.392, 31.453, 31.444, 89.96, 90.00, 90.08	31046.94
0.35	$Pb_{0.99}Sb_{0.012}Se_{0.3}Te_{0.35}S_{0.35}$	31.439, 31.485, 31.588, 90.17, 90.01, 90.04	31267.60
0.40	$Pb_{0.99}Sb_{0.012}Se_{0.2}Te_{0.4}S_{0.4}$	31.530, 31.562, 31.650, 90.25, 90.07, 89.87	31496.49
0.45	$Pb_{0.99}Sb_{0.012}Se_{0.1}Te_{0.45}S_{0.45}$	31.561, 31.672, 31.649, 90.07, 89.96, 89.99	31636.34

Table S4.

Calculated lattice parameters for $Pb_{0.99-y}Sb_{0.012}Sn_ySe_{1-2x}Te_xS_x$, x= 0.25.

y	Model	a, b, c(Å), α, β, γ(degree)	Vol.(Å^3)
0.00	$Pb_{0.99}Sb_{0.012}Se_{0.5}Te_{0.25}S_{0.25}$	31.307, 31.396, 31.394, 89.69, 90.16, 90.26	30857.62
0.05	$Pb_{0.938}Sb_{0.012}Sn_{0.05}Se_{0.5}Te_{0.25}S_{0.25}$	31.279, 31.380, 31.349, 89.96, 89.99, 90.01	30770.14
0.10	$Pb_{0.89}Sb_{0.012}Sn_{0.1}Se_{0.5}Te_{0.25}S_{0.25}$	31.215, 31.176, 31.189, 90.01, 90.09, 89.96	30351.85
0.15	$Pb_{0.838}Sb_{0.012}Sn_{0.15}Se_{0.5}Te_{0.25}S_{0.25}$	31.188, 31.386, 31.353, 90.30, 90.04, 90.03	30690.41
0.20	$Pb_{0.788}Sb_{0.012}Sn_{0.20}Se_{0.5}Te_{0.25}S_{0.25}$	31.183, 31.378, 31.343, 90.29, 90.17, 89.94	30667.88
0.25	$Pb_{0.738}Sb_{0.012}Sn_{0.25}Se_{0.5}Te_{0.25}S_{0.25}$	31.167, 31.346, 31.340, 90.29, 90.19, 89.86	30617.95

Table S5.

The calculated elastic constants C_{ij} (GPa) for ten models in Ge-Te based high-entropy chalcogenides.

Model	C_{11}	C_{12}	C_{13}	C_{21}	C_{22}	C_{23}	C_{31}	C_{32}	C_{33}	C_{44}	C_{55}	C_{66}
m0	87.27	19.73	20.57	19.73	86.77	20.32	20.57	20.32	41.42	26.86	26.95	34.43
m1	68.14	27.31	22.53	27.31	68.99	22.42	22.53	22.42	36.56	15.09	15.72	20.80
m2	72.95	23.58	34.67	23.58	71.54	34.83	34.67	34.83	43.16	26.95	27.48	24.11
m3	72.01	23.12	20.26	23.14	72.46	20.17	20.26	20.17	38.15	19.22	18.90	24.91
m4	77.09	25.85	27.41	25.85	77.82	27.49	27.41	27.49	46.34	26.06	25.74	26.45
m5	50.69	14.43	9.38	14.43	50.27	9.07	9.38	9.07	19.89	10.70	10.03	18.08
m6	75.76	25.71	28.02	25.71	76.65	27.89	28.02	27.89	46.34	26.05	25.96	26.24
m7	75.16	26.31	27.92	26.31	75.77	27.57	27.92	27.57	46.19	25.67	24.94	25.21
m8	65.12	22.75	25.12	22.75	65.27	24.89	25.12	24.89	39.29	19.60	19.54	21.61
m9	77.76	25.86	30.11	25.86	78.25	29.86	30.11	29.86	48.49	28.80	28.37	26.76

Table S6.

Cauchy pressures (CP_x, CP_y) and Lame's constants (λ, μ) for 10 models in Ge-Te based high-entropy chalcogenides.

Model	CP_x (GPa)	CP_y (GPa)	λ(GPa)	μ(GPa)
m0	-6.29	-14.70	18.812	24.534
m1	7.44	6.51	22.707	16.041
m2	7.72	-0.53	26.256	16.230
m3	1.04	-1.79	20.038	18.946
m4	1.35	-0.60	24.764	21.095
m5	-1.32	-3.65	10.363	12.362
m6	1.97	-0.53	24.933	20.732
m7	2.25	1.10	25.208	20.459
m8	5.52	1.14	22.767	16.623
m9	1.31	-0.90	25.981	21.430

Table S7.

Calculated bulk and shear modulus under Voigt and Reuss estimates (K_V, K_R, G_V, G_R), the universal anisotropic index (A^U), and the shear anisotropic factors (A_1, A_2, A_3) for ten models in Ge-Te based high-entropy chalcogenides.

Model	K_V (GPa)	K_R (GPa)	G_V (GPa)	G_R (GPa)	A^U	A_{comp}	A_{shear}	A_1	A_2	A_3
m0	37.415	33.261	27.971	21.104	1.752	0.059	0.140	1.227	1.539	1.023
m1	35.357	31.540	17.084	14.992	0.819	0.057	0.065	1.012	1.295	1.008
m2	41.533	39.877	22.013	10.460	5.564	0.020	0.356	2.305	3.051	0.991
m3	34.419	31.067	20.541	17.342	1.030	0.051	0.084	1.104	1.345	1.014
m4	40.305	38.054	23.684	18.495	1.462	0.029	0.123	1.519	1.860	1.025
m5	20.730	16.526	13.627	11.091	1.398	0.113	0.103	0.826	0.964	1.003
m6	40.220	38.156	23.460	17.992	1.574	0.026	0.132	1.577	1.931	1.039
m7	40.080	38.000	22.852	18.079	1.375	0.027	0.117	1.567	1.866	1.026
m8	35.021	33.068	18.609	14.643	1.413	0.029	0.119	1.447	1.784	1.018
m9	41.794	40.021	24.695	18.155	1.846	0.022	0.153	1.745	2.117	1.026

Table S8.

The minimum and maximum values of Young's modulus, E (GPa), linear compressibility, 1/K (TPa), shear modulus, G (GPa), and Poisson's ratio, η, for ten models in Ge-Te based high-entropy chalcogenides.

Model	E_{min}(GPa)	E_{max} (GPa)	$(1/K)_{min}$ (TPa)	$(1/K)_{max}$ (TPa)	G_{min} (GPa)	G_{max} (GPa)	η_{min}	η_{max}
m0	33.590	98.792	5.770	18.366	15.215	45.531	-0.047	0.663
m1	26.012	59.648	5.508	20.415	11.846	24.338	0.112	0.604
m2	17.905	97.594	4.790	15.307	6.632	44.533	-0.043	0.897
m3	29.573	71.819	6.328	19.476	13.295	30.680	0.055	0.605
m4	31.741	88.573	5.679	14.759	13.511	38.336	0.039	0.660
m5	17.255	47.774	8.974	41.468	8.067	20.299	0.003	0.582
m6	30.974	89.779	5.683	14.575	12.992	38.655	0.035	0.675
m7	31.043	85.484	5.679	14.648	13.199	36.717	0.055	0.655
m8	24.824	69.428	6.005	17.423	10.290	30.024	0.039	0.691
m9	31.180	94.906	5.640	13.555	12.933	41.785	0.030	0.690

Table S9.

The calculated sound velocities (transverse v_s and longitudinal v_l (using equations (**3**) and (**2**) respectively)), and Poisson's ratio (η) (using equation (***S*33**)).

Model	v_s(m/sec) equation (**3**)	v_l(m/sec) equation (**2**)	η equation (***S*33**)
m0	2294.731	4136.292	0.278
m1	1652.363	3511.251	0.358
m2	2182.391	3590.590	0.207
m3	1899.590	3676.881	0.318
m4	2172.528	3736.605	0.245
m5	1388.975	3023.178	0.366
m6	2167.394	3696.187	0.238
m7	2140.887	3663.314	0.241
m8	1862.226	3394.388	0.285
m9	2276.237	3740.239	0.206

Table S10.

Calculated melting temperature (T_{melt}), thermal expansion coefficient (α), the dominant phonon wavelength (λ_{dom}) at 300 K, the experimental lattice thermal conductivity $(\kappa_L)_{exp}$ from Jiang's work [36], and the calculated lattice thermal conductivities κ_L at 300 K for ten models in Ge-Te based high-entropy chalcogenides.

Model	T_{melt}∓300(K)	α(x10^{-5})	λ_{dom}(Å)	$(\kappa_L)_{exp}$ (W.m^{-1}.K^{-1}) [36]	*Mixed model* κ_L (W.m^{-1}.K^{-1})	*Slack model* κ (W.m^{-1}.K^{-1})
m0	677.94	6.52	1.020	1.75	1.6750	1.610
m1	613.26	9.98	0.796	1.74	0.5743	0.409
m2	637.59	9.86	0.793	0.70	0.5457	0.386
m3	627.26	8.45	0.878	0.90	0.9048	0.669
m4	654.78	7.59	0.911	0.65	0.9708	0.670
m5	535.91	12.95	0.692	0.58	0.5740	0.364
m6	650.79	7.72	0.902	0.66	0.9290	0.571
m7	648.77	7.82	0.892	0.65	0.8963	0.550
m8	608.30	9.62	0.801	0.51	0.6142	0.366
m9	660.02	7.47	0.916	0.60	0.9696	0.599

Table S11.

The calculated elastic constants C_{ij} (GPa) of $Pb_{0.99-y}Sb_{0.012}Sn_ySe_{1-2x}Te_xS_x$, (x=0.1, 0.2, 0.25, 0.3, 0.35, 0.4, 0.45), y = 0 solid solutions.

x	C_{11}	C_{12}	C_{13}	C_{21}	C_{22}	C_{23}	C_{31}	C_{32}	C_{33}	C_{44}	C_{55}	C_{66}
–	118.79	14.03	14.06	14.03	118.78	14.05	14.06	14.05	118.84	19.19	19.19	19.19
0.10	111.94	11.91	12.51	11.92	111.49	12.03	12.51	12.03	112.47	14.62	12.57	14.08
0.20	111.54	12.00	13.53	12.00	109.27	13.04	13.53	13.04	110.34	13.09	13.12	13.65
0.25	79.23	74.65	12.94	13.62	74.65	12.94	12.93	12.94	74.56	12.41	12.59	12.64
0.30	107.53	14.71	13.67	14.71	109.89	11.38	13.67	11.38	110.63	12.53	12.37	13.02
0.35	106.84	15.18	13.76	15.18	108.83	11.92	13.76	11.92	113.50	13.04	12.55	12.92
0.40	97.18	16.44	17.24	16.44	105.40	12.15	17.24	12.15	100.63	11.13	12.00	12.47
0.45	103.65	15.06	16.79	15.06	108.54	12.69	16.79	12.69	101.20	12.16	12.24	12.37

Table S12.

The theoretical density (ρ), the calculated sound velocity (longitudinal v_l, transverse v_s, and average v_m), Debye temperature Θ_D, melting temperature (T_{melt}), thermal expansion coefficient (α), and the dominant phonon wavelength (λ_{dom}) at 300 K for $Pb_{0.99-y}Sb_{0.012}Sn_ySe_{1-2x}Te_xS_x$ ((x=0.1, 0.2, 0.25, 0.3, 0.35, 0.4, 0.45) and y=0) solid solutions.

x	ρ(Kg/m^3)	v_s(m/sec)	v_l(m/sec)	v_m(m/sec)	Θ_D(K)	T_{melt}(K)	α($x10^{-5}$)	λ_{dom}(Å)
−	7948.338	1912.852	3312.138	2123.555	203.704	1255.1	5.502	0.890
0.10	7837.141	1740.780	3108.517	1937.552	185.177	1214.6	6.737	0.812
0.20	7743.496	1727.105	3107.808	1923.508	183.064	1212.2	6.927	0.806
0.25	7682.414	1539.551	2744.276	1713.329	162.616	1021.3	8.787	0.718
0.30	7638.208	1710.770	3103.872	1906.542	180.589	1188.5	7.157	0.799
0.35	7586.959	1723.359	3128.217	1920.643	181.500	1184.4	7.101	0.805
0.40	7534.427	1646.841	3043.342	1837.841	173.257	1127.3	7.830	0.770
0.45	7503.744	1682.989	3090.456	1877.298	176.720	1165.6	7.528	0.786

Table S13.

Young's modulus (E), bulk modulus (K), shear modulus (G), Poisson's ratio (η), Pugh's ratio (G/K), Vicker's hardness (H_V), Kleinman parameter(ζ), machinability index(μ_M), Cauchy pressures (CP_x, CP_y), and Lame's constants (λ, μ) for $Pb_{0.99-y}Sb_{0.012}Sn_ySe_{1-2x}Te_xS_x$ solid solutions (x=0.1, 0.2, 0.25, 0.3, 0.35, 0.4, 0.45) and y=0.

x	E(GPa)	K(GPa)	G(GPa)	η	G/K	H_V(GPa)	ζ	μ_M	CP (GPa)	λ(GPa)	μ(GPa)
−	72.694	48.418	29.083	0.250	0.601	5.606	0.269	2.523	-5.16	29.078	29.078
0.10	60.396	44.064	23.749	0.272	0.539	4.291	0.257	3.014	-2.71	28.322	23.741
0.20	58.973	43.993	23.098	0.277	0.525	4.084	0.258	3.361	-1.09	28.682	23.091
0.25	46.264	33.578	18.209	0.271	0.542	3.578	0.961	2.706	62.24	21.538	18.200
0.30	57.310	43.780	22.355	0.282	0.511	3.869	0.288	3.494	2.18	28.914	22.352
0.35	57.781	44.200	22.533	0.282	0.510	3.882	0.293	3.390	2.14	29.151	22.536
0.40	52.840	42.538	20.434	0.293	0.480	3.381	0.321	3.822	5.31	28.922	20.433
0.45	54.801	43.329	21.254	0.289	0.491	3.568	0.297	3.563	2.90	29.115	21.257

Table S14.

Calculated bulk and shear modulus under Voigt and Reuss estimates (K_V, K_R, G_V, G_R), the universal anisotropic index (A^U), and the percentage of anisotropy in compression and shear factors (A_{comp}, A_{shear}) for $Pb_{0.99-y}Sb_{0.012}Sn_ySe_{1-2x}Te_xS_x$ solid solutions (x=0.1, 0.2, 0.25, 0.3, 0.35, 0.4, 0.45) and y=0.

x	K_V (GPa)	K_R (GPa)	G_V (GPa)	G_R (GPa)	A^U	A_{comp}	A_{shear}
−	48.966	48.966	32.464	25.701	1.316	0	0.1163
0.10	45.422	45.351	28.215	19.283	2.318	0.0008	0.1881
0.20	45.367	45.323	27.477	18.719	2.340	0.0005	0.1896
0.25	34.154	33.526	20.122	16.295	1.193	0.0093	0.1051
0.30	45.284	45.263	26.804	17.906	2.485	0.0002	0.1990
0.35	45.653	45.639	26.923	18.144	2.420	0.0002	0.1948
0.40	43.875	43.792	24.277	16.591	2.318	0.001	0.1881
0.45	44.718	44.593	25.276	17.231	2.337	0.0014	0.1893

Table S15.

The minimum and maximum values of Young's modulus, E (GPa), linear compressibility, 1/K (TPa), shear modulus, G (GPa), and Poisson's ratio, η, for $Pb_{0.99-y}Sb_{0.012}Sn_ySe_{1-2x}Te_xS_x$ solid solutions (x=0.1, 0.2, 0.25, 0.3, 0.35, 0.4, 0.45) and y=0.

x	E_{min}(GPa)	E_{max} (GPa)	$(1/K)_{min}$ (TPa)	$(1/K)_{max}$ (TPa)	G_{min} (GPa)	G_{max} (GPa)	η_{min}	η_{max}
−	50.907	115.870	6.803	6.811	19.186	52.381	0.054	0.543
0.10	36.356	110.090	6.495	8.143	12.537	49.850	0.030	0.678
0.20	35.677	108.880	6.781	8.003	13.020	48.707	0.035	0.650
0.25	29.580	75.533	7.705	13.80	12.078	32.201	0.058	0.614
0.30	34.083	108.070	6.965	7.863	12.253	47.655	0.036	0.659
0.35	34.636	110.80	6.993	7.490	12.501	48.119	0.043	0.661
0.40	30.698	101.82	6.629	8.508	10.778	45.409	0.043	0.699
0.45	32.138	105.44	6.552	8.643	12.029	46.038	0.041	0.660

Table S16.

The calculated elastic constants C_{ij} (GPa) of $Pb_{0.99-y}Sb_{0.012}Sn_ySe_{1-2x}Te_xS_x$, (y=0.0, 0.05, 0.1, 0.15, 0.2, 0.25), x = 0.25.

**y**	C_{11}	C_{12}	C_{13}	C_{21}	C_{22}	C_{23}	C_{31}	C_{32}	C_{33}	C_{44}	C_{55}	C_{66}
0.00	79.23	74.65	12.94	13.62	74.65	12.94	12.93	12.94	74.56	12.41	12.59	12.64
0.05	105.83	12.76	13.22	12.76	109.18	11.41	13.22	11.41	105.19	12.77	12.68	13.17
0.10	87.10	9.86	99.67	12.12	90.55	11.02	12.15	11.05	92.46	14.21	11.98	12.55
0.15	96.95	11.46	11.98	11.46	97.69	10.70	11.98	10.70	91.19	11.65	12.56	12.77
0.20	75.16	16.04	15.49	16.04	97.47	8.78	15.49	8.78	88.29	11.79	11.51	11.53
0.25	68.56	16.62	17.72	16.62	87.37	11.17	17.72	11.17	86.60	11.24	10.54	11.42

Table S17.

Young's modulus (E), bulk modulus (K), shear modulus (G), Poisson's ratio (η), Pugh's ratio (G/K), Vicker's hardness (*H_V*), Kleinman parameter(ζ), and machinability index(μ_M) for $Pb_{0.99-y}Sb_{0.012}Sn_ySe_{1-2x}Te_xS_x$ solid solutions (y=0.0, 0.05, 0.1, 0.15, 0.2, 0.25) and x=0.25.

**y**	E(GPa)	K(GPa)	G(GPa)	η	G/K	H_V(GPa)	ζ	μ_M
0.00	46.264	33.578	18.209	0.271	0.542	3.578	0.961	2.706
0.05	57.079	42.574	22.357	0.277	0.525	3.990	0.271	3.334
0.10	55.547	36.999	22.223	0.250	0.601	4.634	0.264	2.604
0.15	52.501	38.318	20.643	0.272	0.539	3.886	0.269	3.289
0.20	48.108	37.039	18.741	0.284	0.506	3.377	0.365	3.142
0.25	44.585	36.089	17.227	0.294	0.477	2.975	0.393	3.211

Table S18.

Cauchy pressure (CP) and Lame's constants (λ, μ) for $Pb_{0.99-y}Sb_{0.012}Sn_ySe_{1-2x}Te_xS_x$ solid solutions (y=0.0, 0.05, 0.1, 0.15, 0.2, 0.25) and x=0.25.

**y**	CP (GPa)	λ(GPa)	μ(GPa)
0.00	62.24	21.538	18.200
0.05	-0.01	27.761	22.349
0.10	-4.35	22.219	22.219
0.15	-0.19	24.620	20.637
0.20	4.25	24.631	18.734
0.25	5.38	24.587	17.228

Table S19.

Calculated bulk and shear modulus under Voigt and Reuss estimates (K_V, K_R, G_V, G_R), the universal anisotropic index (A^U), and the percentage of anisotropy in compression and shear factors (A_{comp}, A_{shear}) for $Pb_{0.99-y}Sb_{0.012}Sn_ySe_{1-2x}Te_xS_x$ solid solutions (y=0.0, 0.05, 0.1, 0.15, 0.2, 0.25) and x=0.25.

y	K_V (GPa)	K_R (GPa)	G_V (GPa)	G_R (GPa)	A^U	A_{comp}	A_{shear}
0.00	34.154	33.526	20.122	16.295	1.193	0.0093	0.1051
0.05	43.887	43.860	26.577	18.136	2.328	0.0003	0.1889
0.10	40.211	39.566	25.766	18.055	2.225	0.0005	0.1821
0.15	39.343	39.158	24.174	17.112	2.068	0.0024	0.1711
0.20	37.949	37.602	21.673	15.808	1.864	0.0046	0.1565
0.25	37.060	36.303	19.774	14.679	1.756	0.0103	0.1479

Table S20. The calculated thermal (κ) and minimum thermal (κ_{min}) conductivities ($W \cdot m^{-1}. K^{-1}$) at 300 K, calculated lattice thermal conductivities κ_L ($W \cdot m^{-1}. K^{-1}$) at 300 K, κ_L contributed by acoustic phonons (κ_a), κ_L contributed by optical phonons (κ_o), and Grüneisen parameter γ_a for $Pb_{0.99-y}Sb_{0.012}Sn_ySe_{1-2x}Te_xS_x$ ((y=0.0, 0.05, 0.1, 0.15, 0.2, 0.25) and x=0.25) solid solutions.

y	*Clarke model* κ_{min} ($W.m^{-1}.K^{-1}$)	*Cahill model* κ_{min} ($W.m^{-1}.K^{-1}$)	*Slack model* κ ($W.m^{-1}.K^{-1}$)	*Mixed model* κ_L ($W.m^{-1}.K^{-1}$)	κ_a($W.m^{-1}.K^{-1}$)	κ_o ($W.m^{-1}.K^{-1}$)	γ_a
0.00	0.2996	0.2423	0.4517	0.6675	0.6051	0.0623	1.6028
0.05	0.3355	0.2723	0.5593	0.8659	0.7962	0.0697	1.6361
0.10	0.3344	0.2676	0.6518	0.9978	0.9278	0.0700	1.4989
0.15	0.3271	0.2647	0.5190	0.8067	0.7387	0.0680	1.6096
0.20	0.3158	0.2574	0.4195	0.6626	0.5972	0.0655	1.6750
0.25	0.3066	0.2517	0.3477	0.5583	0.4949	0.0634	1.7367

Table S21.

The theoretical density (ρ), the calculated sound velocity (longitudinal v_l, transverse v_s, and average v_m), Debye temperature Θ_D, melting temperature (T_{melt}), thermal expansion coefficient (α), and the dominant phonon wavelength (λ_{dom}) at 300 K for $Pb_{0.99-y}Sb_{0.012}Sn_ySe_{1-2x}Te_xS_x$ ((y=0.0, 0.05, 0.1, 0.15, 0.2, 0.25) and x=0.25) solid solutions.

y	ρ(Kg/m^3)	v_s(m/sec)	v_l(m/sec)	v_m(m/sec)	$\Theta_D(K)$	T_{melt}(K)	$\alpha(x10^{-5})$	λ_{dom}(Å)
0.00	7682.414	1539.551	2744.276	1713.330	162.616	1021.3	8.787	0.718
0.05	7584.821	1716.857	3089.204	1912.086	181.652	1178.5	7.157	0.801
0.10	7579.604	1712.293	2964.903	1900.906	181.550	1067.8	7.199	0.796
0.15	7365.038	1674.168	2989.951	1863.429	177.181	1126.0	7.751	0.781
0.20	7250.615	1607.715	2924.846	1792.069	170.436	997.2	8.537	0.751
0.25	7142.412	1553.039	2875.532	1733.404	164.946	958.2	9.288	0.726

References

1. Yao, H., Ouyang, L. & Ching, W. Ab Initio Calculation of Elastic Constants of Ceramic Crystals. **3204**, 3194–3204 (2007).

2. Yang, A., Bao, L., Peng, M. & Duan, Y. Explorations of elastic anisotropies and thermal properties of the hexagonal TMSi2 (TM = Cr, Mo, W) silicides from first-principles calculations. *Mater. Today Commun.* **27**, 102474 (2021).

3. Wang, A. J. *et al.* Structural and elastic properties of cubic and hexagonal TiN and AlN from first-principles calculations. *Comput. Mater. Sci.* **48**, 705–709 (2010).

4. Reuss, A. Berechnung der Fließgrenze von Mischkristallen auf Grund der Plastizitätsbedingung für Einkristalle . *ZAMM - J. Appl. Math. Mech. / Zeitschrift für Angew. Math. und Mech.* **9**, 49–58 (1929).

5. Hill, R. The elastic behaviour of a crystalline aggregate. *Proc. Phys. Soc. Sect. A* **65**, 349–354 (1952).

6. Korozlu, N., Colakoglu, K., Deligoz, E. & Surucu, G. First-principles study of structural, elastic, lattice dynamical and thermodynamical properties of GdX (X = Bi, Sb). *Philos. Mag.* **90**, 1833–1852 (2010).

7. Sun, Z., Music, D., Ahuja, R. & Schneider, J. M. Theoretical investigation of the bonding and elastic properties of nanolayered ternary nitrides. *Phys. Rev. B - Condens. Matter Mater. Phys.* **71**, 3–5 (2005).

8. Tian, Y., Xu, B. & Zhao, Z. Microscopic theory of hardness and design of novel superhard crystals. *Int. J. Refract. Met. Hard Mater.* **33**, 93–106 (2012).

9. Yousef, E. S., El-Adawy, A. & El-KheshKhany, N. Effect of rare earth (Pr2O3, Nd2O3, Sm2O3, Eu2O3, Gd2O3 and Er2O3) on the acoustic properties of glass belonging to bismuth-borate system. *Solid State Commun.* **139**, 108–113 (2006).

10. Varshney, D. & Shriya, S. Elastic, mechanical and thermodynamic properties at high pressures and temperatures of transition metal monocarbides. *Int. J. Refract. Met. Hard Mater.* **41**, 375–401 (2013).

11. Ranganathan, S. I. & Ostoja-Starzewski, M. Universal elastic anisotropy index. *Phys. Rev. Lett.* **101**, 3–6 (2008).

12. Ravindran, P. *et al.* Density functional theory for calculation of elastic properties of orthorhombic crystals: Application to TiSi2. *J. Appl. Phys.* **84**, 4891–4904 (1998).

13. Jiang, S. *et al.* Elastic and thermodynamic properties of high entropy carbide (HfTaZrTi)C and (HfTaZrNb)C from ab initio investigation. *Ceram. Int.* **46**, 15104–15112 (2020).

14. Boudiaf, K. *et al.* Structural, Elastic, Electronic and Optical Properties of LaOAgS-Type Silver Fluoride Chalcogenides: First-Principles Study. *J. Electron. Mater.* **46**, 4539–4556 (2017).

15. Guo, F. *et al.* Structural, mechanical, electronic and thermodynamic properties of cubic TiC compounds under different pressures: A first-principles study. *Solid State Commun.* **311**, 113856

(2020).

16. Anderson, O. L. a Simplified Method for Calculating the. *J. Phys. Chem. Solids* **24**, 909–917 (1963).

17. Wachter, P., Filzmoser, M. & Rebizant, J. Electronic and elastic properties of the light actinide tellurides. *Phys. B Condens. Matter* **293**, 199–223 (2001).

18. Naher, M. I. & Naqib, S. H. Structural, elastic, electronic, bonding, and optical properties of topological CaSn3 semimetal. *J. Alloys Compd.* **829**, 154509 (2020).

19. Naher, M. I. & Naqib, S. H. An ab-initio study on structural, elastic, electronic, bonding, thermal, and optical properties of topological Weyl semimetal TaX (X = P, As). *Sci. Rep.* **11**, 1–21 (2021).

20. Clarke, D. R. Materials selections guidelines for low thermal conductivity thermal barrier coatings. *Surf. Coatings Technol.* **163**–**164**, 67–74 (2003).

21. Clarke, D. R. & Levi, C. G. Materials design for the next generation thermal barrier coatings. *Annu. Rev. Mater. Res.* **33**, 383–417 (2003).

22. Cahill, D. G., Watson, S. K. & Pohl, R. O. Lower limit to the thermal conductivity of disordered crystals. *Phys. Rev. B* **46**, 6131–6140 (1992).

23. Cahill, D. G. *et al.* Nanoscale thermal transport. *J. Appl. Phys.* **93**, 793–818 (2003).

24. Morelli, D. T. & Slack, G. A. High lattice thermal conductivity solids. *High Therm. Conduct. Mater.* 37–68 (2006) doi:10.1007/0-387-25100-6_2.

25. Morelli, D. T. & Heremans, J. P. Thermal conductivity of germanium, silicon, and carbon nitrides. *Appl. Phys. Lett.* **81**, 5126–5128 (2002).

26. Arab, F., Sahraoui, F. A., Haddadi, K., Bouhemadou, A. & Louail, L. Phase stability, mechanical and thermodynamic properties of orthorhombic and trigonal MgSiN 2: An ab initio study. *Phase Transitions* **89**, 480–513 (2016).

27. JULIAN, C. L. Theory of Heat Conduction in Rare-Gas Crystals. *Phys. Rev.* **137**, (1965).

28. Bo Li, Yonghua Duan , Mingjun Peng, L. S. and H. Q. Anisotropic Elastic and Thermal Properties ofM2InX (M = Ti, Zr and X = C, N) Phases: A First-Principles Calculation. *Metals (Basel).* **12**, (2022).

29. Jia, T. Lattice thermal conductivity evaluated using elastic properties. *Phys. Rev. B* **95**, (2017).

30. Qin, G. *et al.* High-throughput computational evaluation of lattice thermal conductivity using an optimized Slack model. *Mater. Adv.* **3**, 6826–6830 (2022).

31. Toberer, E. S., Zevalkink, A. & Snyder, G. J. Phonon engineering through crystal chemistry. *J. Mater. Chem.* **21**, 15843–15852 (2011).

32. Fine, M. E., Brown, L. D. & Marcus, H. L. Elastic constants versus melting temperature in metals. *Scr. Metall.* **18**, 951–956 (1984).

33. Naher, M. I., Afzal, M. A. & Naqib, S. H. A comprehensive DFT based insights into the physical properties of tetragonal superconducting Mo5PB2. *Results Phys.* **28**, 104612 (2021).

34. K. Benkaddour, A. Chahed, A. Amar, H. Rozale, A. Lakdja, O. Benhelal, A. S. First-principles study of structural, elastic, thermodynamic, electronic and magnetic properties for the quaternary

Heusler alloys CoRuFeZ (Z = Si, Ge, Sn). *J. Alloys Compd.* (2016).

35. Liu, S. *et al.* Journal of the European Ceramic Society Phase stability , mechanical properties and melting points of high-entropy quaternary metal carbides from first-principles. *J. Eur. Ceram. Soc.* (2021) doi:10.1016/j.jeurceramsoc.2021.05.022.

36. Jiang, B. *et al.* High figure-of-merit and power generation in high-entropy GeTe-based thermoelectrics. **213**, 208–213 (2022).
